# Controlling catalytic activity and selectivity for partial hydrogenation by tuning the environment around active sites in iridium complexes bonded to supports†
†Electronic supplementary information (ESI) available: Plots of residuals and individual shells characterizing EXAFS data and fits, table summarizing goodness of fit parameters, characterization of the uncoated and IL-coated samples by XANES and by FTIR spectroscopy, SEM images and EDX spectra of IL-coated samples and calculated IL loadings, FTIR evidence of single-site iridium after IL coating, characterization of the samples before and after 1,3-butadiene hydrogenation reaction and time-on-stream data. See DOI: 10.1039/c8sc05287e


**DOI:** 10.1039/c8sc05287e

**Published:** 2019-01-09

**Authors:** Melike Babucci, Chia-Yu Fang, Jorge E. Perez-Aguilar, Adam S. Hoffman, Alexey Boubnov, Erjia Guan, Simon R. Bare, Bruce C. Gates, Alper Uzun

**Affiliations:** a Department of Chemical and Biological Engineering , Koç University , Rumelifeneri Yolu , Sariyer 34450, Istanbul , Turkey . Email: auzun@ku.edu.tr; b Koç University TÜPRAŞ Energy Center (KUTEM) , Koç University , Rumelifeneri Yolu , Sariyer 34450, Istanbul , Turkey; c Department of Materials Science and Engineering , University of California , Davis , California 95616 , USA; d Department of Chemical Engineering , University of California , Davis , California 95616 , USA . Email: bcgates@ucdavis.edu; e SSRL , SLAC National Accelerator Laboratory , Menlo Park , CA 94025 , USA; f Koç University Surface Science and Technology Center (KUYTAM) , Koç University , Rumelifeneri Yolu , Sariyer, 34450 Istanbul , Turkey

## Abstract

Single-site Ir(CO)_2_ complexes bonded to high-surface-area metal oxide supports, SiO_2_, TiO_2_, Fe_2_O_3_, CeO_2_, MgO, and La_2_O_3_, were synthesized by chemisorption of Ir(CO)_2_(acac) (acac = acetylacetonate) followed by coating with each of the following ionic liquids (ILs): 1-*n*-butyl-3-methylimidazolium tetrafluoroborate, [BMIM][BF_4_], 1-*n*-butyl-3-methylimidazolium acetate, [BMIM][Ac], and 1-(3-cyanopropyl)-3-methylimidazolium dicyanamide, [CPMIM][DCA]. Extended X-ray absorption fine structure spectroscopy showed that site-isolated iridium was bonded to oxygen atoms of the support. Electron densities on the iridium enveloped by each IL sheath/support combination were characterized by carbonyl infrared spectroscopy of the iridium *gem*-dicarbonyls and by X-ray absorption near-edge structure data. The electron-donor/acceptor tendencies of both the support and IL determine the activity and selectivity of the catalysts for the hydrogenation of 1,3-butadiene, with electron-rich iridium being selective for partial hydrogenation. The results resolve the effects of the IL and support as ligands; for example, the effect of the IL becomes dominant when the support has a weak electron-donor character. The combined effects of supports and ILs as ligands offer broad opportunities for tuning catalytic properties of supported metal catalysts.

## Abstract 2

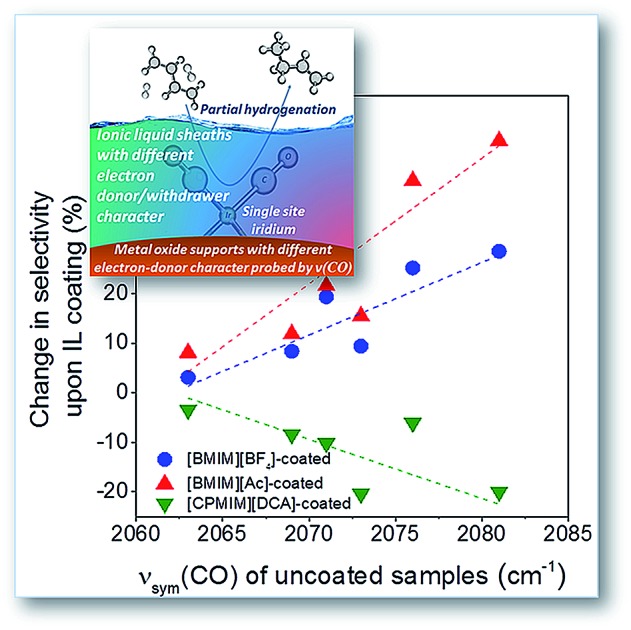
Enveloping atomically dispersed supported iridium with the choice of ionic liquid molecular sheaths and supports controls the catalytic performance.

## Introduction

Atomically dispersed metal catalysts on supports are drawing wide attention, because they offer new catalytic properties^1^–^5^ and an efficient use of expensive transition metals. Notwithstanding numerous recent investigations of structure and performance of such catalysts on supports such as metal oxides,^6^–^14^ there is only limited insight into how to control their catalytic properties. Some researchers have tuned the catalytic properties by choice of ligands bonded to the metal;^15^–^17^ the supports act as ligands as well.^18^–^20^ Electron-donor properties of supports are determined by the compositions and structures of the sites where the metals are bonded to the supports,^21^,^22^ and they can be tuned by promoters nearby on the support.^23^,^24^ The effects of supports are sometimes pronounced, as illustrated by rhodium complexes on HY-zeolite and on MgO—the strong electron-donor MgO induces high selectivity for 1,3-butadiene hydrogenation to butenes, whereas the zeolite does not.^25^

Molecules nearby metal centers on surfaces may also act as ligands, as illustrated by electron-donor ethylenediamine coatings of platinum nanowires,^23^ which influence the selectivity of the platinum for catalytic hydrogenation of nitroaromatics. The class of coatings influencing catalytically active metals in supported catalysts has recently been extended to include ionic liquids (ILs), which have been shown to enhance catalytic selectivity in cyclooctadiene hydrogenation.^26^ Many ILs are available, offering a wide range of electron-donor/acceptor properties. They can be quite stable on surfaces, and in addition to acting as ligands, they can also act as “molecular filters” controlling the local concentrations of reactants and intermediates.^26^–^29^

Early results show that 1,3-dialkylimidazolium-type IL coatings significantly influence the electronic properties of iridium carbonyls that are chemically bonded to alumina, Ir(CO)_2_/γ-Al_2_O_3_,^30^ and they thereby influence the catalyst performance for partial hydrogenation of 1,3-butadiene.^31^ Owing to a wide range of electron-donor/acceptor properties, the ILs offer broad prospects for tuning supported catalysts. We posit that they can be tuned to work synergistically with supports to control the electronic environments and thereby the catalytic properties of essentially molecular species on supports.^30^,^31^

Now we report such control for a family of site-isolated iridium complex catalysts by choice of the environment enveloping the metal—comprising both the support and a sheath of IL molecules—that regulate the chemical environment, and thereby the catalytic properties of the metal, while allowing the access of reactant molecules.

We assess the effects of the support and the IL in terms of their electron-donating/-withdrawing properties. Our data show how to dial-in the selectivity for partial hydrogenation of 1,3-butadiene to selectively give *n*-butenes. For example, the selectivity of these isolated Ir sites on porous silica incorporating the electron-withdrawing IL 1-(3-cyanopropyl)-3-methylimidazolium dicyanamide ([CPMIM][DCA]) was only 22%, but this value increased to 93% when the IL was switched to the electron-donating 1-*n*-butyl-3-methylimidazolium acetate ([BMIM][Ac]). Supports separately affect the catalytic properties,^3^,^32^–^38^ and we demonstrate how to measure the essential electron-donor/acceptor properties of the ILs and the supports acting in concert, by using Fourier-transform infrared (FTIR) and X-ray absorption near-edge structure (XANES) spectroscopies. Because there is an extremely large set of available ILs, the approach can be generalized to a large class of supported metal catalysts ranging from atomically dispersed species to nanoparticles.

Our goals were to learn how to control the catalytic properties of our single-site iridium catalyst by investigating a family of supports offering a wide range of electron-donor/acceptor tendencies. The supports (SiO_2_, TiO_2_ (anatase), Fe_2_O_3_, CeO_2_, MgO, and La_2_O_3_) and the ILs were chosen to offer a wide range of complementary electron-donor/acceptor tendencies (the ILs were the strong electron-acceptor [CPMIM][DCA], the strong electron-donor [BMIM][Ac], and one with intermediate electron-donor character, 1-*n*-butyl-3-methylimidazolium tetrafluoroborate, [BMIM][BF_4_]). We tested the catalysts for the industrially important partial hydrogenation of 1,3-butadiene.^39^–^42^ Our results show how the ILs and supports act synergistically as ligands to control the catalytic properties, and the spectroscopic data resolve the individual roles of each.

## Experimental

### Materials and methods

All sample handling and preparations were done with standard air-exclusion techniques. The chemicals were purchased from Sigma-Aldrich with the highest available purities. The metal oxide supports were calcined in flowing O_2_ with the temperature ramped at a rate of 3 K min^–1^ from room temperature to a selected final temperature (K) for each support: 793 for SiO_2_; 673 for TiO_2_ and CeO_2_; 773 for Fe_2_O_3_; 873 for La_2_O_3_; and 973 for MgO; thereafter, each sample was held at the final temperature for 5 h in flowing O_2_. Each IL, [BMIM][BF_4_], [BMIM][Ac], and [CPMIM][DCA], was dried at 353 K for 6 h and kept under vacuum for 12 h before being transferred into an argon-filled Labconco glove box. The supported catalysts were synthesized by the reaction of Ir(CO)_2_(acac), where acac is acetylacetonate ([C_5_H_7_O_2_]^–^), (18 mg) with a sample of each metal oxide (982 mg) to give an iridium loading of 1 wt% Ir in the final uncoated sample. The precursor and support were slurried in *n*-pentane, which had been purified in an MBraun SBS solvent purifier system. The slurry was mixed for 24 h in a sealed flask, followed by evacuation to remove the solvent and leave all the iridium on the support. Each of the resultant solid catalysts was stored in the argon-filled glove box. Coating of these samples with ILs involved impregnating the supported metal complex (200 mg) with the IL (100 mg) to adsorb the remaining unadsorbed IL, with the handling done in the glove box. Each of the resultant samples prepared as a physical mixture of IL-coated supported complexes with added support adsorbing the excess IL had an average grain diameter of approximately 0.03 to 0.05 mm and an iridium loading of 0.4 wt%. The IL loading of each sample was approximately 20 wt%, as shown by energy-dispersive X-ray spectroscopy (EDX) measurements shown in the ESI.†

### IR spectroscopy

A Bruker Vertex 80v spectrometer equipped with a vacuum sample chamber was used in transmission mode to measure FTIR spectra of solid samples with a spectral resolution of 2 cm^–1^. Samples were handled with the exclusion of moisture and air at room temperature under vacuum. Each sample was pressed between two KBr windows in a glove box and transferred to the sample chamber of the spectrometer immediately prior to the measurement. The sample chamber was evacuated within a few seconds after the insertion of the sample holder. For each reported spectrum, 256 scans were averaged, and before each of these measurements, a background of 128 scans was collected with the chamber under vacuum. Data characterizing [CPMIM][DCA]-coated catalysts were deconvoluted using a Voigt function to isolate the carbonyl bands from those associated with the IL.

### X-ray absorption spectroscopy (XAS)

X-ray absorption spectroscopy (XAS) measurements were made at beam line 9–3 at the Stanford Synchrotron Radiation Lightsource (SSRL). Beam line 9–3 is a collimating and focusing beam line with a 16-pole, 2 Tesla wiggler, Rh-coated harmonic-rejection mirrors, and a double-crystal, liquid-nitrogen cooled, Si(220) monochromator. Spectra were collected in fluorescence detection mode using N_2_-filled ion chambers and a 30-element Ge detector to measure signal. Approximately 250 mg of a sample was pressed into an *in situ* XAS cell^43^ in an argon-filled glove box and handled in the absence of air at the beam line to prevent contamination. An Ir black powder (Sigma-Aldrich) was scanned simultaneously with the sample for energy calibration. Extended X-ray absorption fine structure (EXAFS) spectra were measured from 200 eV below the Ir L_III_ edge (11 215 eV) to *k* = 15.3 Å^–1^ in 20 min, with three scans collected and averaged to improve the signal-to-noise ratio.

ATHENA software, a part of the IFEFFIT package,^44^ and the software XDAP^45^ were used in the analysis of the EXAFS data. Data alignment, edge calibration, and deglitching were performed with Athena. The edge, determined as the first inflection point of the absorption edge of the iridium black powder, was calibrated to the reported Ir L_III_ energy, 11 215.0 eV. XDAP was used for normalization, background subtraction, and data fitting,^45^ which allows the efficient application of a difference-file technique^46^ for the determination of optimized fit parameters and isolation of individual shells. Reference files, used in the data analysis, were calculated by using the code FEFF6.0.^47^

The crystal structure of Ir(CO)_2_(acac)^48^ was used to calculate the phase shifts and backscattering amplitudes representing the Ir–C_CO_, Ir–O_CO_, and Ir–O_s_ shells, as it was foreseen that the structure of the supported iridium species would be similar to that of this precursor. The Ir–C_CO_ and multiple scattering paths of the Ir–O_CO_ of the carbonyl were fit together to separate out the carbon and oxygen first shells. The phase shifts and backscattering amplitudes representing Ir–Si, Ir–Ti, Ir–Ce, Ir–Fe, and Ir–Mg shells were calculated from the structural parameters characterizing Ir–Si, Ir–Ti, Ir–Ce, Ir–Fe, and Ir–Mg alloys, respectively. The Ir–Si distance (3.42 Å) was found to be longer than typical Ir–Si (2.56 Å)^49^ distance whereas the Ir–Ti distance (2.56 Å) and the Ir–Ce distance (2.54 Å) were found to be shorter than typical Ir–Ti (2.69 Å)^50^ and typical Ir–Ce (3.05 Å)^51^ distances, respectively. These contributions might also represent an Ir–O* contribution (where O* is oxygen at long distance); however, the data quality was not sufficient to identify the back-scatterer or even to determine this contribution with confidence.

Iterative fitting was carried out for various structural models until the best agreement was attained between the calculated *k*^0^-, *k*^1^-, and *k*^2^-weighted EXAFS data and the postulated model. Plots were made to show the residuals remaining after the fitting; representative results are shown in the ESI.†

The fitting ranges in both *k*- and *R*-space, where *k* is the wave vector and *R* the distance from the absorbing Ir atom, in the analysis of the data characterizing the as-prepared sample were determined by the data quality; the range in *k* was in general between 3.5 ± 0.4 and 12.0 ± 0.6 Å^–1^, and the range in *R* was 0–3.5 Å for each sample. These values were used with the Nyquist theorem^52^ to estimate the justified number of fitting parameters. The accuracies of the parameters are estimated to be as follows (except for Ir–Si, Ir–Ti, Ir–Ce, Ir–Fe and Ir–Mg contributions): coordination number *N*, ±20%; distance *R*, ±0.02 Å; disorder term Δ*σ*^2^, ±20%; and inner potential correction Δ*E*_0_, ±20%.

Data analysis for XANES spectra was performed with ATHENA using standard procedures.^44^ The data for each sample were first calibrated using a known glitch in the monochromator observed in the *I*_0_ signal. A least-squares Gaussian fit of the glitch determined the error in the energy calibration of the samples to be 0.022 eV. The absorption edge energy was defined as the maximum of the first derivative of the normalized absorbance with respect to beam energy.

### Point of zero charge (PZC) measurements

PZC values characterizing the metal oxides were determined by using a Zetasizer Nanoseries instrument coupled with Malvern Multipurpose titrator. Each metal oxide (4 mg) was dispersed in a 0.1 M KNO_3_ solution (20 mL), and the solutions were ultrasonicated for approximately 5 min. Dispersed samples were titrated with 0.5 M NaOH and HNO_3_.^53^ pH increments of the instrument were set to 0.2 for measurements of SiO_2_ and TiO_2_. For back titration measurements, the pH increment was 0.1 for MgO, CeO_2_, and α-Fe_2_O_3_. Tolerance was set to 0.2 for SiO_2_ and TiO_2_ and to 0.05 for MgO, CeO_2_, and α-Fe_2_O_3_. Refractive index values used during measurements were 1.46, 2.90, 1.74, 2.20, and 2.94 for SiO_2_, TiO_2_, MgO, CeO_2_, and α-Fe_2_O_3_, respectively. Absorbance values used during the measurements were 0.01, 1.0, 0.1, 0.01, and 0.1 for SiO_2_, TiO_2_, MgO, CeO_2_, and α-Fe_2_O_3_, respectively. The PZC characterizing La_2_O_3_ was taken to be a literature value, 10.3 (pH).^54^

### Scanning electron microscopy coupled with energy-dispersive X-ray spectroscopy (SEM/EDX)

A Zeiss Ultra Plus scanning electron microscope (SEM) equipped with a field-emission gun was used to collect images of the catalyst samples in secondary-electron mode. Powder samples consisting of IL-coated supported iridium complexes on SiO_2,_ TiO_2_, α-Fe_2_O_3_, CeO_2_, MgO, and La_2_O_3_ were mounted on carbon tape to minimize support charging effects. Images were collected at magnifications of 20k×, 50k×, and 100k× with an accelerating voltage of 3 kV for each sample. The working distances between the probe of the microscope and samples were in the range of 2.6–2.8 mm. Each EDX image was collected at a magnification in the range of 10–75k× with an accelerating voltage of 10 kV and a working distance in the range of 5.2–5.4 mm.

### Catalyst performance measurements

To evaluate the performance of each catalyst, uncoated and IL-coated supported iridium complexes (each including 300–350 mg of the supported iridium *gem*-dicarbonyl complex, independent of whether an IL coating was present) were each placed in a 1/4-inch (OD) stainless-steel once-through tubular flow reactor. A Thermcraft three-zone resistively heated furnace (Model number XST-3-0-18-3V) equipped with PC-operated temperature controllers was used for temperature control of the reactor. Electronic mass flow controllers (Aalborg, Model GFC17) were used to control the flow rates of feed gases. Before each measurement, the catalysts were treated in flowing ethylene at 373 K for 1 h as the temperature was ramped at a rate of 3 K min^–1^ to convert the almost inactive supported Ir(CO)_2_ complexes into mixtures of Ir(CO)_2_(C_2_H_4_) and Ir(CO)_2_(C_2_H_4_)_2_ complexes, as reported for the isostructural HY-zeolite-supported iridium complexes.^37^ Then, the flow of the reaction mixture with a molar ratio of H_2_ (Linde, 99.99 vol%) to 1,3-butadiene (Linde, 99.6 vol%) of 2 : 1 was started through the catalyst bed, operated at atmospheric pressure and 333 K. Products were analysed with an online gas chromatograph (Agilent 7890A) equipped with a GS-alumina column (50 m × 530 μm) and a flame-ionization detector. Conversions of 1,3-butadiene were in the differential range, <2%. Reaction rates are reported per Ir atom in terms of turnover frequency (TOF), assuming that all of the Ir atoms were accessible for reaction. Repeat measurements showed that the TOF values are reproducible within ±2%. Control experiments done with catalysts containing double the loading of IL were performed to verify that the intraparticle mass transfer limitations were not significant.

## Results

### Atomically dispersed supported iridium complexes

Supported iridium complexes were synthesized by the reaction of Ir(CO)_2_(acac) with freshly calcined metal oxides. The samples incorporated 1 wt% Ir, giving an average distance between Ir atoms of at least 1 nm, depending on the support surface area, to assure site-isolation. Consistent with the atomic dispersion, Ir L_III_-edge EXAFS spectra of the samples (Tables 1 and S1, Fig. S1–S36 in the ESI†) give no evidence of an Ir–Ir scattering path that would represent metal clusters/nanoparticles, consistent with atomically dispersed iridium in each catalyst.^55^ The best-fit EXAFS model characterizing each sample includes an Ir–O_s_ scattering path (the subscript refers to the support) with a coordination number of 2.2 ± 0.2 (Table 1) and a bonding distance ranging from 2.01 to 2.07 Å, as expected for positively charged group-8 metals on metal oxides.^46^ The data in Table 1 indicate that each Ir atom was bonded to approximately two carbon atoms (indicated by a coordination number of 2.1 ± 0.2) at a distance ranging from 1.90 to 2.02 Å, which is typical of Ir–C bonding in iridium dicarbonyl complexes.^56^

**Table 1:** Summary of EXAFS fit parameters characterizing samples prepared by adsorption of Ir(CO)_2_(acac) on metal oxides having various electron-donor characteristics (quantified by the their corresponding PZC values)^*a*^

Sample (PZC of support, pH)	Shell^*b*^	*N*	*R* (Å)	Δ*σ*^2^ × 10^3^ (Å^2^)	Δ*E*_0_ (eV)	*k* range (Å^–1^)	*R* range (Å)
Ir(CO)_2_/SiO_2_ (1.7)	Ir–O_s_	2.25	2.07	3.46	–3.42	3.82–12.43	0.5–3.50
	Ir–C_CO_	2.25	2.00	3.87	–1.61		
	Ir–O_CO_	2.00	3.28	7.78	–5.12		
	Ir–Si	2.50	3.42	5.94	5.01		
Ir(CO)_2_/TiO_2_ (4.5)	Ir–O_s_	2.40	2.03	0.10	4.21	3.17–12.10	0.5–3.00
	Ir–C_CO_	2.20	1.92	1.55	–0.64		
	Ir–O_CO_	2.10	2.95	5.00	5.66		
	Ir–Ti	1.40	2.65	6.21	–8.00		
Ir(CO)_2_/Fe_2_O_3_ (6.8)	Ir–O_s_	2.40	2.01	0.10	3.54	3.92–12.30	0.5–3.20
	Ir–C_CO_	2.14	1.90	2.80	8.00		
	Ir–O_CO_	1.90	2.91	6.89	–1.70		
	Ir–Fe	1.12	2.52	7.69	–5.48		
Ir(CO)_2_/CeO_2_ (8.7)	Ir–O_s_	2.30	2.02	0.10	–3.75	3.92–12.60	0.5–3.2
	Ir–C_CO_	2.10	1.90	3.73	8.00		
	Ir–O_CO_	1.90	2.83	6.54	–7.73		
	Ir–Ce	2.20	2.54	10.17	–8.00		
Ir(CO)_2_/MgO (9.8)	Ir–O_s_	2.40	2.06	0.10	–6.98	4.02–11.30	0.5–3.40
	Ir–C_CO_	2.20	2.02	0.62	0.29		
	Ir–O_CO_	2.00	2.95	5.78	–8.00		
	Ir–Mg	1.40	2.76	0.10	7.36		
Ir(CO)_2_/La_2_O_3_ (10.3)	Ir–O_s_	2.00	2.02	0.10	1.76	3.15–11.30	0.5–3.20
	Ir–C_CO_	1.89	1.93	3.16	4.26		
	Ir–O_CO_	2.14	2.92	5.98	8.00		
	Ir–O_l_	2.10	2.46	0.10	0.21		

^*a*^Notation: *N*, coordination number; *R*, distance between the absorber and backscatterer atoms; Δ*σ*^2^, mean square relative displacement; Δ*E*_0_, inner potential correction. Error bounds are estimated as follows: *N*, ±20%; *R*, ±0.02 Å; Δ*σ*^2^, ±20%; Δ*E*_0_, ±20%.

^*b*^O_s_ and O_l_ denote surface oxygen atoms of supports; C_CO_ and O_CO_ denote carbon and carbon and oxygen atoms of carbonyl ligands bonded to iridium, respectively.

**Fig. 1:**
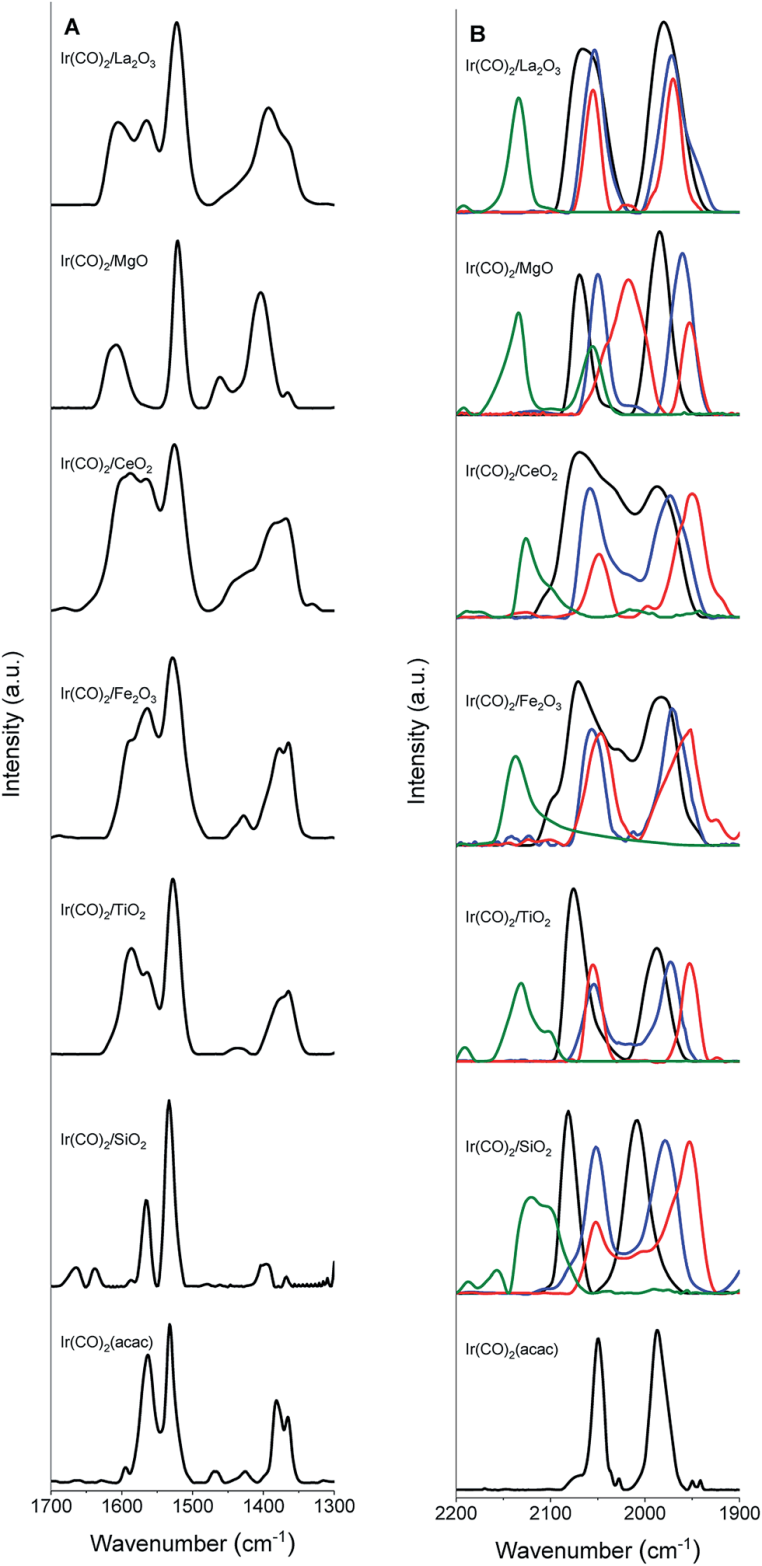
IR spectra of uncoated (black) and IL-coated ([BMIM][BF_4_] (blue), [BMIM][Ac] (red), and [CPMIM][DCA] (green)) Ir(CO)_2_ complexes supported on SiO_2_, TiO_2_, Fe_2_O_3_, CeO_2_, MgO, and La_2_O_3_ in the region of (A) 1300–1700 cm^–1^ and (B) 1900–2200 cm^–1^.

IR spectra (Fig. 1) confirm the identification of iridium *gem*-dicarbonyls. We observed no bridging CO bands that would have indicated the presence of iridium clusters (details in the ESI, Section S2.1†).^57^ A comparison of the IR spectra of the Ir(CO)_2_(acac) precursor and the supported samples (Fig. 1, Tables 2 and S2†) shows that when Ir(CO)_2_(acac) was adsorbed, some bands shifted or were replaced, as expected,^56^ for the formation of the supported iridium *gem*-dicarbonyls (details in the ESI, Section S2.1†).

**Table 2:** *ν*_sym_(CO) and *ν*_asym_(CO) band positions characterizing Ir(CO)_2_(acac) and uncoated and IL-coated metal-oxide-supported Ir(CO)_2_ complexes

Supported Ir(CO)_2_ complex	*ν*_sym_(CO) (cm^–1^)	*ν*_asym_(CO) (cm^–1^)
Ir(CO)_2_(acac)	2049	1985
Ir(CO)_2_(acac) in hexane^56^	2074	2000
Ir(CO)_2_/SiO_2_	2081	2008
Ir(CO)_2_/TiO_2_	2076	1988
Ir(CO)_2_/Fe_2_O_3_	2073	1981
Ir(CO)_2_/CeO_2_	2071	1982
Ir(CO)_2_/MgO	2069	1985
Ir(CO)_2_/La_2_O_3_	2066	1977
[BMIM][BF_4_]-coated Ir(CO)_2_/SiO_2_	2052	1981
[BMIM][BF_4_]-coated Ir(CO)_2_/TiO_2_	2055	1971
[BMIM][BF_4_]-coated Ir(CO)_2_/Fe_2_O_3_	2056	1969
[BMIM][BF_4_]-coated Ir(CO)_2_/CeO_2_	2057	1974
[BMIM][BF_4_]-coated Ir(CO)_2_/MgO	2058	1960
[BMIM][BF_4_]-coated Ir(CO)_2_/La_2_O_3_	2055	1970
[BMIM][Ac]-coated Ir(CO)_2_/SiO_2_	2049	1953
[BMIM][Ac]-coated Ir(CO)_2_/TiO_2_	2052	1962
[BMIM][Ac]-coated Ir(CO)_2_/Fe_2_O_3_	2053	1947
[BMIM][Ac]-coated Ir(CO)_2_/CeO_2_	2050	1966
[BMIM][Ac]-coated Ir(CO)_2_/MgO	2056	1952
[BMIM][Ac]-coated Ir(CO)_2_/La_2_O_3_	2053	1970
[CPMIM][DCA]-coated Ir(CO)_2_/SiO_2_	2098	1974
[CPMIM][DCA]-coated Ir(CO)_2_/TiO_2_	2084	1956
[CPMIM][DCA]-coated Ir(CO)_2_/Fe_2_O_3_	2081	1973
[CPMIM][DCA]-coated Ir(CO)_2_/CeO_2_	2074	1955
[CPMIM][DCA]-coated Ir(CO)_2_/MgO	2064	1959
[CPMIM][DCA]-coated Ir(CO)_2_/La_2_O_3_	2061	1991

### Supports as ligands

The *ν*(CO) spectra (Table 2) distinguish one support from another as electron-donor/acceptor ligands. The major *ν*_sym_(CO) peaks indicate Ir(CO)_2_ on the surface.^56^

**Fig. 2:**
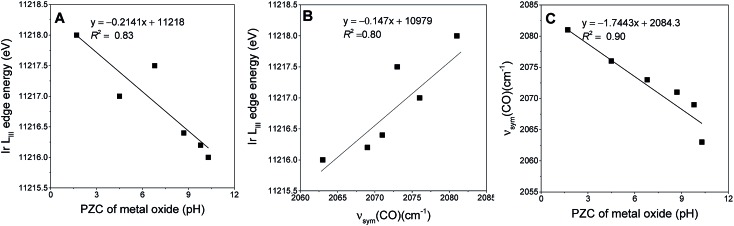
Measures of the electron-donor strengths of the supports: variation of (A) Ir L_III_ edge energy of the uncoated supported iridium carbonyls with PZC of the support; (B) Ir L_III_ edge position with the corresponding *ν*_sym_(CO) band positions of uncoated supported iridium carbonyls; and (C) *ν*_sym_(CO) band positions of uncoated supported iridium carbonyls with PZC of the support.

Electronic environments of the supported iridium were probed with XANES spectroscopy at the Ir L_III_ edge (Fig. S37–S43 in the ESI†). Table S3 in the ESI† is a summary of the absorption edge energies of each uncoated sample. This energy decreased from 11 218.0 to 11 216.0 eV in the order SiO_2_, TiO_2_, Fe_2_O_3_, CeO_2_, MgO, and La_2_O_3_ indicating the increasing electron density on iridium donated by the support.^37^ This order is consistent with the PZC values characterizing the metal oxides (Fig. 2A).^53^,^54^ The IR *ν*_sym_(CO) band positions of the iridium carbonyls provide still another, easily determined, measure of the electron-donor strengths of the supports, as confirmed by the strong correlation between the edge energies of the iridium carbonyls and their *ν*_sym_(CO) band positions as shown in Fig. 2B (or between *ν*_sym_(CO) and PZC of the support (Fig. 2C)). Thus, for example, a red shift in the *ν*_sym_(CO) band position indicates an increase in the electron density on the iridium sites.^58^ The data show variations in broadness of these *ν*(CO) bands on various supports, originating from differences in the degrees of surface uniformity of these metal oxides. The broad bands and shoulders that were observed for Ir/CeO_2_ and Ir/Fe_2_O_3_ indicate iridium carbonyls on minority surface sites.

### ILs distributed uniformly on supports

IL loadings were chosen to be 20 wt%, sufficient to provide a multilayer covering of all Ir sites in each sample but not so much as to present a significant transport limitation to the reactant molecules in catalysis.^26^ The loading corresponds to 45 ± 3 IL molecules per Ir site, on average. The IL was present in layers with a thickness on average of several molecules. On a macroscopic scale, at least, the ILs were uniformly distributed over the supported species, as shown by SEM/EDX images (Fig. S44–S50 and Table S4 in the ESI; details in the ESI†).

### ILs as ligands

FTIR spectra of the various samples and the bulk ILs (Fig. 1 and S51–S53 in the ESI†) show that the supported complexes were still present as site-isolated iridium *gem*-dicarbonyls after addition of the IL, as shown by the two distinct carbonyl stretching bands of the iridium *gem*-dicarbonyls in each supported catalyst. The IL coatings shift these bands. For example (Fig. 1B and Table 2) the *ν*_sym_(CO) and *ν*_asym_(CO) bands of Ir(CO)_2_/SiO_2_, at 2081 and 2008 cm^–1^, were shifted to 2052 and 1981 cm^–1^, respectively, upon coating with [BMIM][BF_4_]. Similarly, when the complexes were coated with [BMIM][Ac], the bands were red-shifted by 32 and 55 cm^–1^, respectively. These shifts confirm^28^,^29^,^59^ that these ILs donate electrons, acting as ligands. In contrast, the electron-withdrawing IL [CPMIM][DCA], with its cyano group on the cation, blue-shifted the corresponding bands to 2098 and 1974 cm^–1^. Doubling the IL loadings led to no significant changes in the band shifts, demonstrating that ILs were present at saturation loadings in terms of their ligand effects.^30^

To rule out the possibility that the iridium complex was removed from the surface and dissolved in the IL coating, we washed some IL-coated samples in methanol and checked the FTIR spectra of both the filtrates and the washed samples. No vibrational bands associated with iridium carbonyls were detected in the filtrates, and the spectra of the washed samples still indicated the presence of the iridium carbonyls (although the peak positions and shapes were changed by the treatments, as illustrated in Fig. S54 in the ESI† for [BMIM][BF_4_]-coated Ir(CO)_2_/MgO). These results show that the iridium complexes remained bonded to the support even after the samples were coated with the ILs and did not dissolve in the IL.

### Catalytic hydrogenation of 1,3-butadiene

The data presented above show that the electronic environments of the Ir centers are influenced both by the support and the IL. Thus, we tested the influence of each one on the catalytic properties for the hydrogenation of 1,3-butadiene. First, each catalyst was activated by removal of at least one of the CO ligands per Ir atom by treatment in flowing ethylene at 373 K; this treatment incorporated ethylene ligands in place of CO, which is a strong catalytic reaction inhibitor,^31^ as reported for similar metal carbonyl complexes supported on metal oxides and zeolites. The relative concentrations of species and the FTIR spectra before and after ethylene exposure are provided in Table S5 and Fig. S55–S77 in the ESI.† FTIR spectra recorded before and after ethylene exposure (Fig. S55–S77 in the ESI†), indicate changes in the intensities and positions of the *ν*(CO) bands as well as the formation of new bands, but none indicating the formation of carbonyls bonded to neighbouring iridium centres, which would have indicated iridium cluster formation.^31^ New bands located between the two carbonyl stretching bands are consistent with the partial replacement of CO with ethylene.^60^,^61^ For example, in the spectrum of the TiO_2_-supported iridium complex coated with [BMIM][BF_4_], the intensity of the *ν*_asym_(CO) band at 1988 cm^–1^ decreased slightly relative to that of the *ν*_sym_(CO) band, accompanied by the appearance of two new bands, at 2030 and 2048 cm^–1^, assigned to Ir(CO)(C_2_H_4_) and Ir(CO)(C_2_H_4_)_2_, respectively.^31^ The areas of the *ν*(CO) bands indicate that the supported complexes were converted into mixtures of Ir(CO)_2_ (∼74%), Ir(CO)(C_2_H_4_) (∼16%), and Ir(CO)(C_2_H_4_)_2_ (∼10%) on TiO_2_, for example. Data characterizing other samples indicate the presence of mixtures of Ir(CO)_2_, Ir(CO)(C_2_H_4_), and Ir(CO)(C_2_H_4_)_2_ in varying concentrations (Table S5†). Following the activation of each supported iridium complex by reaction with ethylene, it was cooled to 333 K in flowing helium followed by the start of flow of the reactant stream consisting of H_2_ + 1,3-butadiene in a molar ratio of 2 : 1 at a space velocity chosen to ensure differential conversions of the 1,3-butadiene (<2%). Typical data (Fig. S78 in the ESI†) characterizing [BMIM][BF_4_]-coated iridium on SiO_2_ for 3 h on stream show that the product distribution and 1,3-butadiene conversion of *ca.* 1.5% were essentially constant.

**Fig. 3:**
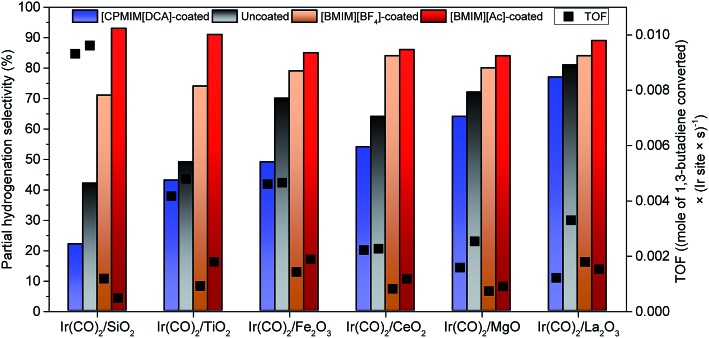
Partial hydrogenation selectivity (sum of all butene isomers: *trans*-2-butene, 1-butene, and *cis*-2-butene) (colored bars) and TOF (black symbols) characterizing each catalyst, measured at steady state and under differential conversion conditions (<2%) at an H_2_/BD feed molar ratio of 2.0 at 333 K and 1 atm catalyzed by activated uncoated and IL-coated iridium complexes supported on metal oxides with various electron-donor characteristics.

Table S6† is a summary of the TOF and selectivity data characterizing each catalyst, and Fig. 3 illustrates the changes in total partial hydrogenation selectivity of the catalysts with changes in electron-donor/acceptor properties of the supports and the ILs; the catalyst performance data were measured at differential conversions, <2%. The data representing uncoated SiO_2_-supported iridium complexes, for example, indicate a TOF of 9.6 × 10^–3^ (mol of 1,3-butadiene converted) × (Ir site × s)^–1^ (Table S6†) with a selectivity for butenes (sum of 1-butene (21%), *trans*-2-butene (11%), and *cis*-2-butene (10%)) of approximately 42% (Fig. 3). Such a low selectivity for partial hydrogenation is typical of iridium complexes on supports such as SiO_2_ that are not good electron-donor ligands. In contrast, when the iridium complexes were supported on good electron-donor supports such as La_2_O_3_, the selectivity for butenes was approximately twice as high (81%) (Fig. 3). For comparison, Table S6† and Fig. 3 also include the corresponding performance data characterizing the IL-coated catalysts—showing that the IL layer strongly influences the catalytic performance. The TOF values characterizing the coated catalysts were lower than those characterizing the respective uncoated catalysts. The partial hydrogenation selectivity, on the other hand, increased when the ILs incorporated [BMIM]^+^-ions and decreased when the IL incorporated [CPMIM]^+^-ions (Fig. 3). For example, under our conditions, the TOF characterizing the SiO_2_-supported iridium complex decreased from 9.6 × 10^–3^ to 1.2 × 10^–3^ (mole of 1,3-butadiene converted) × (Ir site × s)^–1^, whereas the partial hydrogenation selectivity increased from 42 to 73% as a result of the deposition of [BMIM][BF_4_]. Data presented in Table S6† and Fig. 3 also indicate that the effect of the IL coating on the catalytic performance depends strongly on the type of support.

## Discussion

**Fig. 4:**
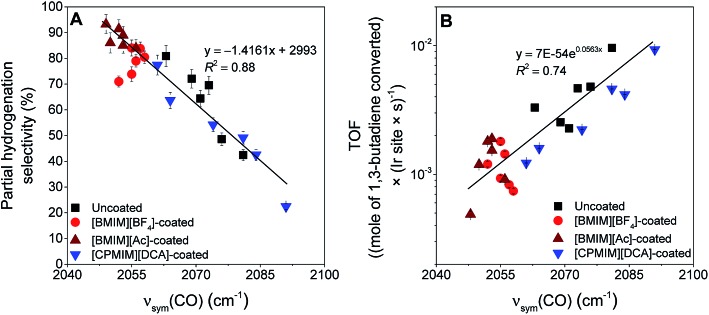
Variation of (A) selectivity for partial hydrogenation (sum of all butene isomers) and (B) TOF of 1,3-butadiene with the *ν*_sym_(CO) band position of the corresponding coated and uncoated iridium complexes supported on metal oxides, following activation. Black symbols indicate uncoated samples, and red, dark red, and blue symbols represent [BMIM][BF_4_]-, [BMIM][Ac]-, and [CPMIM][DCA]-coated supported complexes, respectively.

The essential results are: (a) that both the support and the IL act as ligands determining the electronic properties of the metal, which is represented simply by the *ν*_sym_(CO) band positions, and (b) that the electronic properties control the catalytic selectivity and activity (TOF) for partial hydrogenation of 1,3-butadiene. All data are accounted for by the correlations of Fig. 4. Thus, when the electron density on the iridium is the greatest, the selectivity for partial hydrogenation of 1,3-butadiene is maximized, and *vice versa* (Fig. 4A). On the other hand, when the electron density on iridium is the greatest, the TOF is minimized, and *vice versa* (Fig. 4B).

The deviations from the trend lines in Fig. 4 might originate from (i) non-uniformity of the active sites associated with the heterogeneity of the support surfaces and/or (ii) undetermined differences in the behavior of the ILs on various supports. Presence of this second effect was illustrated in a report focusing on the interactions of [BMIM][BF_4_] with various metal oxides in the absence of any active metal. These interactions vary considerably with the changes in the electron-donor character of the supports and lead to variations in the thermal stability of [BMIM][BF_4_] on these metal oxides.^53^ The interactions can also alter other physical and chemical characteristics of the IL when it is in contact with a metal oxide. Consequently, solubilities of guest molecules in a specific IL—thus, a filter effect^26^—can vary significantly when an IL is dispersed on various supports. Because the IL filter effect controls the concentrations of the reactants and intermediates interacting with the active sites, it influences the catalytic performance, and this effect explains the deviations from a perfect correlation in Fig. 4. Thus, we recognize the potential inclusion of an additional structural parameter that could be tuned to improve the catalytic performance.

**Fig. 5:**
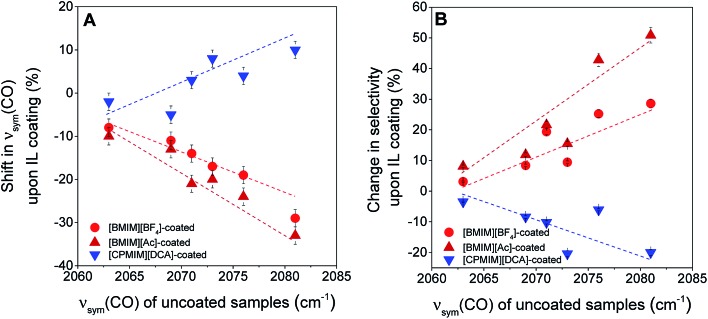
(A) Variations in the magnitudes of *ν*_sym_(CO) band shifts observed upon coating the supported metal complexes with various ILs. The *x*-axis quantifies the electron-donor character of the supports, and the vertical axis represents the magnitude of the ligand effect of the IL; (B) variation in the change of partial hydrogenation selectivity upon the deposition of various ILs, [BMIM][BF_4_], [BMIM][Ac], and [CPMIM][DCA] with *ν*_sym_(CO) band positions on the corresponding uncoated supported iridium complexes, quantifying the electron-donor character of the support.

The electronic effects shown in Fig. 4 are substantial, but, on some supports the effects of the electron-donor/acceptor properties of the IL are small, whereas on others they are at least twice as large. For example, when the iridium complex is supported on the strongly electron-donating La_2_O_3_, the effect of the IL is indicated by shifts <10 cm^–1^ in the *ν*_sym_(CO) band position (Fig. 5A). In contrast, when the support is the weak electron-donor SiO_2_, the magnitude of the shift indicating the ligand effect of the IL exceeded 20 cm^–1^ when the IL sheath contained the [BMIM]^+^ ion (a red shift) and the [CPMIM]^+^ ion (a blue shift). Fig. 5A illustrates the magnitudes of these shifts, and thus the strength of the ILs' ligand effects compared with those of the supports.

To further investigate the ligand effects on catalysis, we considered the change in catalytic selectivity for butenes as a function of the electron-donor character of the support. Fig. 5B shows that the increase in partial hydrogenation selectivity upon the deposition of [BMIM]^+^-containing IL sheaths becomes dominant when the support is a weak electron-donor, but when the IL incorporates a cyano group, the catalyst becomes less selective for partial hydrogenation, because (as illustrated in Fig. 5A), this IL withdraws electrons from iridium. As shown in Fig. 5B, this effect is dominant when the support is a weak electron-donor, as in the case of [BMIM]^+^-containing ILs, but presenting a correlation with a reverse trend.

Although there is more to learn about the interactions between the IL and the catalytic species, the correlations shown here make clear that the IL, like a support, acts as a ligand. Moreover, the data confirm that these ligand effects are additive, providing a high degree of flexibility for controlling the electronic structures of catalytic sites with an enormous number of possible combinations presenting opportunities for tuning the electronic environments of supported metals over wide ranges. We posit that the opportunities offered by enveloping the catalytically active species with IL sheaths/supports for tuning the catalytic properties extend to many metals, supports, and ILs, and recent results characterizing nickel nanoparticles on supports incorporating ILs^42^ indicate that the opportunities extend beyond atomically dispersed metals to metal nanoparticles.

## Conclusions

Atomically dispersed supported iridium complexes were synthesized on metal oxides having a wide range of surface electron-donor characteristics, SiO_2_, TiO_2_, Fe_2_O_3_, CeO_2_, MgO, and La_2_O_3_, and coated with various ILs, [BMIM][BF_4_], [BMIM][Ac], and [CPMIM][DCA]. The electron density on the iridium was probed with FTIR spectroscopy determining the *ν*(CO) frequencies. The results show that the electron-donor/acceptor character of the support and that of the IL sheath together determine the electronic structures of the iridium complexes and their catalytic properties for hydrogenation of 1,3-butadiene, for which high selectivities for butenes were obtained. Thus, the metal oxide support works as a ligand that influences the electron density on the metal sites. As the electron-donor strength of the metal oxides increases, as probed by an increase in the corresponding PZC, the isolated supported iridium sites become more electron-rich and consequently become more selective as catalysts for the formation of butenes from 1,3-butadiene. The data illustrate a similar ligand effect of the IL sheaths, which becomes dominant when the support is a weak electron-donor. Among the ILs considered, those incorporating [BMIM]^+^ donate substantial electron density to the metal sites, making them more selective as catalysts for butene formation, whereas when the cation incorporates a cyano group, the IL withdraws electrons from the metal sites and makes them less selective for partial hydrogenation. The strategy of enveloping the catalytically active species with IL sheaths/supports provides a high degree of flexibility for controlling the electronic structures of these species with, in prospect, an enormous number of possible combinations, presenting opportunities for tuning the electronic environments and the corresponding catalytic activity, selectivity, and stability.

## Conflicts of interest

There are no conflicts to declare.

## Supplementary Material

Supplementary information
