# Supplementary material for: Controlling catalytic activity and selectivity for partial hydrogenation by tuning the environment around active sites in iridium complexes bonded to supports
Source: Chem Sci. 2019 Jan 9;10(9):2623–32. doi: 10.1039/c8sc05287e (PMC6419936; doi:10.1039/c8sc05287e)
Supplement: Supplementary file 1 [file SC-010-C8SC05287E-s001.pdf]

Electronic Supplementary Material (ESI) for Chemical Science.
This journal is © The Royal Society of Chemistry 2019

ELECTRONIC SUPPLEMENTARY INFORMATION

Controlling catalytic activity and selectivity for partial hydrogenation by tuning environment around active sites in iridium complexes bonded to supports

Melike Babucci,[†,‡] Chia-Yu Fang,[§] Jorge E. Perez-Aguilar,[‖] Adam S. Hoffman,[#] Alexey Boubnov,[#] Erjia Guan,[§] Simon R. Bare,[#] Bruce C. Gates,[‖,]* and Alper Uzun[†,‡,⊥]*

[†]*Department of Chemical and Biological Engineering, Koç University Rumelifeneri Yolu, Sariyer 34450, Istanbul, Turkey*

[‡]*Koç University TÜPRAŞ Energy Center (KUTEM), Koç University Rumelifeneri Yolu, Sariyer 34450, Istanbul, Turkey*

[§]*Department of Materials Science and Engineering, University of California, Davis, California 95616, United States*

[‖]*Department of Chemical Engineering, University of California, Davis, California 95616, United States*

[#]*SSRL, SLAC National Accelerator Laboratory, Menlo Park, CA 94025 United States*

[⊥]*Koç University Surface Science and Technology Center (KUYTAM), Koç University, Rumelifeneri Yolu, Sariyer, 34450 Istanbul, Turkey;*

Corresponding Authors:

*E-mail: auzun@ku.edu.tr

*Email: bcgates@ucdavis.edu

S1. Extended X-Ray Absorption Fine Structure (EXAFS) Results of Uncoated Samples

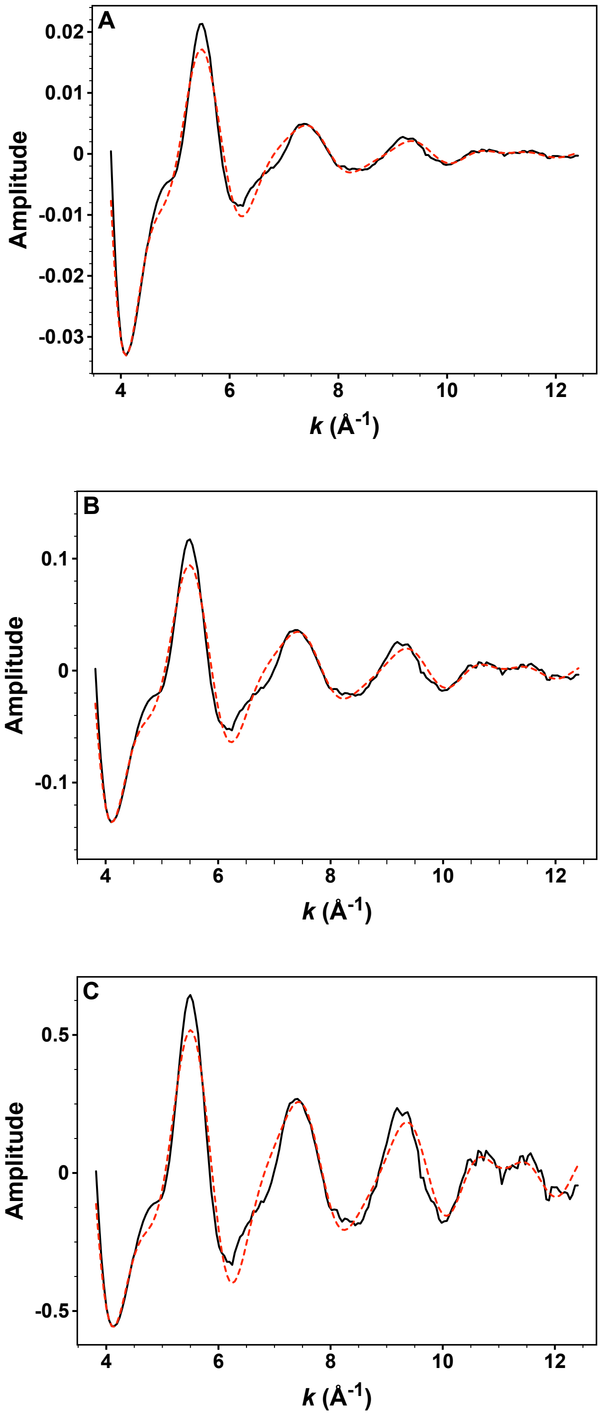

Figure S1. Results of EXAFS analysis characterizing the sample formed by the reaction of $Ir(CO)_2(acac)$ with SiO_2 calcined in flowing O_2 at 793 K. EXAFS function, χ (solid line), and calculated contribution (dotted line) (A, unweighted; B, k^1-weighted; and C, k^2-weighted).

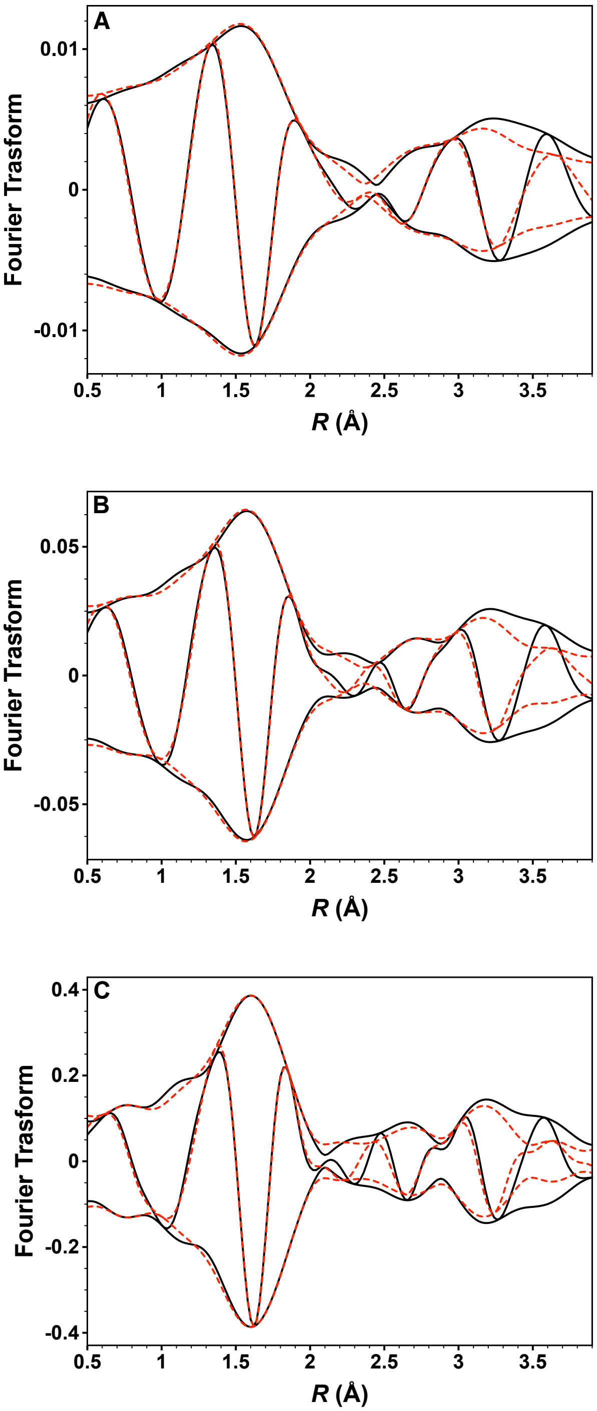

Figure S2. Results of EXAFS analysis characterizing the sample formed by the reaction of $Ir(CO)_2(acac)$ with SiO_2 calcined in flowing O_2 at 793 K. Imaginary part and magnitude of the Fourier transform of data (solid line), and calculated contribution (dotted line) (A, unweighted; B, k^1-weighted; and C, k^2-weighted).

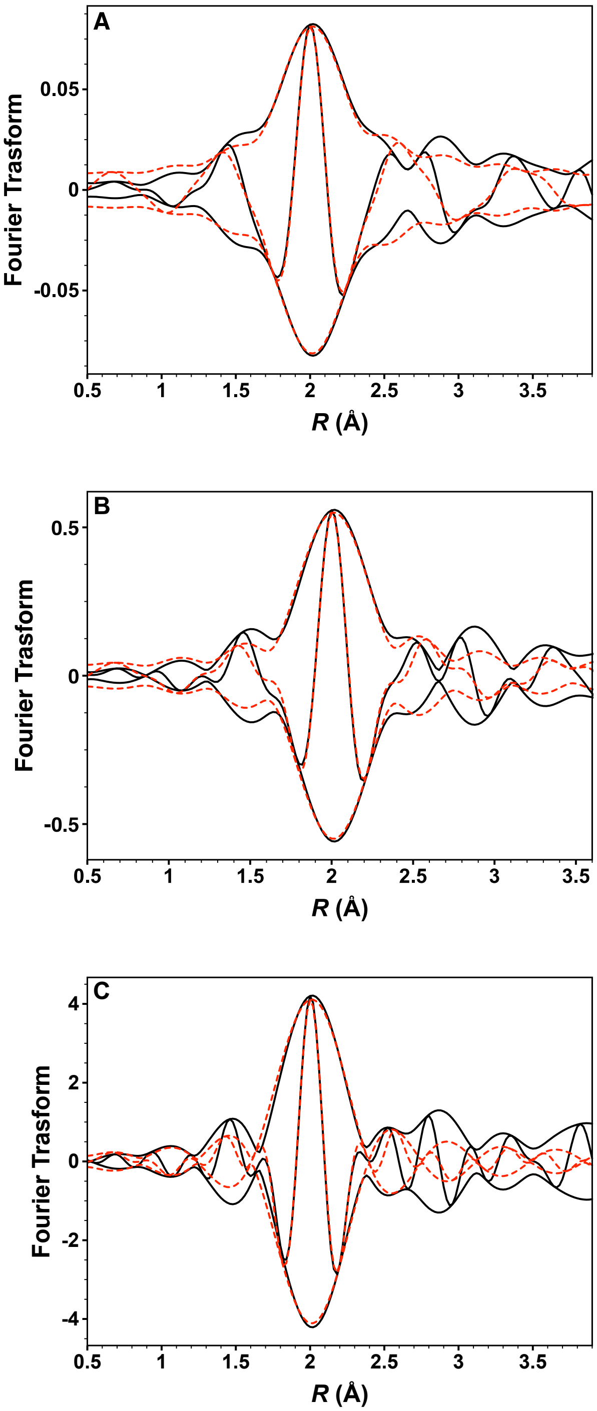

Figure S3. Imaginary part and magnitude of phase- and amplitude-corrected Fourier transform (A, unweighted; B, k^1-weighted; and C, k^2-weighted) of raw data minus calculated Ir–O_s, Ir–O_{CO} and Ir–Si contribution (solid line) and calculated Ir–C_{CO} contribution (dotted line) characterizing the sample formed by the reaction of $Ir(CO)_2(acac)$ with SiO_2 calcined in flowing O_2 at 793 K.

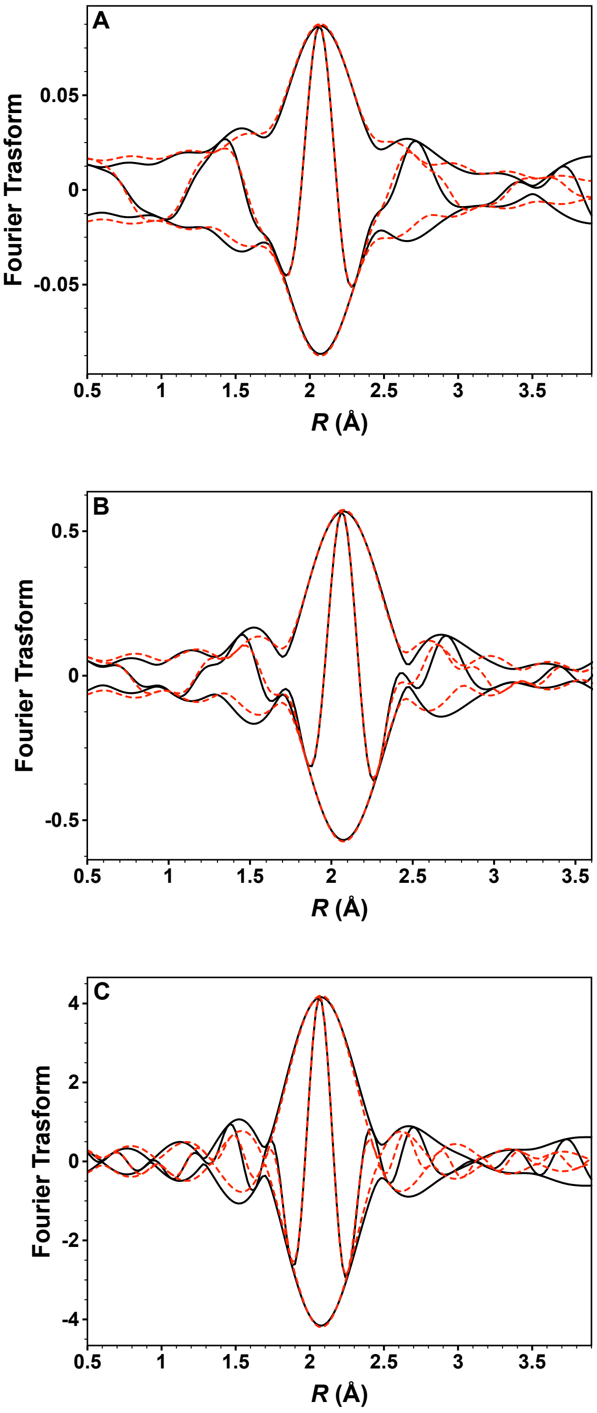

Figure S4. Imaginary part and magnitude of phase- and amplitude-corrected Fourier transform (A, unweighted; B, k^1-weighted; and C, k^2-weighted) of raw data minus calculated Ir−C_{CO}, Ir–O_{CO} and Ir–Si contribution (solid line) and calculated Ir−O_s contribution (dotted line) characterizing the sample formed by the reaction of $Ir(CO)_2(acac)$ with SiO_2 calcined in flowing O_2 at 793 K.

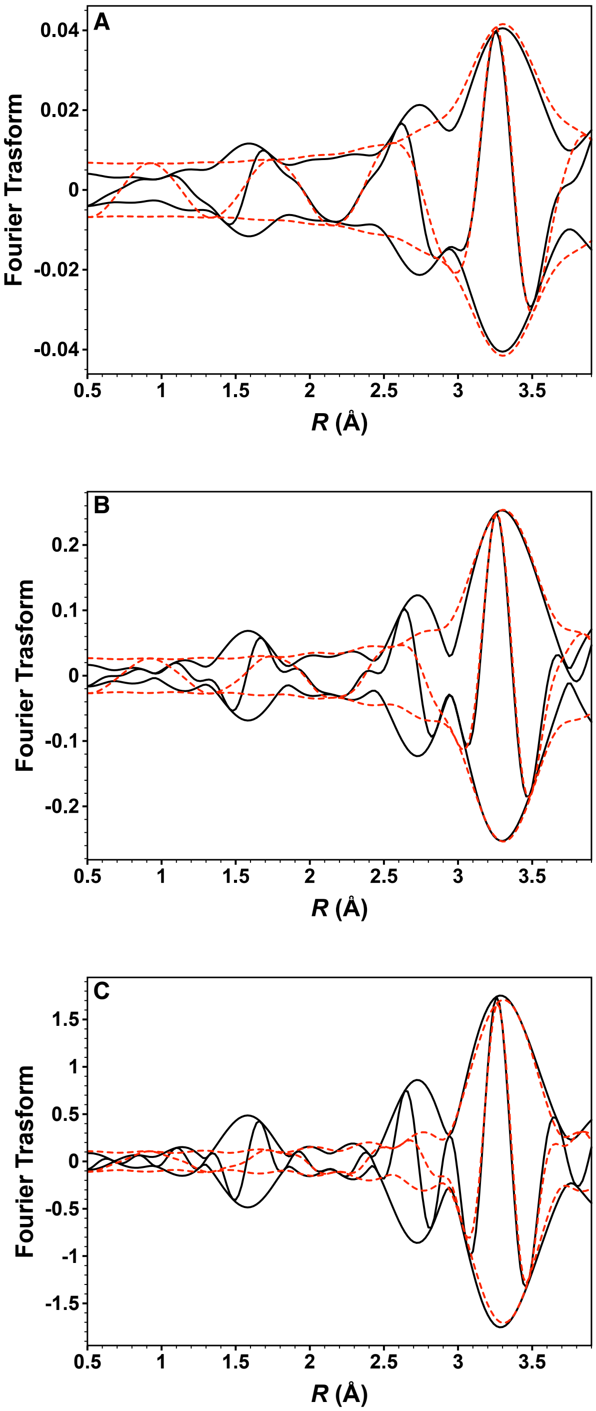

Figure S5. Imaginary part and magnitude of phase- and amplitude-corrected Fourier transform (A, unweighted; B, k^1-weighted; and C, k^2-weighted) of raw data minus calculated Ir−C_{CO}, Ir–O_s and Ir–Si contribution (solid line) and calculated Ir−O_{CO} contribution (dotted line) characterizing the sample formed by the reaction of $Ir(CO)_2(acac)$ with SiO_2 calcined in flowing O_2 at 793 K.

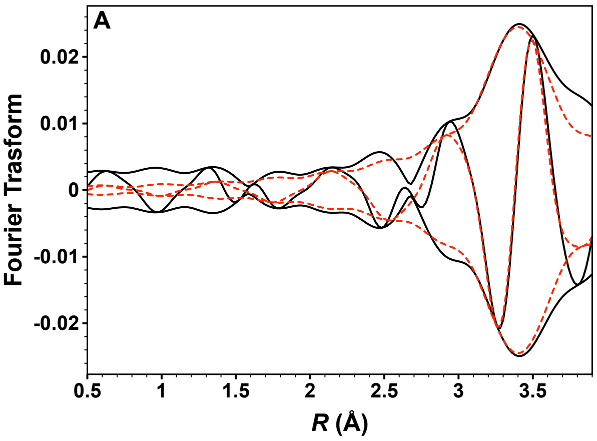

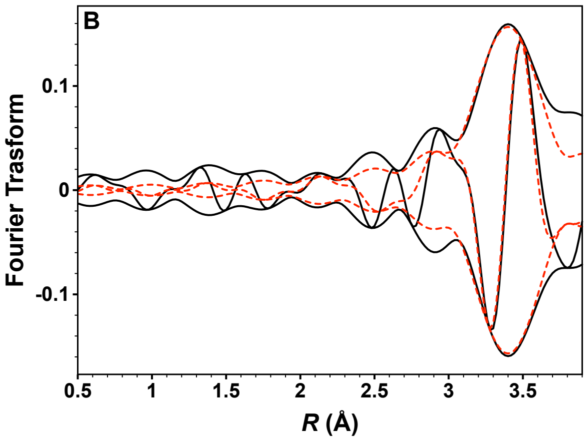

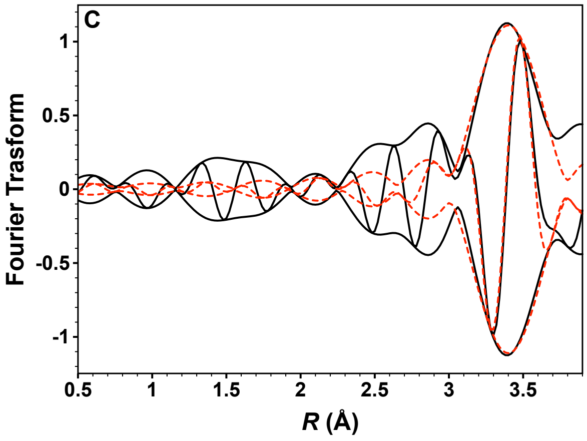

Figure S6. Imaginary part and magnitude of phase- and amplitude-corrected Fourier transform (A, unweighted; B, k^1-weighted; and C, k^2-weighted) of raw data minus calculated Ir−C_{CO}, Ir–O_s and Ir–O_{CO} contribution (solid line) and calculated Ir−Si contribution (dotted line) characterizing the sample formed by the reaction of $Ir(CO)_2(acac)$ with SiO_2 calcined in flowing O_2 at 793 K.

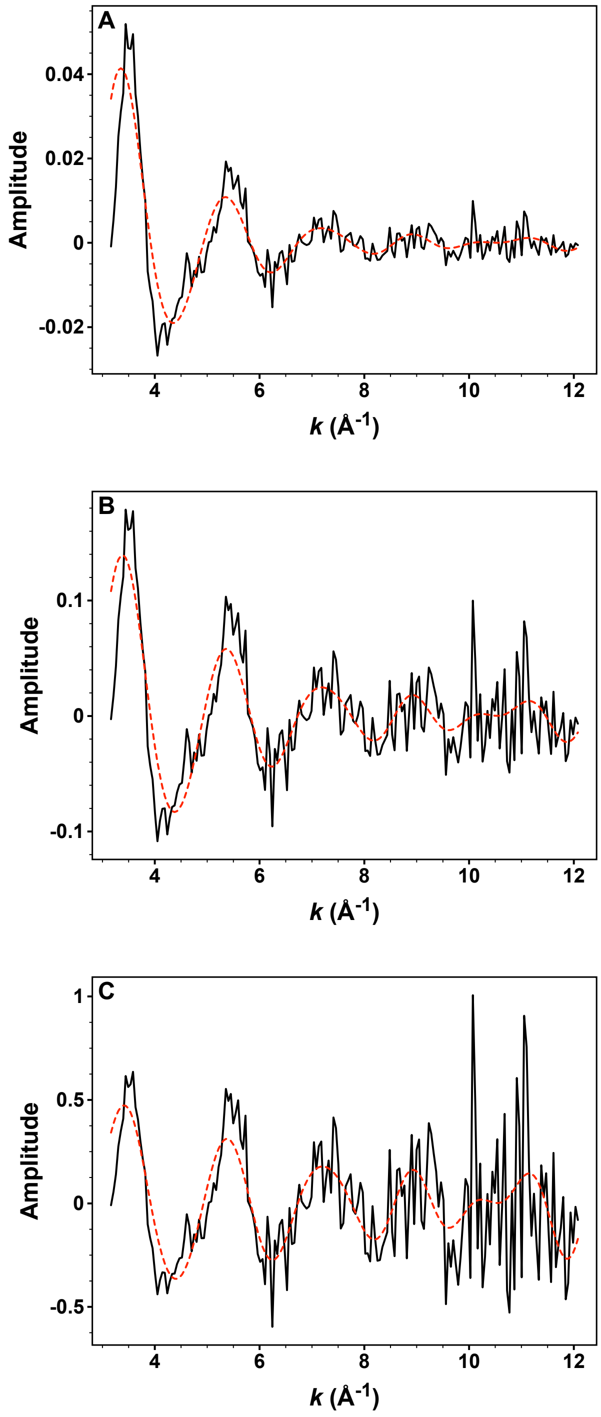

Figure S7. Results of EXAFS analysis characterizing the sample formed by the reaction of $Ir(CO)_2(acac)$ with TiO_2 calcined in flowing O_2 at 673 K. EXAFS function, χ (solid line), and calculated contribution (dotted line) (A, unweighted; B, k^1-weighted; and C, k^2-weighted).

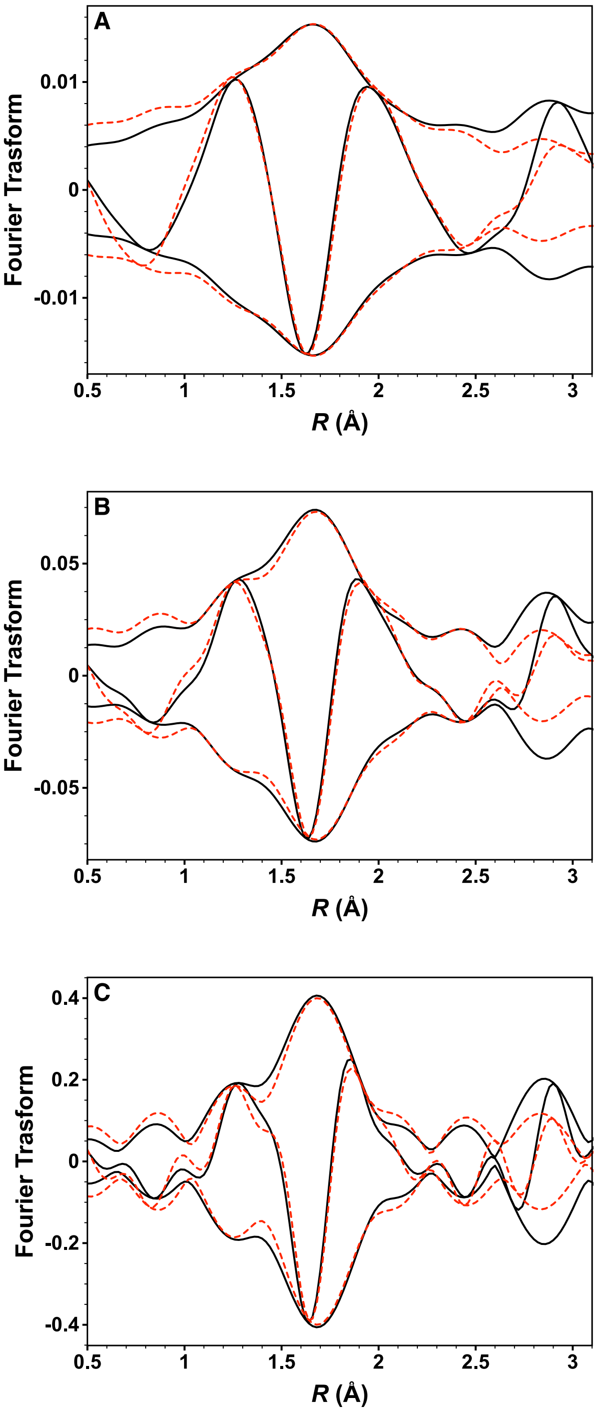

Figure S8. Results of EXAFS analysis characterizing the sample formed by the reaction of $Ir(CO)_2(acac)$ with TiO_2 calcined in flowing O_2 at 673 K. Imaginary part and magnitude of the Fourier transform of data (solid line), and calculated contribution (dotted line) (A, unweighted; B, k^1-weighted; and C, k^2-weighted).

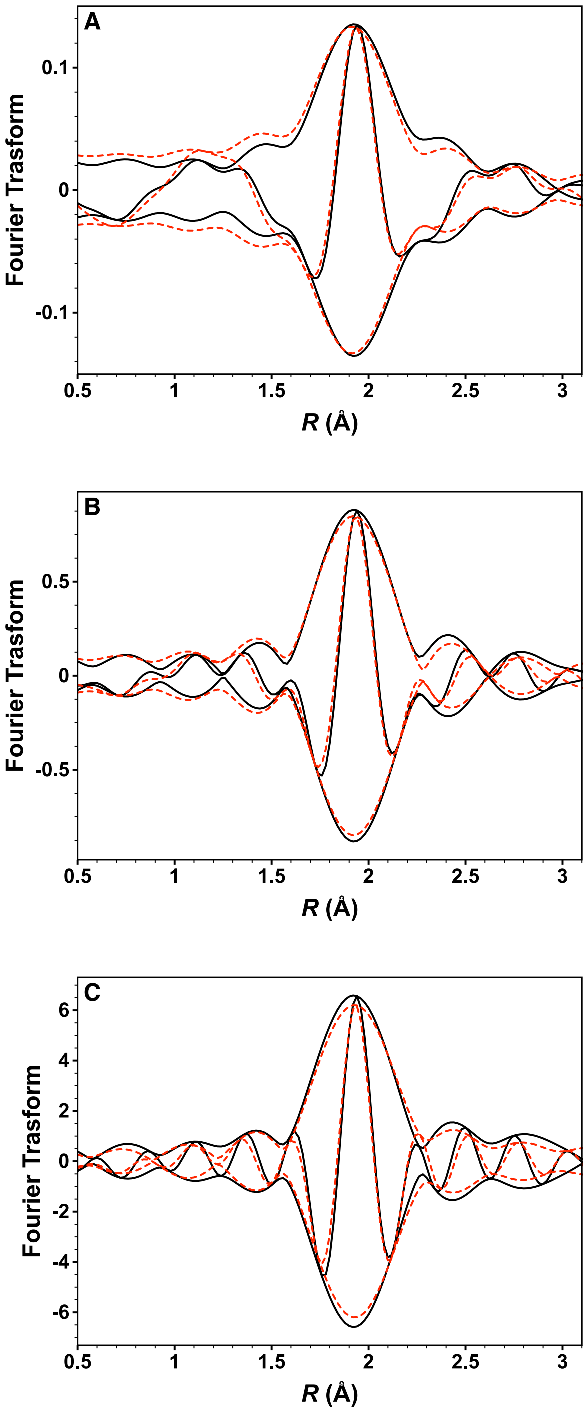

Figure S9. Imaginary part and magnitude of phase- and amplitude-corrected Fourier transform (A, unweighted; B, k^1-weighted; and C, k^2-weighted) of raw data minus calculated Ir−O_s, Ir–O_{CO} and Ir–Ti contribution (solid line) and calculated Ir−C_{CO} contribution (dotted line) characterizing the sample formed by the reaction of $Ir(CO)_2(acac)$ with TiO_2 calcined in flowing O_2 at 673 K.

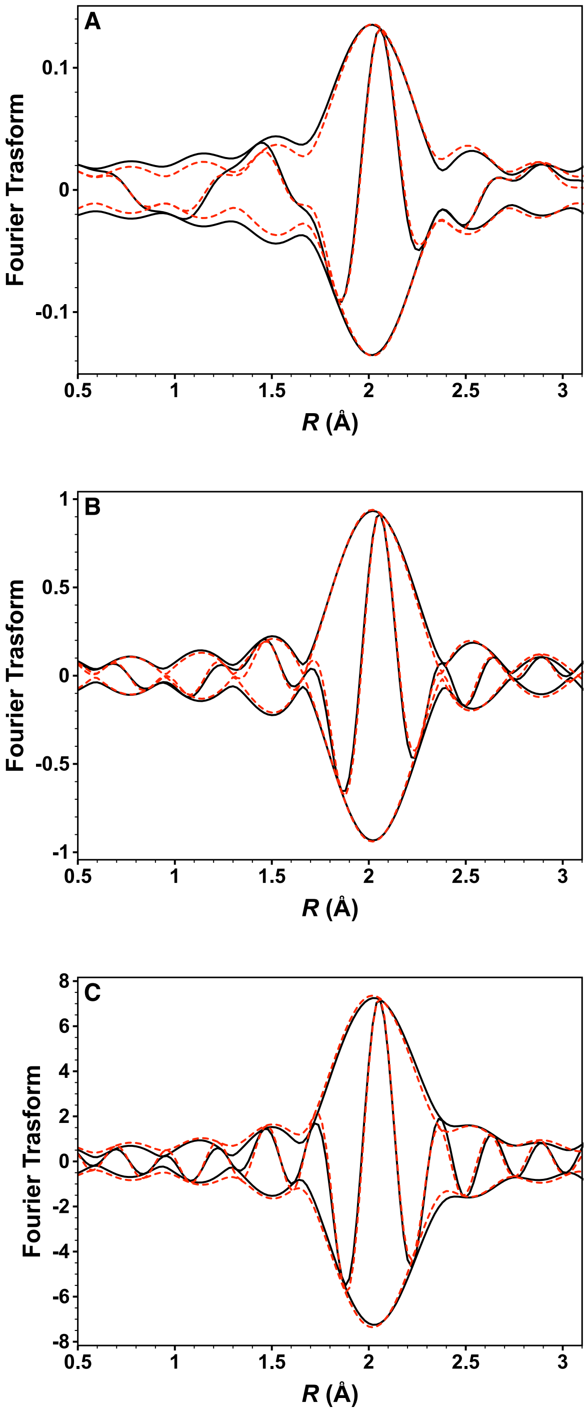

Figure S10. Imaginary part and magnitude of phase- and amplitude-corrected Fourier transform (A, unweighted; B, k^1-weighted; and C, k^2-weighted) of raw data minus calculated Ir–C_{CO}, Ir–O_{CO} and Ir–Ti contribution (solid line) and calculated Ir–O_s contribution (dotted line) characterizing the sample formed by the reaction of $Ir(CO)_2(acac)$ with TiO_2 calcined in flowing O_2 at 673 K.

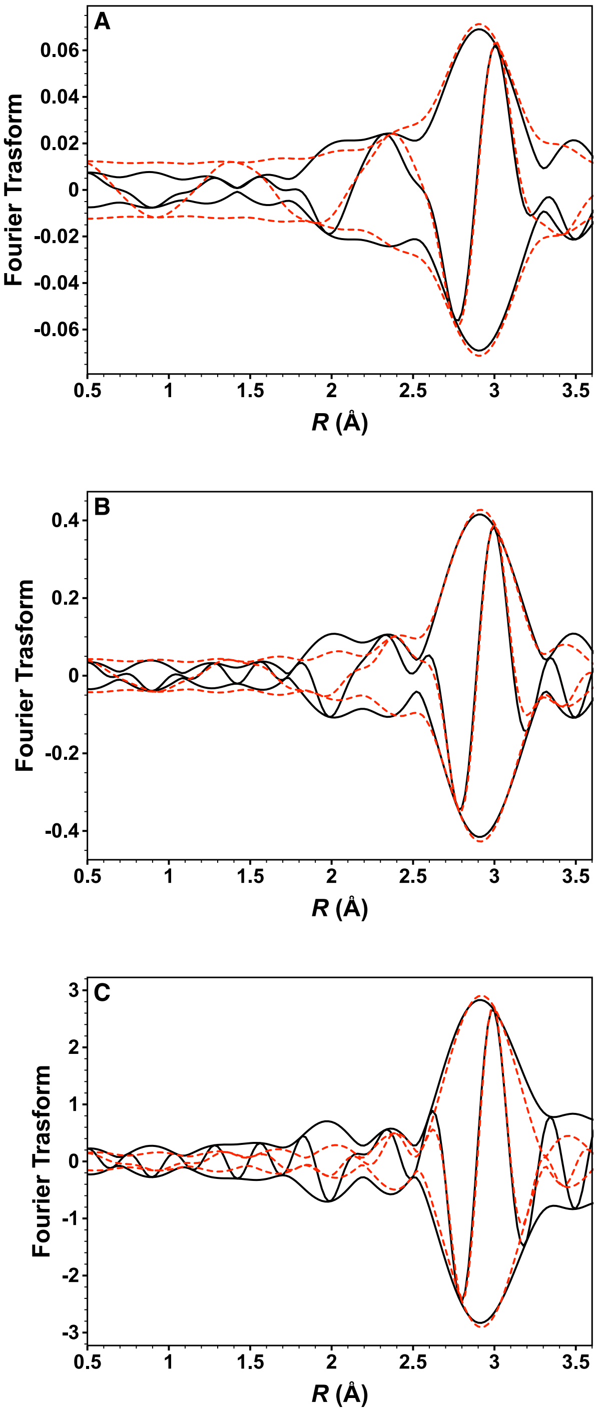

Figure S11. Imaginary part and magnitude of phase- and amplitude-corrected Fourier transform (A, unweighted; B, k^1-weighted; and C, k^2-weighted) of raw data minus calculated Ir–O_s, Ir–O_{CO} and Ir–Ti contribution (solid line) and calculated Ir–O_{CO} contribution (dotted line) characterizing the sample formed by the reaction of $Ir(CO)_2$(acac) with TiO_2 calcined in flowing O_2 at 673 K.

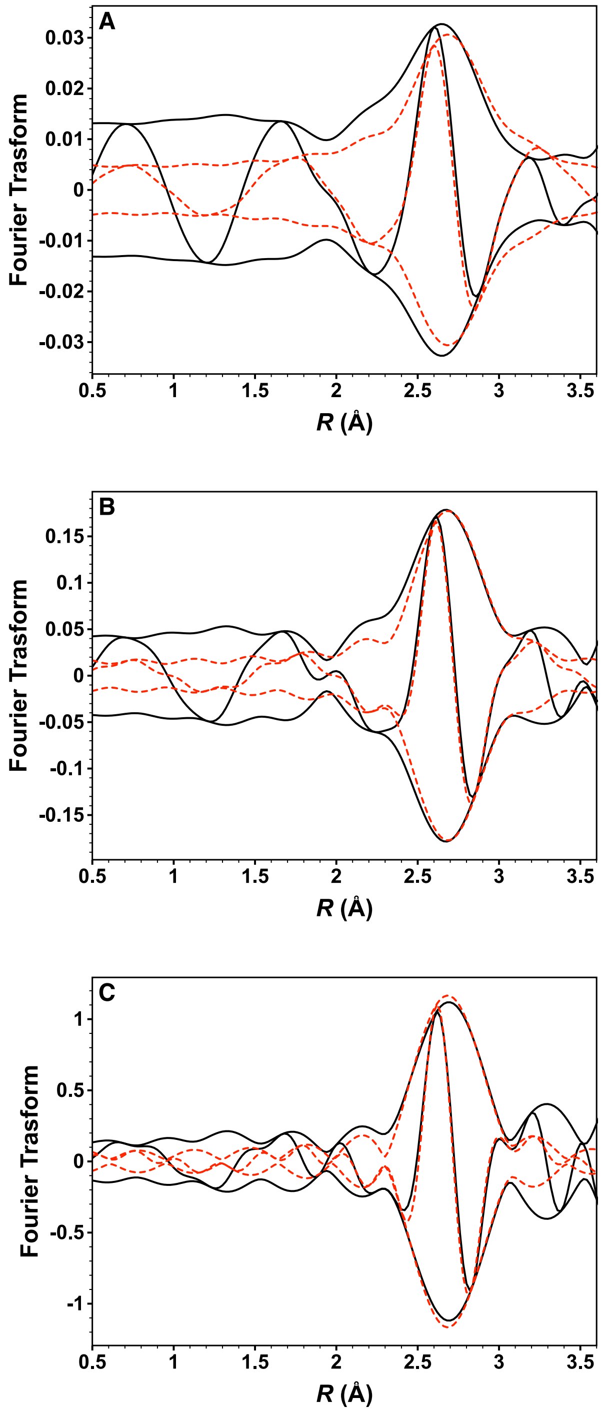

Figure S12. Imaginary part and magnitude of phase- and amplitude-corrected Fourier transform (A, unweighted; B, k^1-weighted; and C, k^2-weighted) of raw data minus calculated Ir–O_s, Ir–C_{CO} and Ir–O_{CO} contribution (solid line) and calculated Ir–Ti contribution (dotted line) characterizing the sample formed by the reaction of $Ir(CO)_2(acac)$ with TiO_2 calcined in flowing O_2 at 673 K.

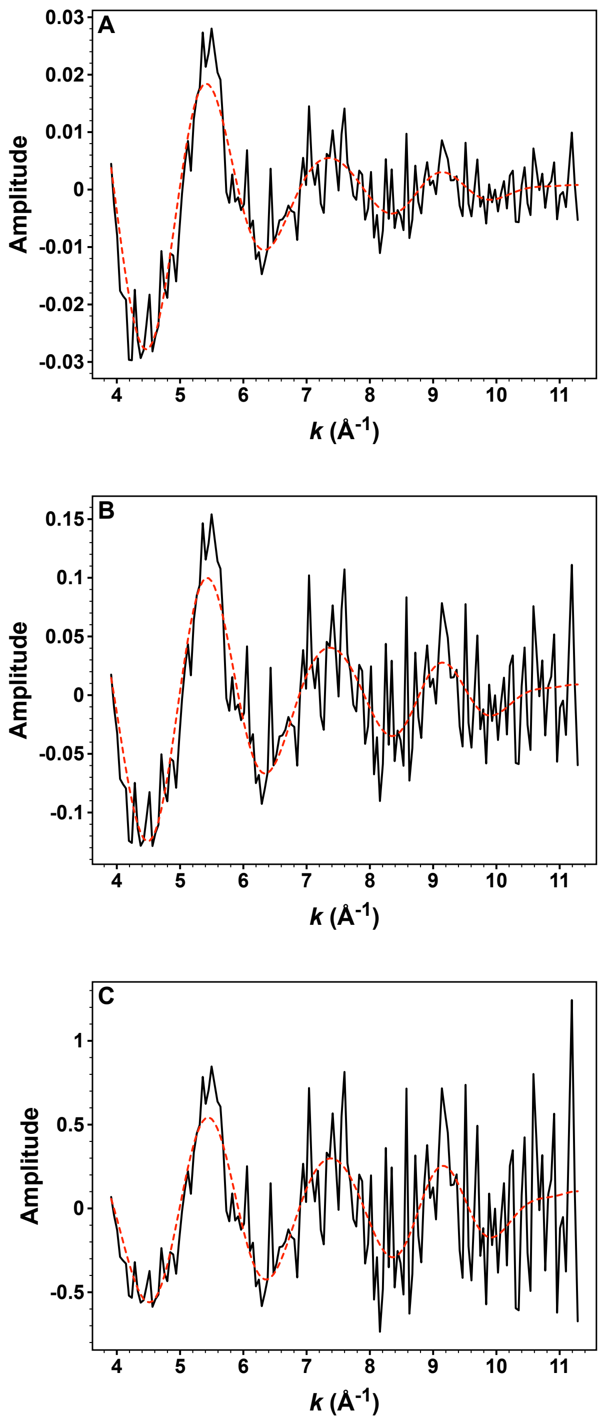

Figure S13. Results of EXAFS analysis characterizing the sample formed by the reaction of $Ir(CO)_2(acac)$ with Fe_2O_3 calcined in flowing O_2 at 773 K. EXAFS function, χ (solid line), and calculated contribution (dotted line) (A, unweighted; B, k^1-weighted; and C, k^2-weighted).

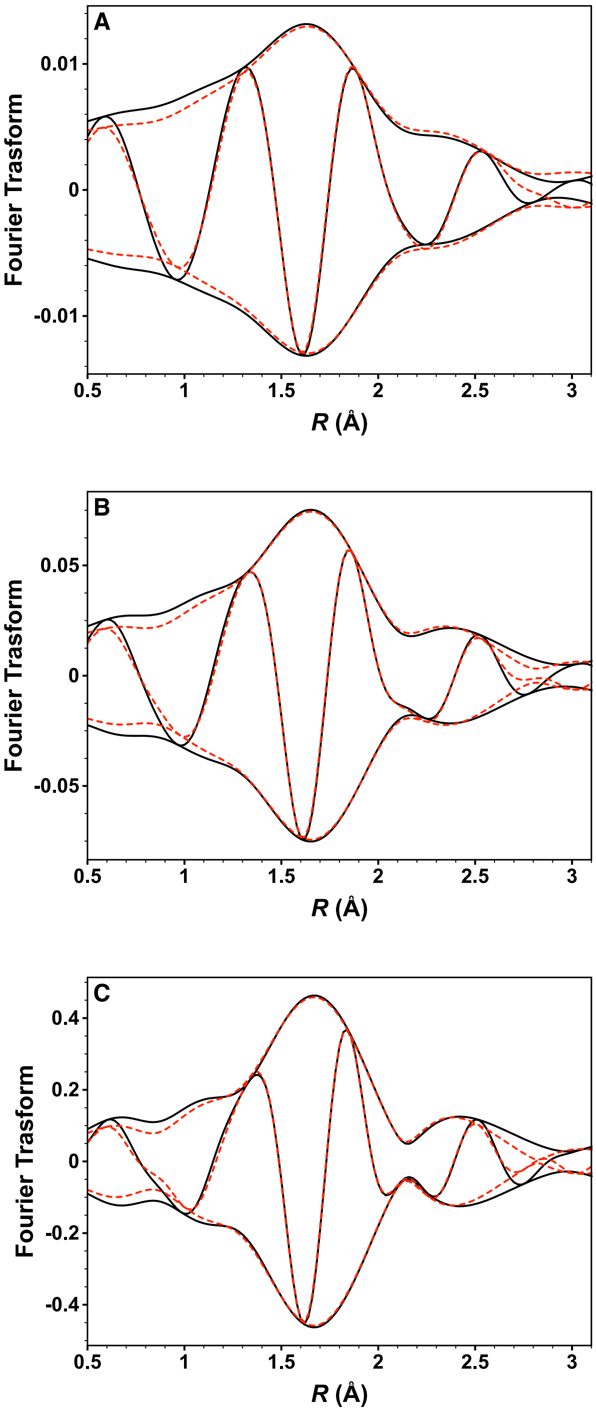

Figure S14. Results of EXAFS analysis characterizing the sample formed by the reaction of $Ir(CO)_2(acac)$ with Fe_2O_3 calcined in flowing O_2 at 773 K. EXAFS function, χ (solid line), and calculated contribution (dotted line) (A, unweighted; B, k^1-weighted; and C, k^2-weighted).

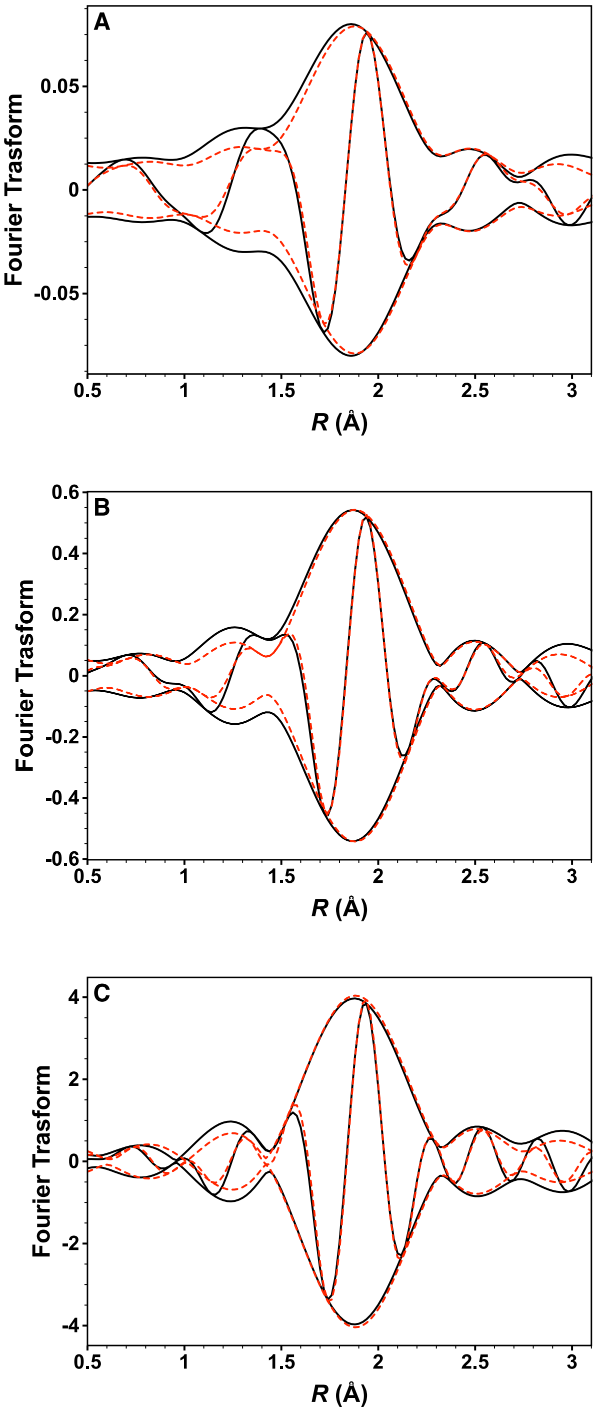

Figure S15. Imaginary part and magnitude of phase- and amplitude-corrected Fourier transform (A, unweighted; B, k^1-weighted; and C, k^2-weighted) of raw data minus calculated Ir−O_s, Ir–O_{CO} and Ir–Fe contribution (solid line) and calculated Ir−C_{CO} contribution (dotted line) characterizing the sample formed by the reaction of $Ir(CO)_2(acac)$ with Fe_2O_3 calcined in flowing O_2 at 773 K.

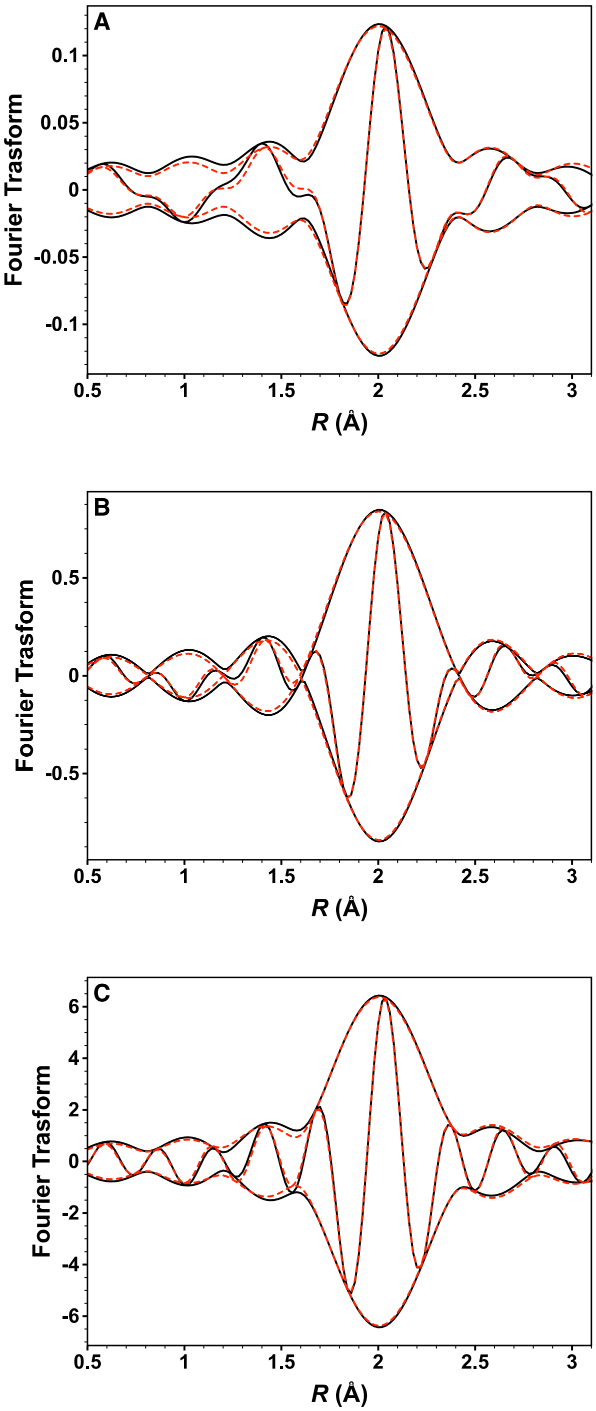

Figure S16. Imaginary part and magnitude of phase- and amplitude-corrected Fourier transform (A, unweighted; B, k^1-weighted; and C, k^2-weighted) of raw data minus calculated Ir–C_{CO}, Ir–O_{CO} and Ir–Fe contribution (solid line) and calculated Ir–O_s contribution (dotted line) characterizing the sample formed by the reaction of $Ir(CO)_2$(acac) with Fe_2O_3 calcined in flowing O_2 at 773 K.

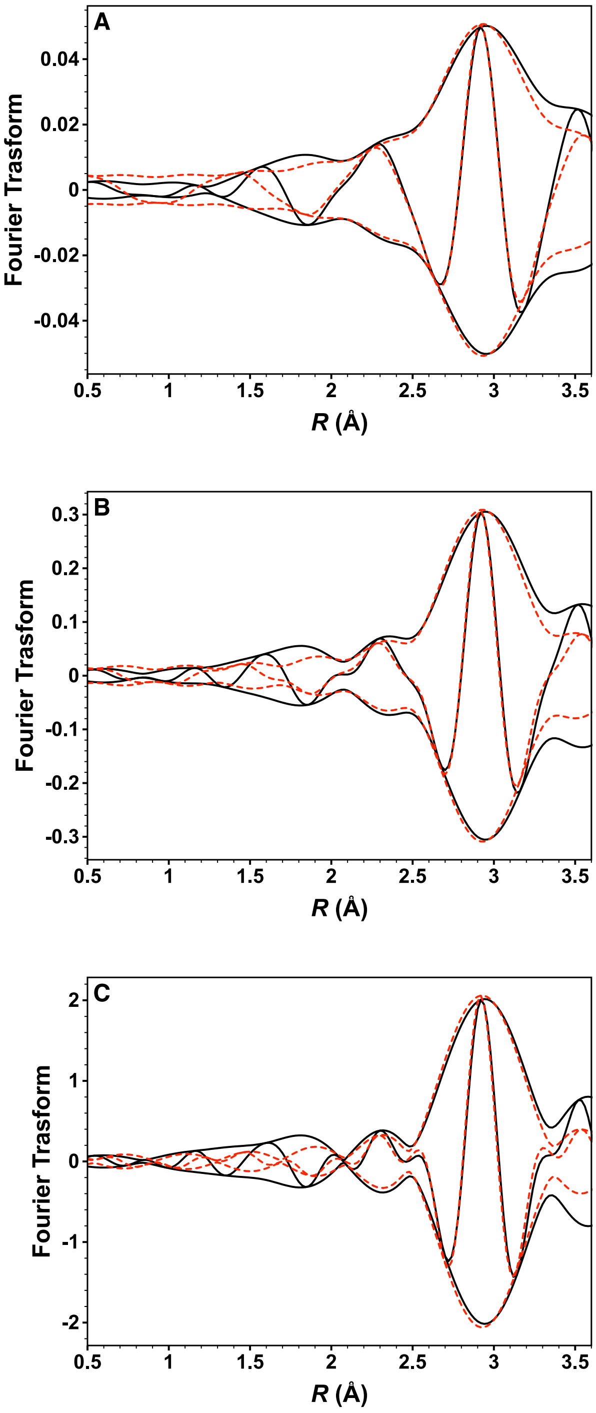

Figure S17. Imaginary part and magnitude of phase- and amplitude-corrected Fourier transform (A, unweighted; B, k^1-weighted; and C, k^2-weighted) of raw data minus calculated Ir−C_{CO}, Ir–O_s and Ir–Fe contribution (solid line) and calculated Ir−O_{CO} contribution (dotted line) characterizing the sample formed by the reaction of $Ir(CO)_2(acac)$ with Fe_2O_3 calcined in flowing O_2 at 773 K.

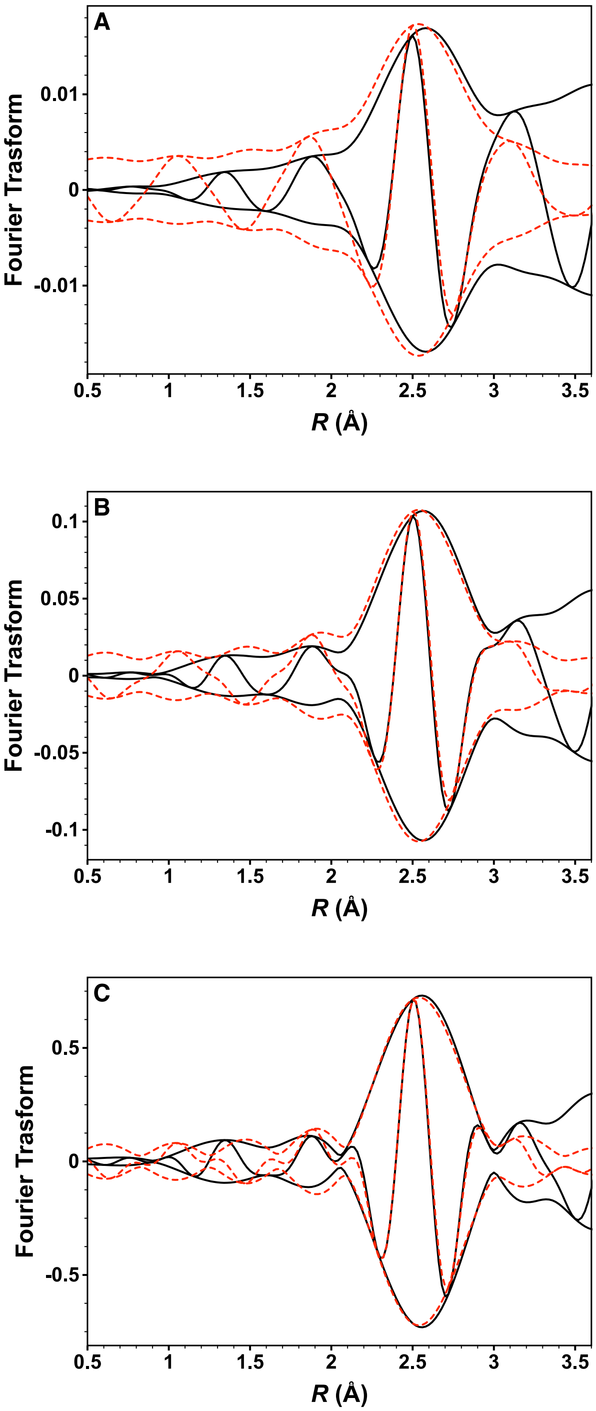

Figure S18. Imaginary part and magnitude of phase- and amplitude-corrected Fourier transform (A, unweighted; B, k^1-weighted; and C, k^2-weighted) of raw data minus calculated Ir−C_{CO}, Ir–O_s and Ir–O_{CO} contribution (solid line) and calculated Ir−Fe contribution (dotted line) characterizing the sample formed by the reaction of $Ir(CO)_2(acac)$ with Fe_2O_3 calcined in flowing O_2 at 773 K.

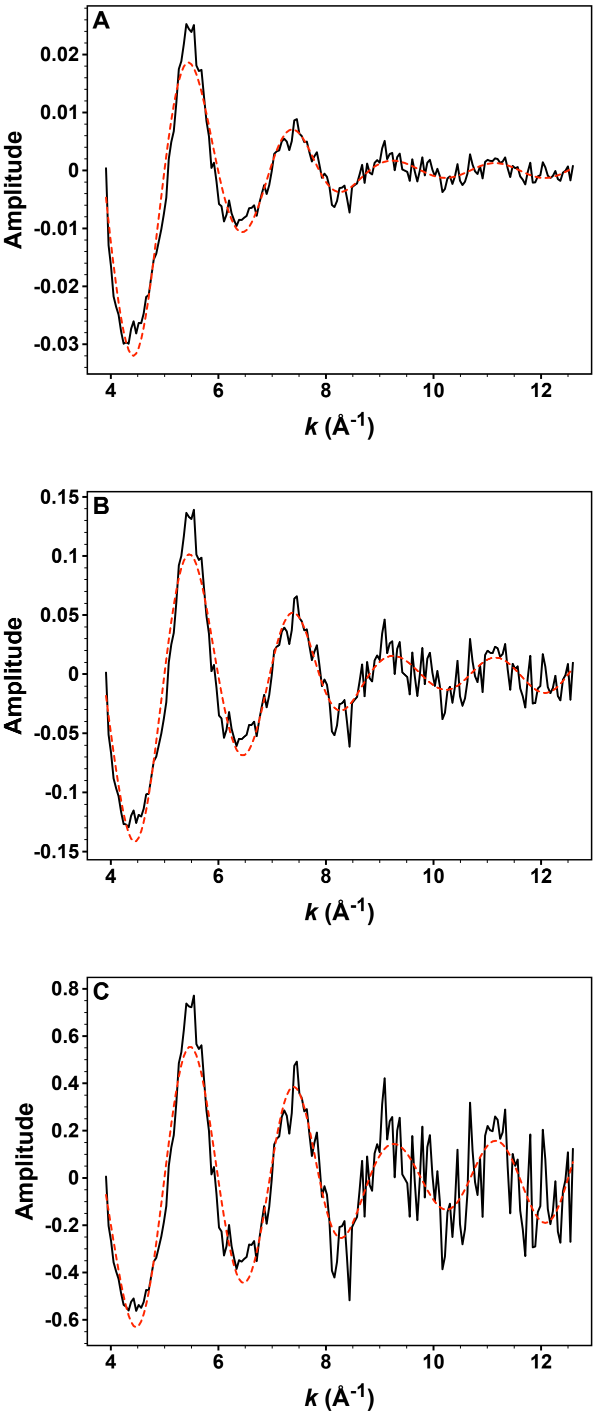

Figure S19. Results of EXAFS analysis characterizing the sample formed by the reaction of $Ir(CO)_2(acac)$ with CeO_2 calcined in flowing O_2 at 673 K. EXAFS function, χ (solid line), and calculated contribution (dotted line) (A, unweighted; B, k^1-weighted; and C, k^2-weighted).

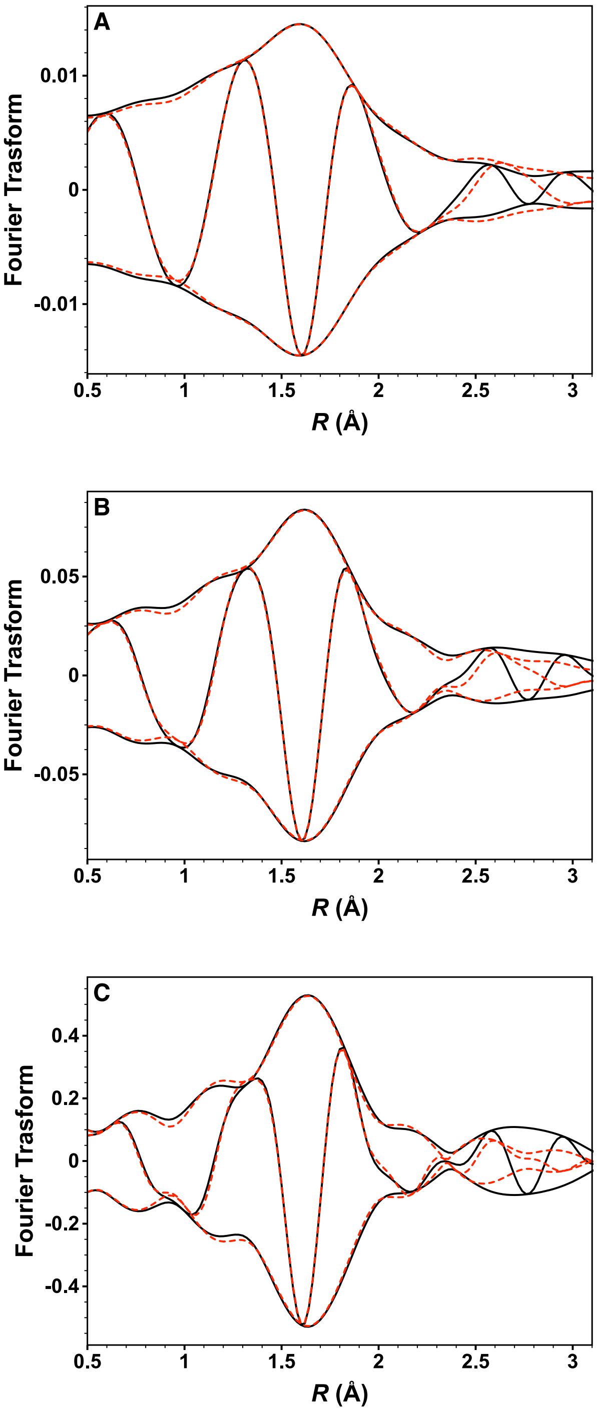

Figure S20. Results of EXAFS analysis characterizing the sample formed by the reaction of $Ir(CO)_2(acac)$ with CeO_2 calcined in flowing O_2 at 673 K. Imaginary part and magnitude of the Fourier transform of data (solid line), and calculated contribution (dotted line) (A, unweighted; B, k^1-weighted; and C, k^2-weighted).

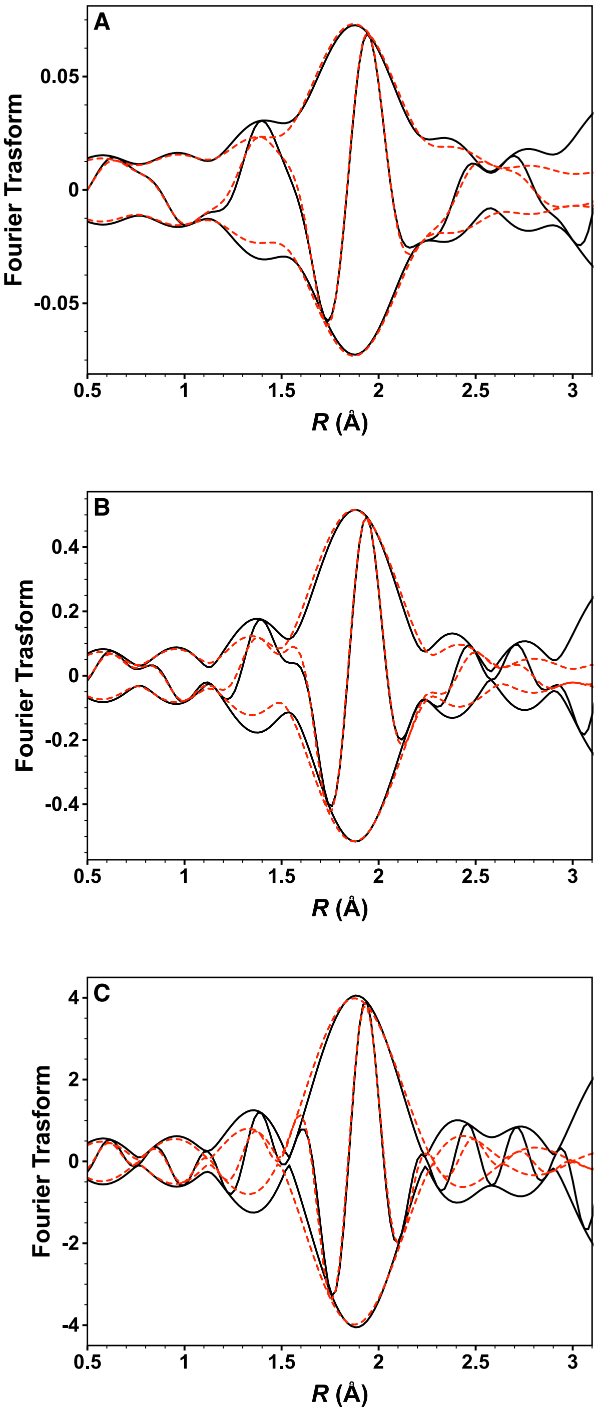

Figure S21. Imaginary part and magnitude of phase- and amplitude-corrected Fourier transform (A, unweighted; B, k^1-weighted; and C, k^2-weighted) of raw data minus calculated Ir−O_s, Ir–O_{CO} and Ir–Ce contribution (solid line) and calculated Ir−C_{CO} contribution (dotted line) characterizing the sample formed by the reaction of $Ir(CO)_2(acac)$ with CeO_2 calcined in flowing O_2 at 673 K.

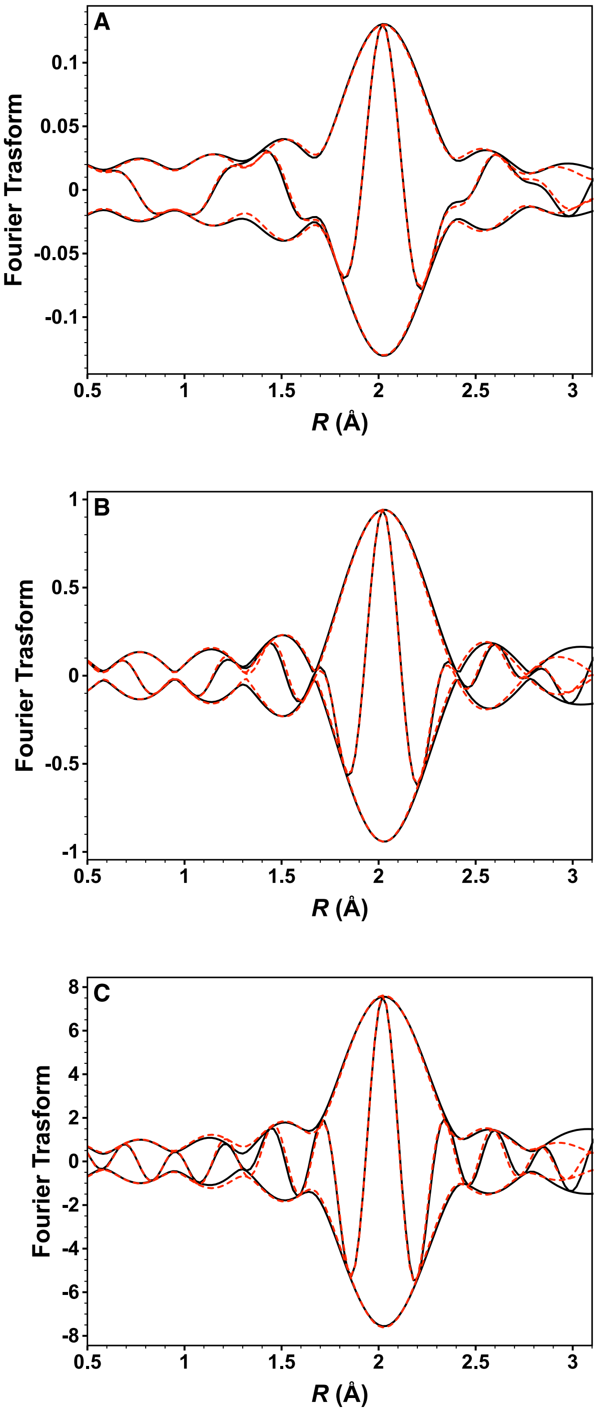

Figure S22. Imaginary part and magnitude of phase- and amplitude-corrected Fourier transform (A, unweighted; B, k^1-weighted; and C, k^2-weighted) of raw data minus calculated Ir–C_{CO}, Ir–O_{CO} and Ir–Ce contribution (solid line) and calculated Ir−O_s contribution (dotted line) characterizing the sample formed by the reaction of $Ir(CO)_2(acac)$ with CeO_2 calcined in flowing O_2 at 673 K.

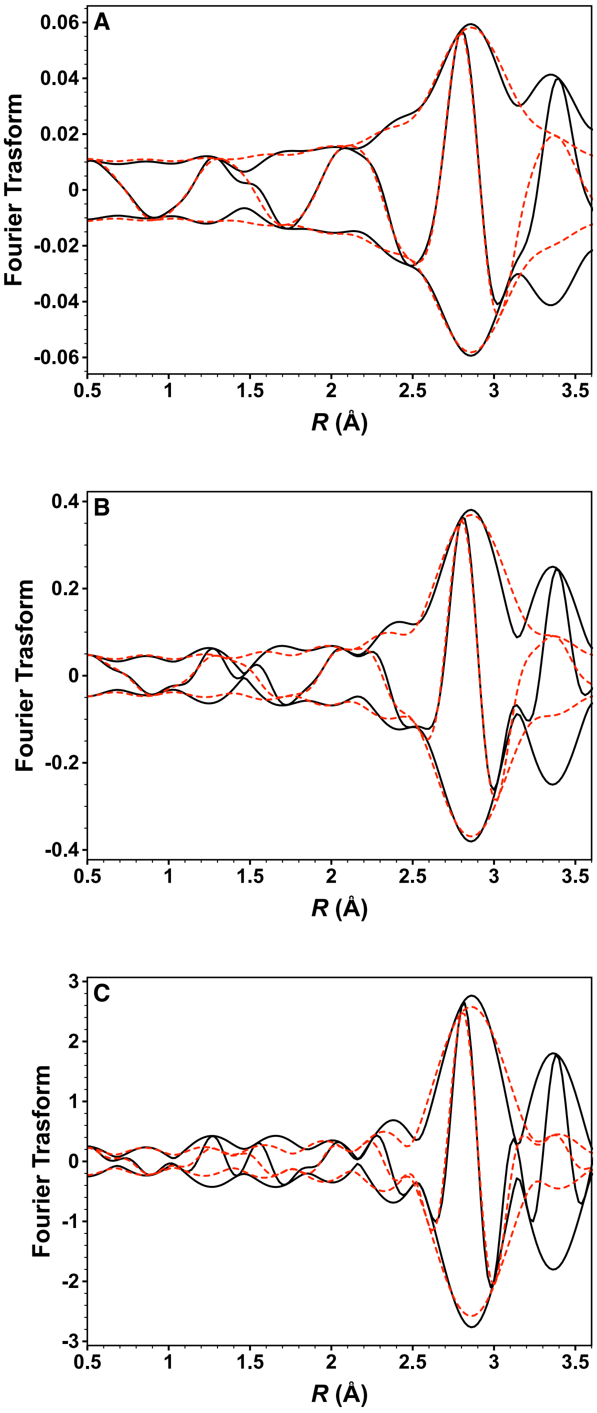

Figure S23. Imaginary part and magnitude of phase- and amplitude-corrected Fourier transform (A, unweighted; B, k^1-weighted; and C, k^2-weighted) of raw data minus calculated Ir–C_{CO}, Ir–O_s and Ir–Ce contribution (solid line) and calculated Ir−O_{CO} contribution (dotted line) characterizing the sample formed by the reaction of $Ir(CO)_2(acac)$ with CeO_2 calcined in flowing O_2 at 673 K.

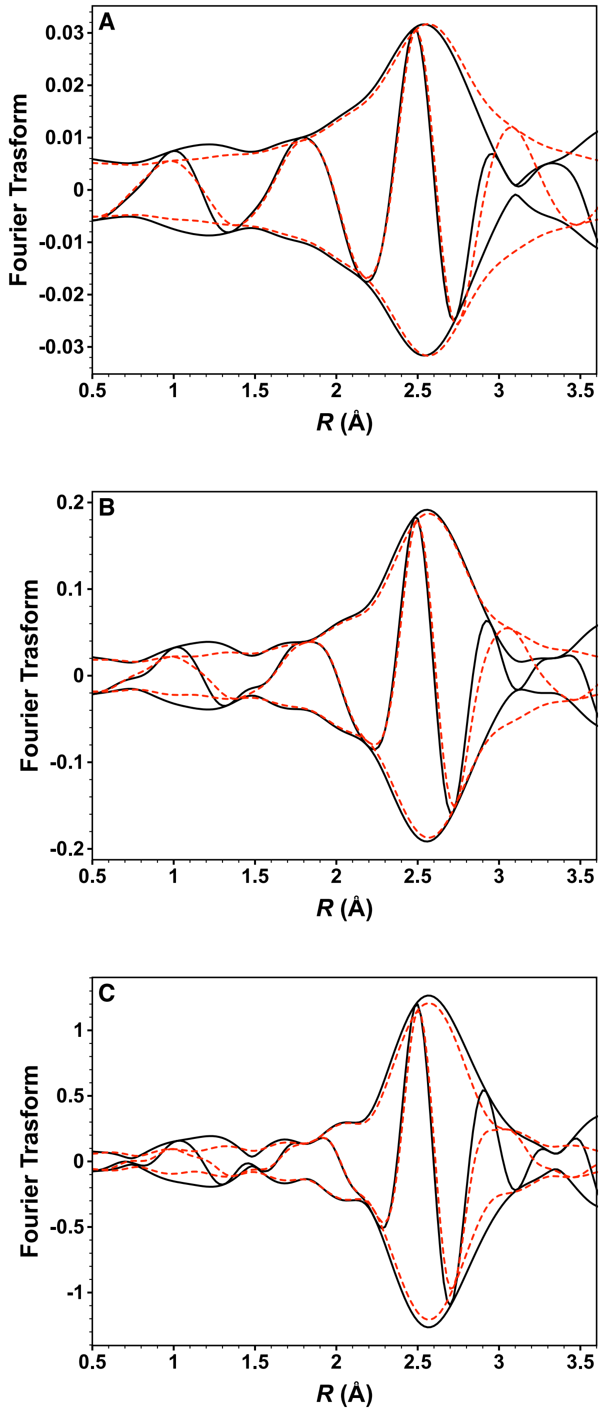

Figure S24. Imaginary part and magnitude of phase- and amplitude-corrected Fourier transform (A, unweighted; B, k^1-weighted; and C, k^2-weighted) of raw data minus calculated Ir–C_{CO}, Ir–O_s and Ir–O_{CO} contribution (solid line) and calculated Ir–Ce contribution (dotted line) characterizing the sample formed by the reaction of $Ir(CO)_2(acac)$ with CeO_2 calcined in flowing O_2 at 673 K.

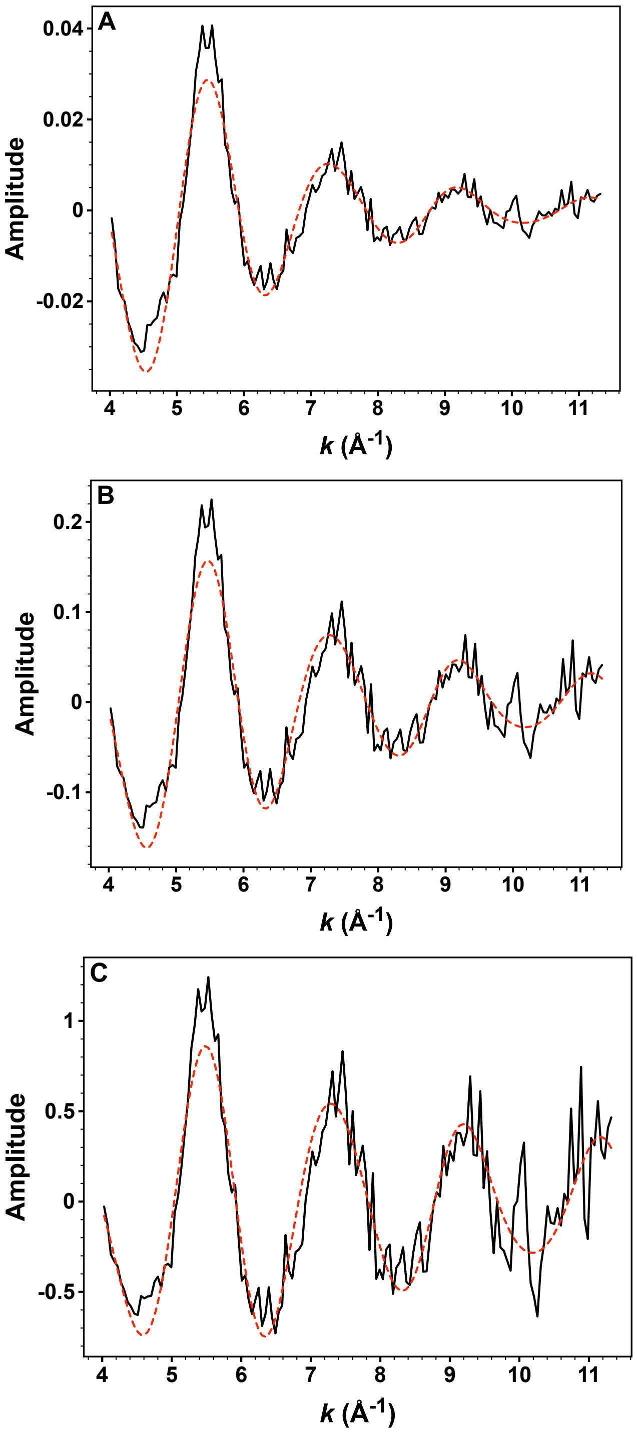

Figure S25. Results of EXAFS analysis characterizing the sample formed by the reaction of $Ir(CO)_2(acac)$ with MgO calcined in flowing O_2 at 973 K. EXAFS function, χ (solid line), and calculated contribution (dotted line) (A, unweighted; B, k^1-weighted; and C, k^2-weighted).

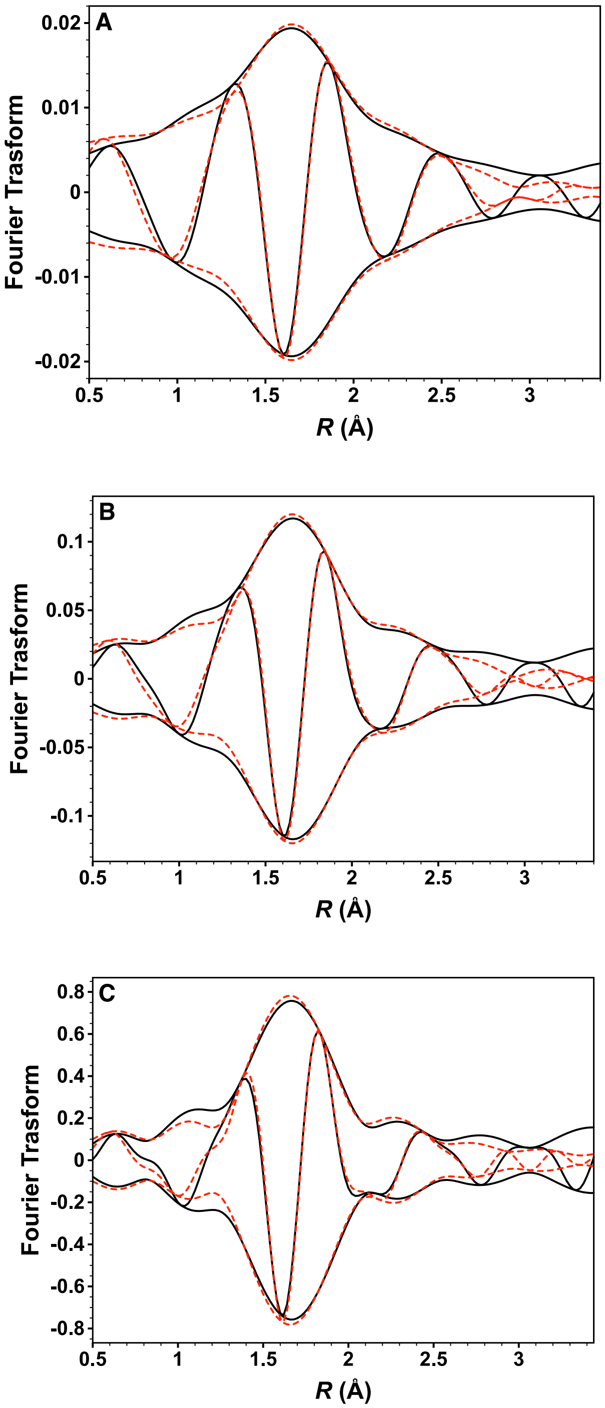

Figure S26. Results of EXAFS analysis characterizing the sample formed by the reaction of $Ir(CO)_2(acac)$ with MgO calcined in flowing O_2 at 973 K. EXAFS function, χ (solid line), and calculated contribution (dotted line) (A, unweighted; B, k^1-weighted; and C, k^2-weighted).

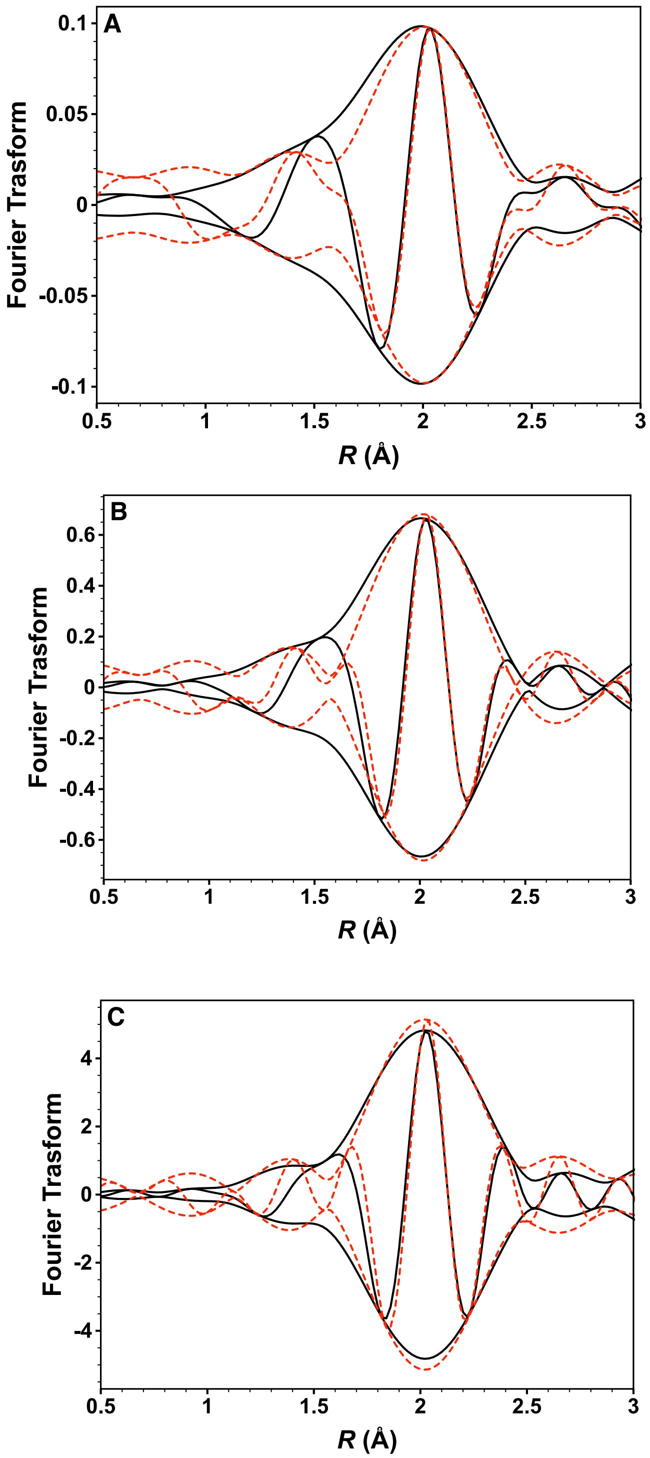

Figure S27. Imaginary part and magnitude of phase- and amplitude-corrected Fourier transform (A, unweighted; B, k^1-weighted; and C, k^2-weighted) of raw data minus calculated Ir−O_s, Ir–O_{CO} and Ir–Mg contribution (solid line) and calculated Ir−C_{CO} contribution (dotted line) characterizing the sample formed by the reaction of $Ir(CO)_2(acac)$ with MgO calcined in flowing O_2 at 973 K.

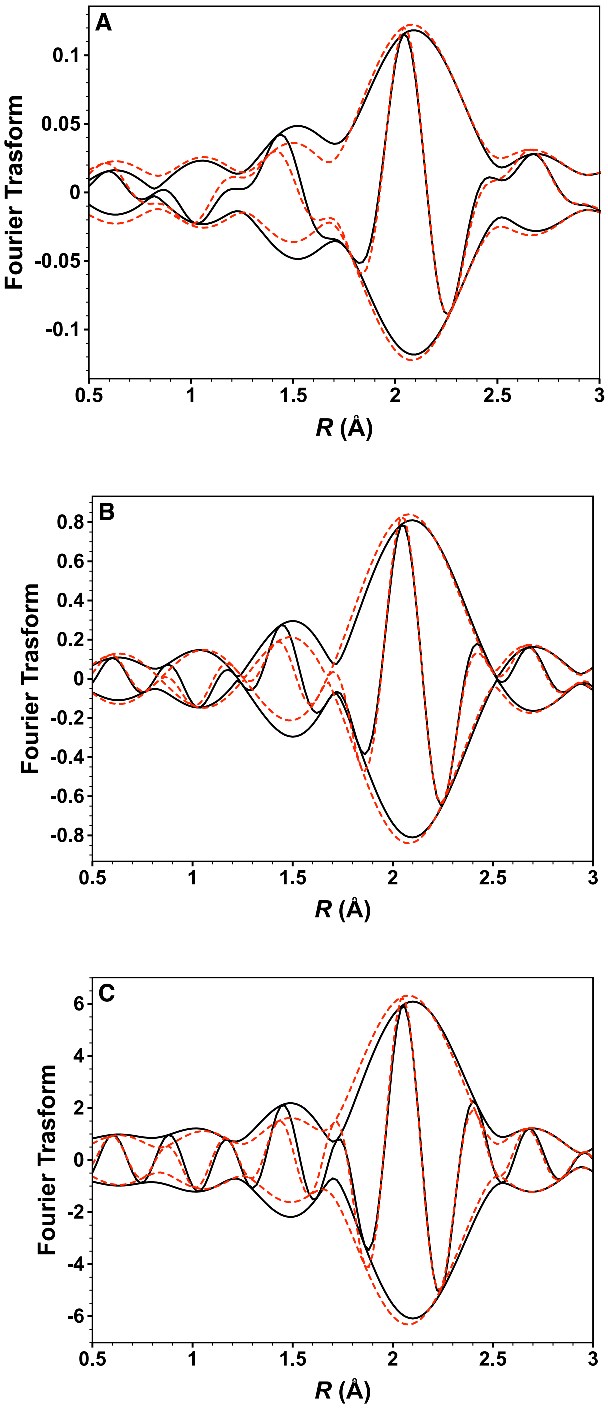

Figure S28. Imaginary part and magnitude of phase- and amplitude-corrected Fourier transform (A, unweighted; B, k^1-weighted; and C, k^2-weighted) of raw data minus calculated Ir−C_{CO}, Ir−O_{CO} and Ir–Mg contribution (solid line) and calculated Ir−O_s contribution (dotted line) characterizing the sample formed by the reaction of $Ir(CO)_2$(acac) with MgO calcined in flowing O_2 at 973 K.

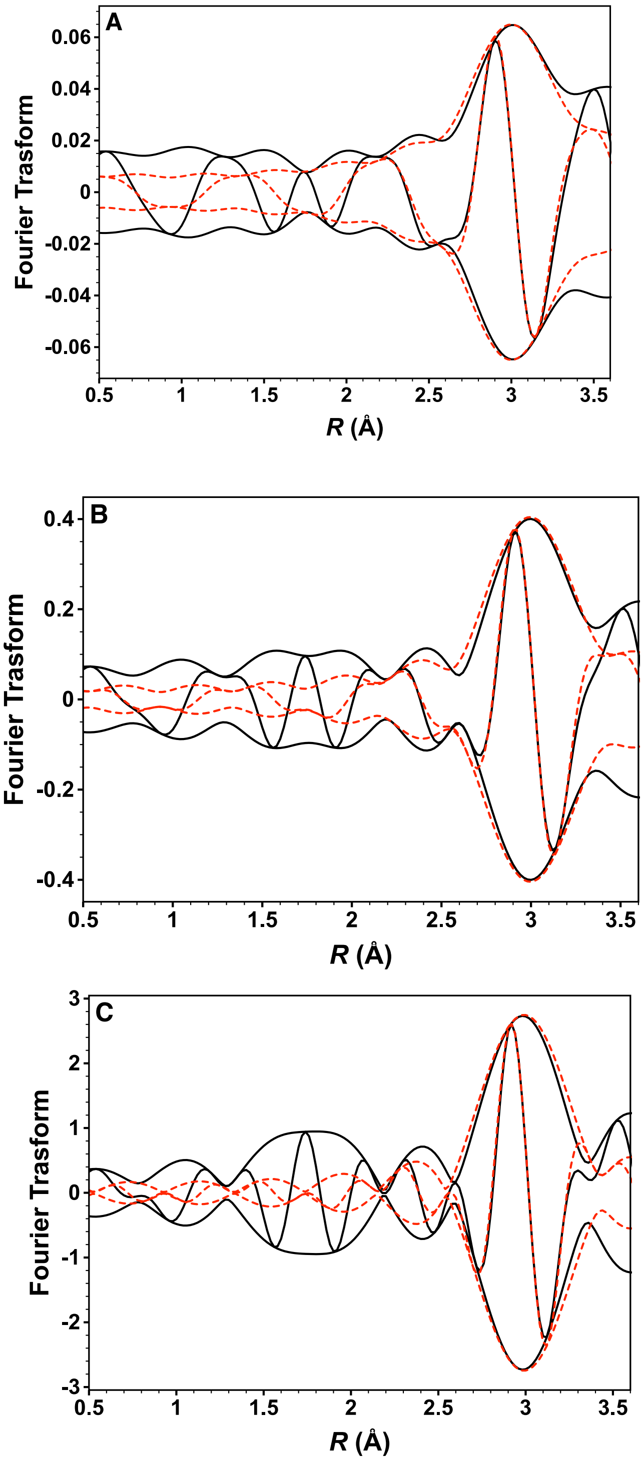

Figure S29. Imaginary part and magnitude of phase- and amplitude-corrected Fourier transform (A, unweighted; B, k^1-weighted; and C, k^2-weighted) of raw data minus calculated Ir−C_{CO}, Ir–O_s and Ir–Mg contribution (solid line) and calculated Ir−O_{CO} contribution (dotted line) characterizing the sample formed by the reaction of $Ir(CO)_2(acac)$ with MgO calcined in flowing O_2 at 973 K.

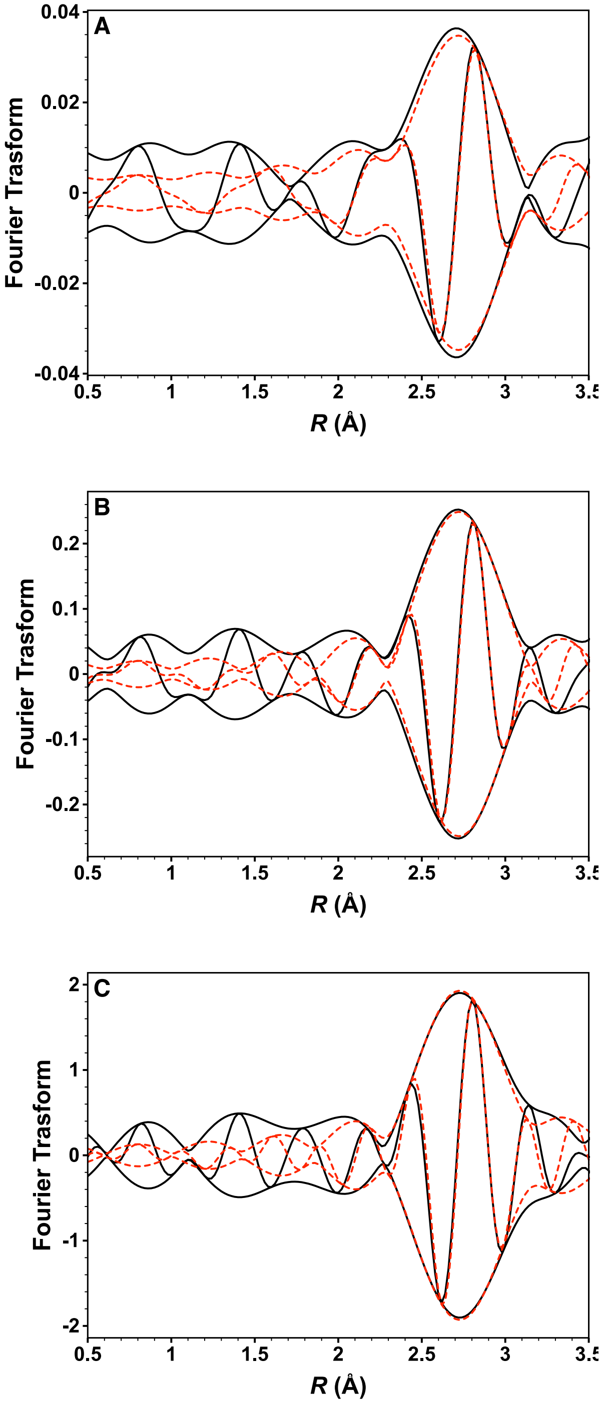

Figure S30. Imaginary part and magnitude of phase- and amplitude-corrected Fourier transform (A, unweighted; B, k^1-weighted; and C, k^2-weighted) of raw data minus calculated Ir−C_{CO}, Ir–O_s and Ir–O_{CO} contribution (solid line) and calculated Ir−Mg contribution (dotted line) characterizing the sample formed by the reaction of $Ir(CO)_2(acac)$ with MgO calcined in flowing O_2 at 973 K.

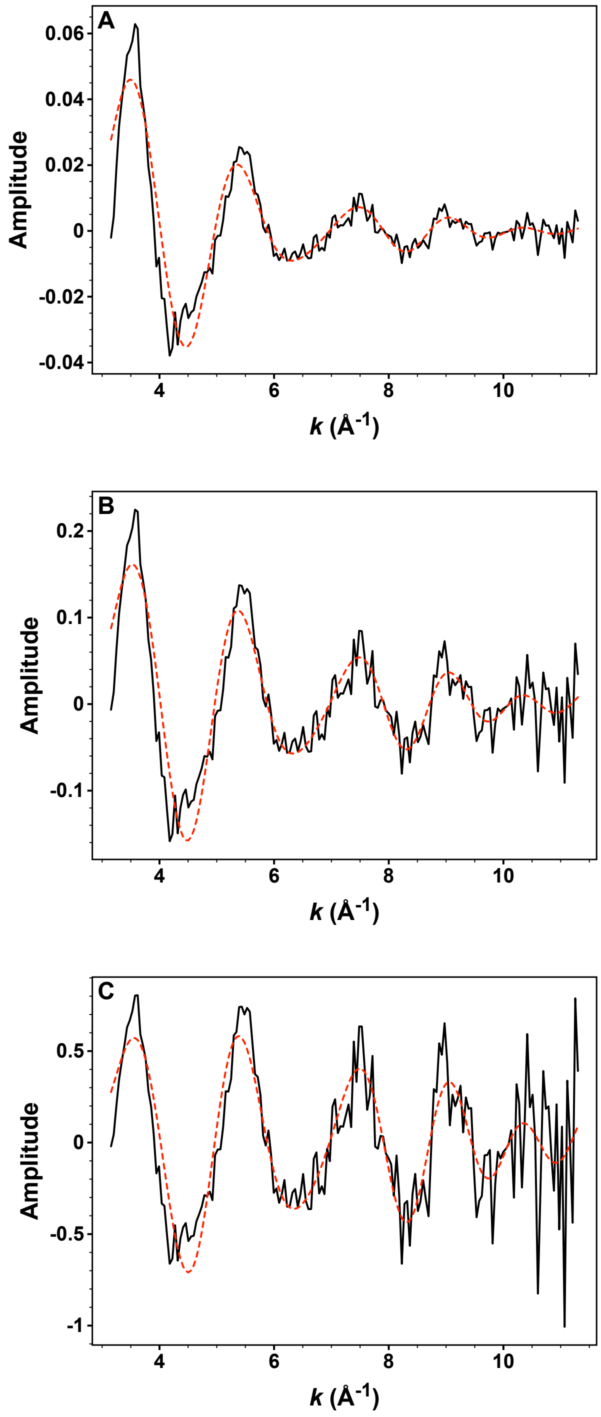

Figure S31. Results of EXAFS analysis characterizing the sample formed by the reaction of $Ir(CO)_2(acac)$ with La_2O_3 calcined in flowing O_2 at 873 K. EXAFS function, χ (solid line), and calculated contribution (dotted line) (A, unweighted; B, k^1-weighted; and C, k^2-weighted).

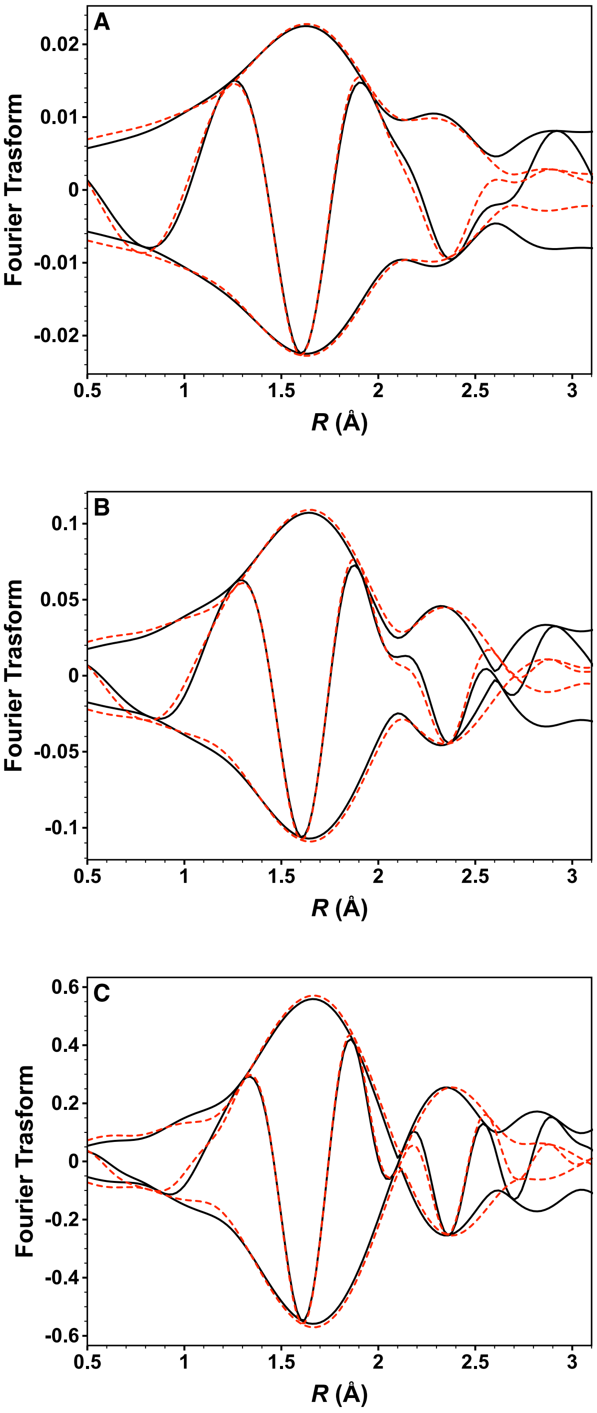

Figure S32. Results of EXAFS analysis characterizing the sample formed by the reaction of $Ir(CO)_2(acac)$ with La_2O_3 calcined in flowing O_2 at 873 K. EXAFS function, χ (solid line), and calculated contribution (dotted line) (A, unweighted; B, k^1-weighted; and C, k^2-weighted).

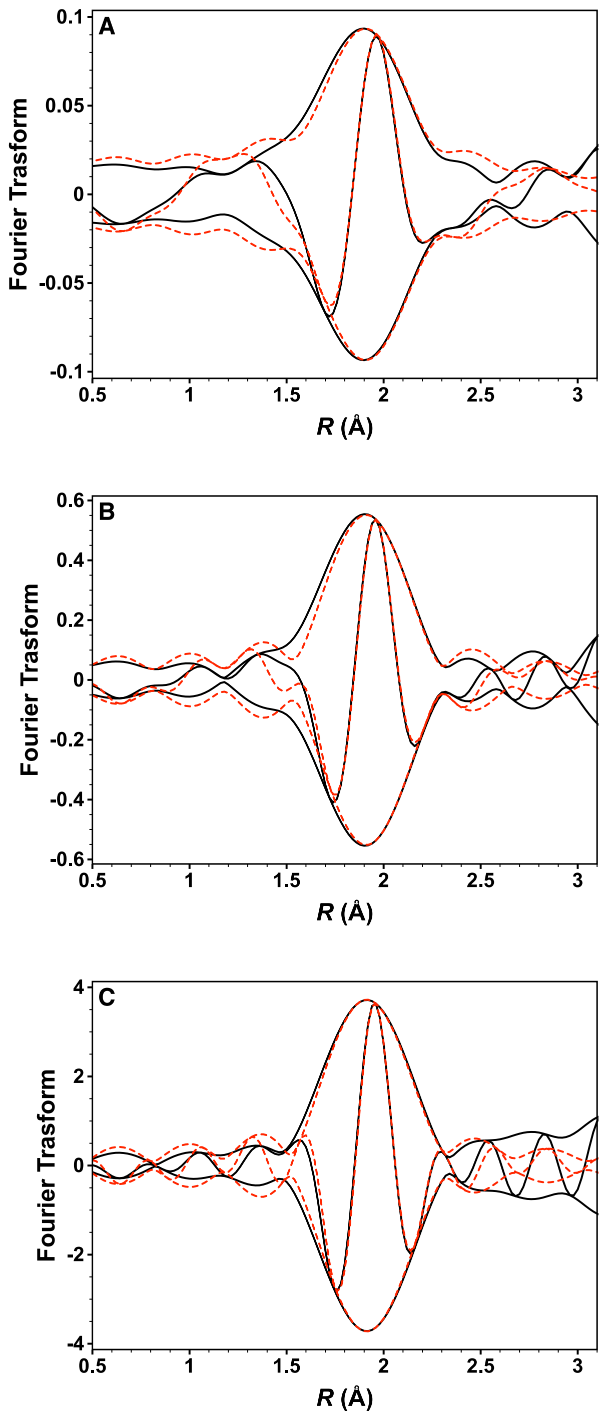

Figure S33. Imaginary part and magnitude of phase- and amplitude-corrected Fourier transform (A, unweighted; B, k^1-weighted; and C, k^2-weighted) of raw data minus calculated Ir−O$_s$, Ir–O$_{CO}$ and Ir–O$_l$ contribution (solid line) and calculated Ir−C$_{CO}$ contribution (dotted line) characterizing the sample formed by the reaction of $Ir(CO)_2(acac)$ with La_2O_3 calcined in flowing O_2 at 873 K.

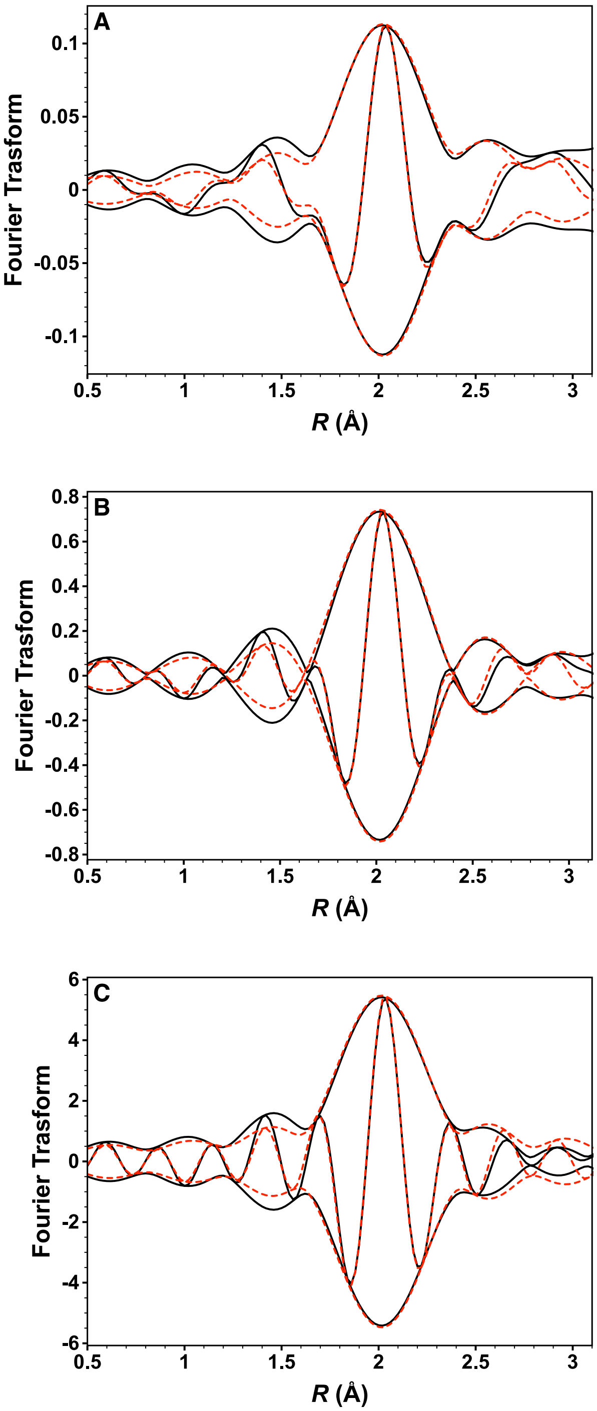

Figure S34. Imaginary part and magnitude of phase- and amplitude-corrected Fourier transform (A, unweighted; B, k^1-weighted; and C, k^2-weighted) of raw data minus calculated Ir–C_{CO}, Ir–O_{CO} and Ir–O_l contribution (solid line) and calculated Ir–O_s contribution (dotted line) characterizing the sample formed by the reaction of $Ir(CO)_2(acac)$ with La_2O_3 calcined in flowing O_2 at 873 K.

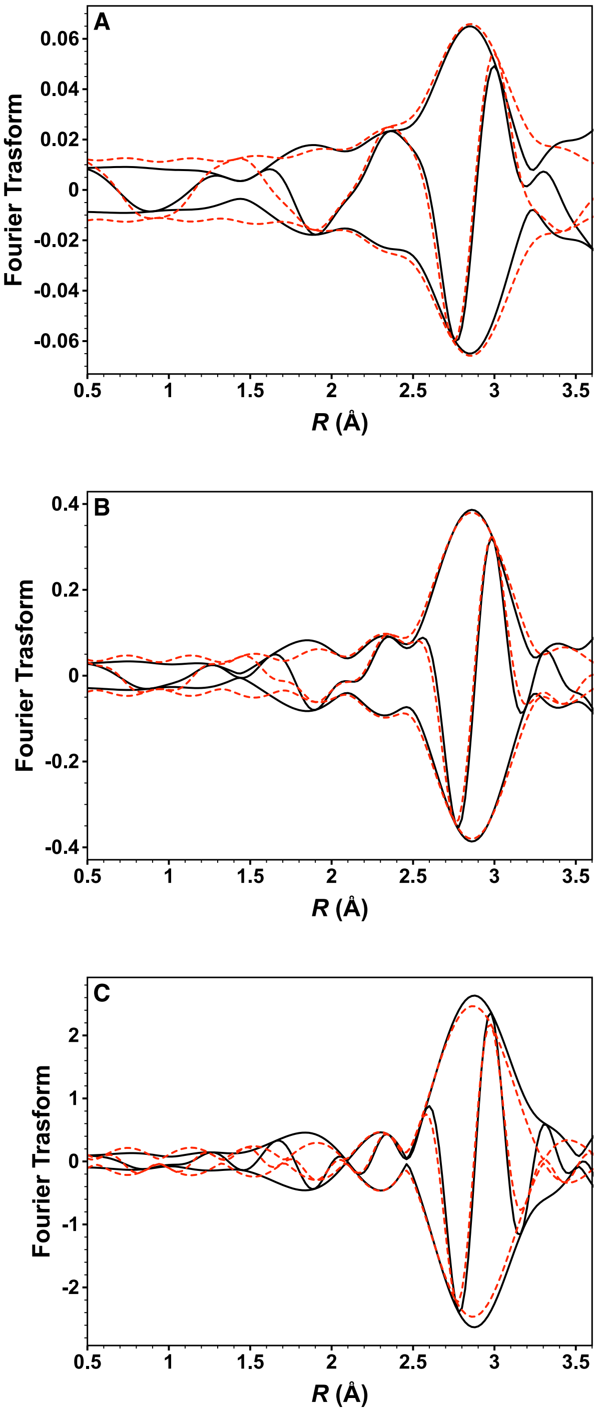

Figure S35. Imaginary part and magnitude of phase- and amplitude-corrected Fourier transform (A, unweighted; B, k^1-weighted; and C, k^2-weighted) of raw data minus calculated Ir–C_{CO}, Ir–O_s and Ir–O_l contribution (solid line) and calculated Ir–O_{CO} contribution (dotted line) characterizing the sample formed by the reaction of $Ir(CO)_2(acac)$ with La_2O_3 calcined in flowing O_2 at 873 K.

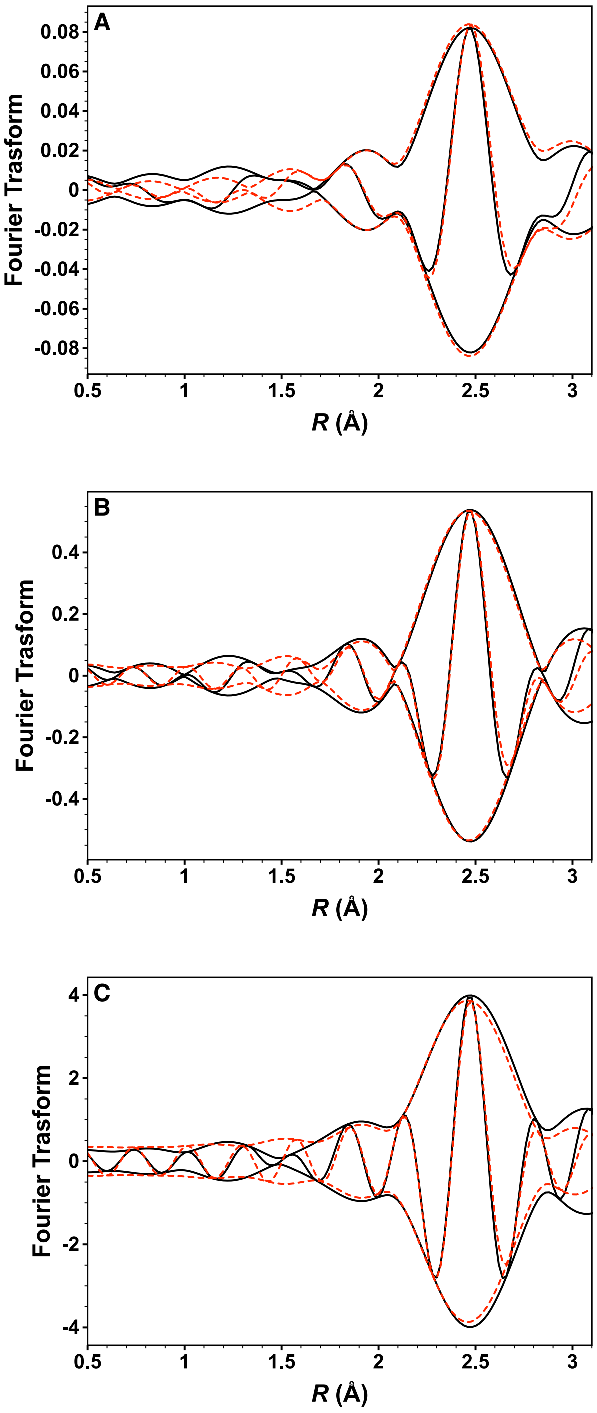

Figure S36. Imaginary part and magnitude of phase- and amplitude-corrected Fourier transform (A, unweighted; B, k^1-weighted; and C, k^2-weighted) of raw data minus calculated Ir−C_{CO}, Ir–O_s and Ir–O_{CO} contribution (solid line) and calculated Ir−O_l contribution (dotted line) characterizing the sample formed by the reaction of $Ir(CO)_2$(acac) with La_2O_3 calcined in flowing O_2 at 873 K.

Table S1. Goodness of fit parameters and variance between data and EXAFS fits for each supported $Ir(CO)_2$ complexes.

Sample	Goodness of fit[a]	k^0 - weighted variance [b] (%)	k^{fit} - weighted variance [b] (%)	Variance in R space,[b, c] with a k^1 weighting (%)	
				Imaginary	Absolute
$Ir(CO)_2/SiO_2$ (Δk = 3.82 – 12.43 Å^{-1})	4.82	2.53	2.53	0.75	0.62
$Ir(CO)_2/TiO_2$ (Δk = 3.17–12.10 Å^{-1})	6.38	28.50	28.50	4.48	2.97
$Ir(CO)_2/Fe_2O_3$ (Δk = 3.92–11.30 Å^{-1})	1.88	21.0	21.0	1.55	0.40
$Ir(CO)_2/CeO_2$ (Δk = 3.92–12.60 Å^{-1})	3.85	5.60	5.60	2.29	0.18
$Ir(CO)_2/MgO$ (Δk = 4.02–11.30 Å^{-1})	3.13	8.94	8.94	4.86	1.89
$Ir(CO)_2/La_2O_3$ (Δk = 3.15–11.30 Å^{-1})	8.65	18.10	18.10	4.61	3.65

[a] Goodness of fit values are calculated by XDAP as follows:

$$goodness\ of\ fit = \frac{\nu}{NPTS\ (\nu - Nfree)} \sum_{i=1}^{NPTS} \left(\frac{\chi exp, i - \chi model, i}{\sigma exp, i} \right)^2$$

[b] Variance between the k-weighted Fourier transform of the data and the fit over the fitting range (1.0–5.0 Å) was calculated by XDAP as follows:

$$k^n\ variance = \frac{\int [k^n(\chi model(k) - \chi \exp(k))]^2 dk}{\int [k^n \chi \exp(k)]^2 dk} \times 100$$

[c] Variance in R space is calculated by XDAP as follows:

$$k^n\ variance = \frac{\int [k^n(FTmodel(R) - FT\exp(R))]^2 dR}{\int [k^n FT\exp(R)]^2 dR} \times 100$$

S2. Infrared Spectroscopy (IR) results

S2.1. Evidence of atomically dispersed iridium complexes supported on metal oxides

Figure 1 presents the IR spectra of the samples. The bands in 1300–1700 cm^{-1} region (given in Table S2) represent acetylacetone (Hacac) formed by the reaction of precursor acac ligands with support hydroxyl groups, forming structures similar to those obtained when Hacac is adsorbed on metal oxides.[1] The 1800–2200 cm^{-1} region of the IR spectrum of the precursor includes bands at 2049 and 1985 cm^{-1} characterizing the symmetric (υ_{sym}(CO)) and asymmetric (υ_{asym}(CO)) stretches of the CO ligands (Figure S37 and Table S2). The weak bands at 2029 and 1950 cm^{-1} represent natural-abundance ^{13}CO.[2] Spectra of the supported iridium complexes indicate distinct bands similar to those of the precursor $Ir(CO)_2$(acac), assigned to the symmetric (υ_{sym}(CO)) and asymmetric (υ_{asym}(CO)) stretches of the CO ligands. We observed no bridging CO bands that would have indicated the presence of iridium clusters (which would have appeared in the range of 1850 to 1950 cm^{-1}).[3] Thus, the IR data match the EXAFS data (Table 1) and show that $Ir(CO)_2$ complexes were bonded to the metal oxides in the absence of detectable iridium clusters.[4-5] Moreover, shoulders at 2028 and 2036 cm^{-1} in the spectra of the Fe_2O_3- and CeO_2-supported samples, respectively, indicate iridium carbonyls on minority surface sites.[2,6]

Table S2. IR band positions of uncoated iridium complexes on various supports and the precursor in the acac region (1300–1700 cm^{-1}).

**Sample**	**Frequencies of IR bands in 1300–1700 cm^{-1} region (cm^{-1})**				
	$\delta_{asym}(CH_3)$	$\upsilon_{asym}(CO)_{ring}$	$\delta_{asym}(CH)$	$\upsilon_{asym}(CCC)_{ring}$	$\upsilon_{sym}(CO)_{ring}$
$Ir(CO)_2$(acac)	1365	1382	1427	1532	1563
$Ir(CO)_2/SiO_2$	1372	1396	1432	1535	1569
$Ir(CO)_2/TiO_2$	1365	1388	1436	1528	1559
$Ir(CO)_2/Fe_2O_3$	1362	1393	1430	1527	1561
$Ir(CO)_2/CeO_2$	1365	1386	1441	1525	1559
$Ir(CO)_2$/MgO	1365	1404	1460	1541	1568
$Ir(CO)_2/La_2O_3$	1361	1393	1450	1540	1568

S3. X-Ray Absorption Near Edge Structure (XANES) Results

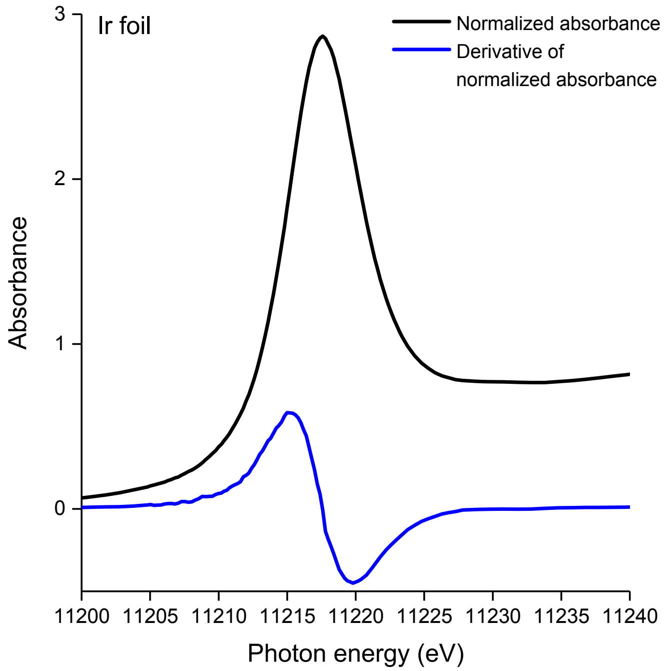

Figure S37. Normalized (black) and derivative of normalized (blue) XANES spectra at the Ir L_{III} edge characterizing iridium foil.

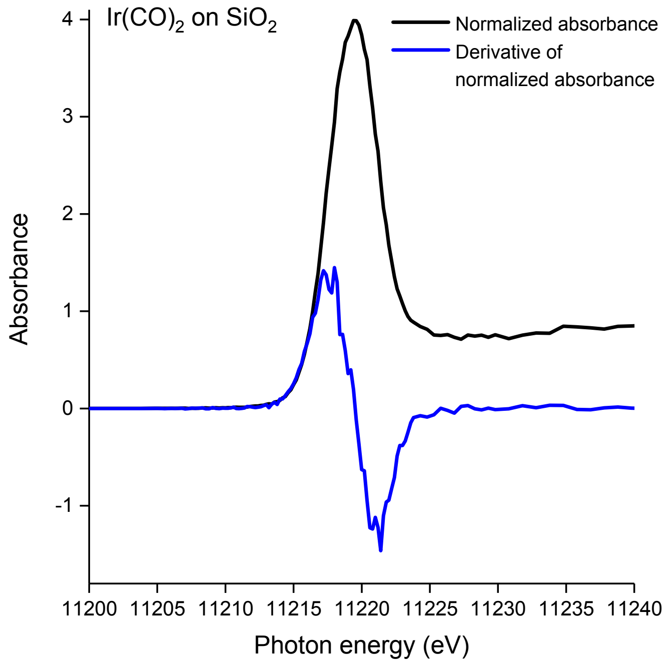

Figure S38. Normalized (black) and derivative of normalized (blue) XANES spectra at the Ir L_{III} edge characterizing uncoated $Ir(CO)_2$ complexes on SiO_2.

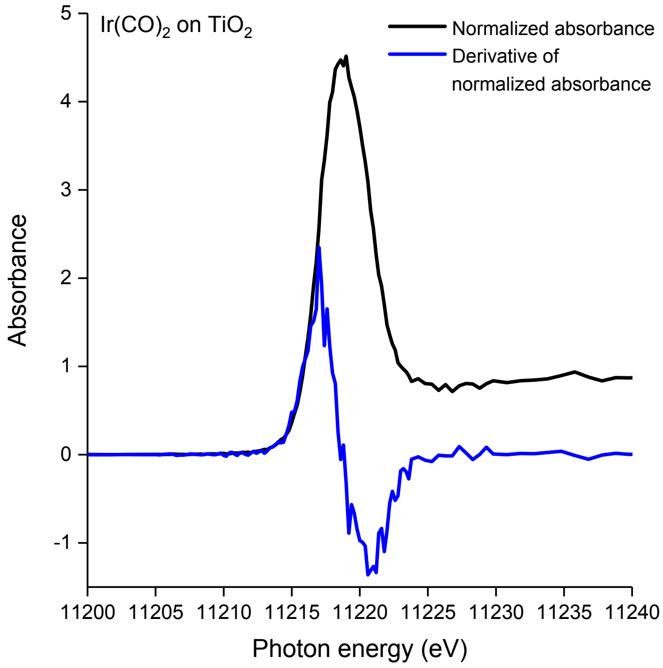

Figure S39. Normalized (black) and derivative of normalized (blue) XANES spectra at the Ir L_{III} edge characterizing uncoated $Ir(CO)_2$ complexes on TiO_2.

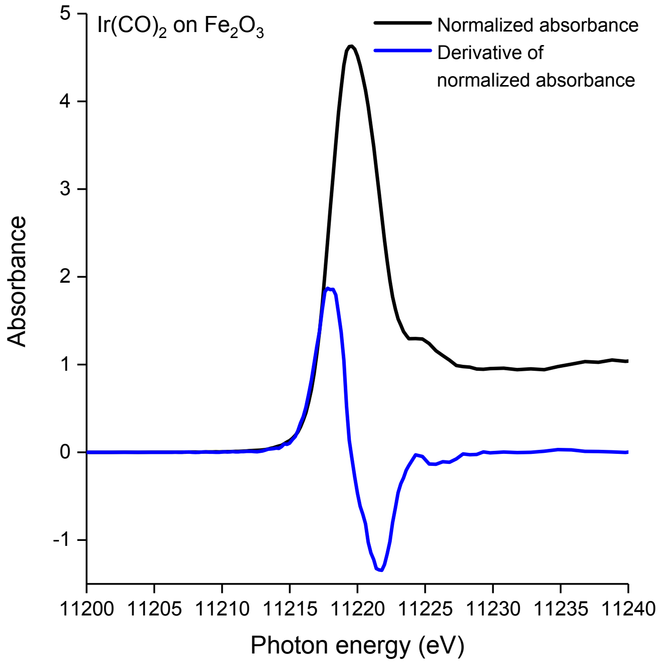

Figure S40. Normalized (black) and derivative of normalized (blue) XANES spectra at the Ir L_{III} edge characterizing uncoated $Ir(CO)_2$ complexes on Fe_2O_3.

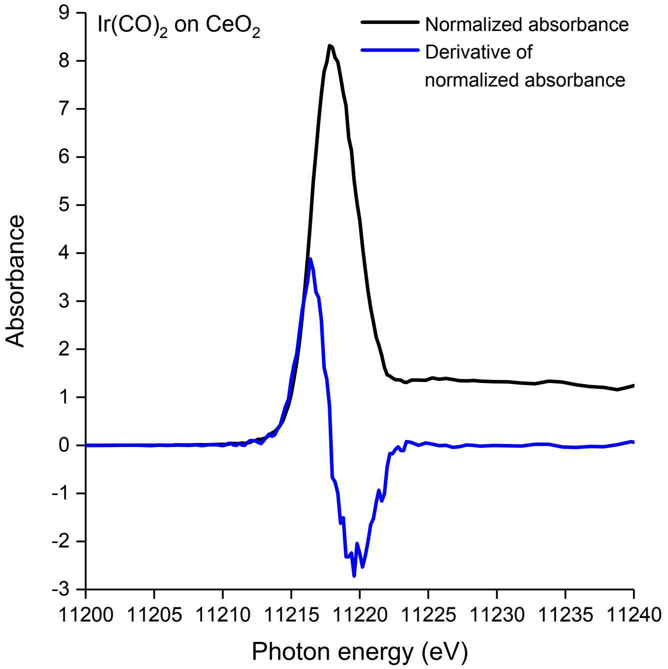

Figure S41. Normalized (black) and derivative of normalized (blue) XANES spectra at the Ir L_{III} edge characterizing uncoated $Ir(CO)_2$ complexes on CeO_2.

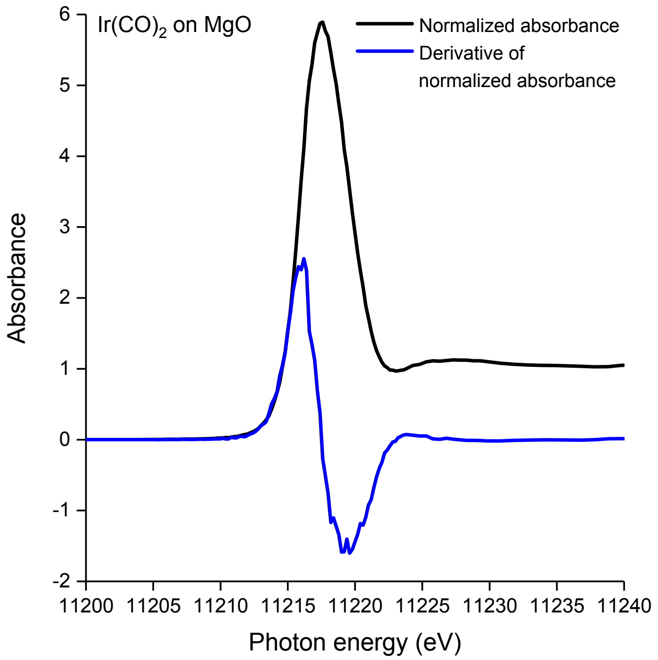

Figure S42. Normalized (black) and derivative of normalized (blue) XANES spectra at the Ir L_{III} edge characterizing uncoated $Ir(CO)_2$ complexes on MgO.

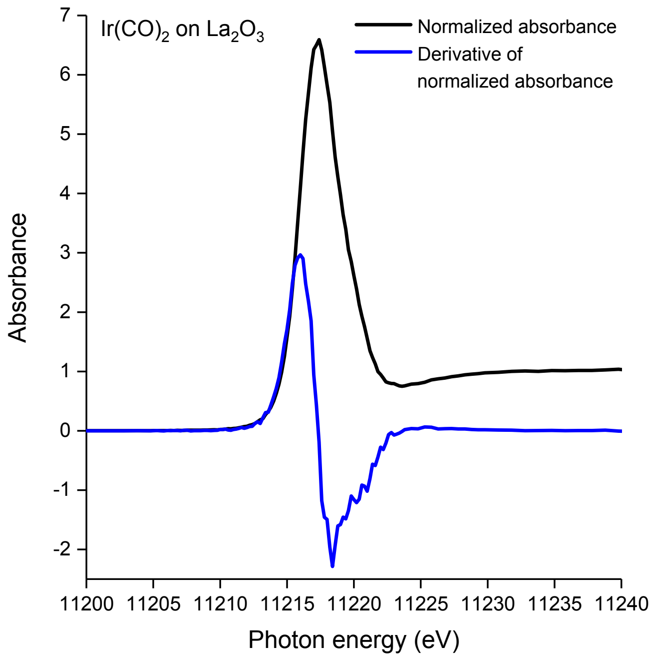

Figure S43. Normalized (black) and derivative of normalized (blue) XANES spectra at the Ir L_{III} edge characterizing uncoated $Ir(CO)_2$ complexes on La_2O_3.

Table S3. Ir L_{III} edge energies of uncoated, [BMIM][BF_4]-coated, [BMIM][Ac]-coated, and [CPMIM][DCA]-coated supported $Ir(CO)_2$ complexes.

**Supported $Ir(CO)_2$ Complex**	**Ir L_{III} edge energy (eV)**
$Ir(CO)_2/SiO_2$	11218.0
$Ir(CO)_2/TiO_2$	11217.0
$Ir(CO)_2/Fe_2O_3$	11217.5
$Ir(CO)_2/CeO_2$	11216.4
$Ir(CO)_2/MgO$	11216.2
$Ir(CO)_2/La_2O_3$	11216.0

S4. Scanning Electron Microscopy Coupled with Energy-Dispersive X-ray Spectroscopy (SEM/EDX) Analysis of Coated Samples Confirming Uniform Distribution of IL Molecules

The IL loadings of the samples, prepared by bringing each supported iridium complex in contact with the respective IL and then adding excess calcined support to absorb the excess IL, were set to 20 wt% (and the corresponding Ir loading was 0.4 wt%). To get an indication of the uniformity of the IL coatings on the scale of SEM/EDX images, we collected data for a representative [BMIM][BF_4]-coated sample set (Figure S44); the distribution of fluorine atoms on the scale of the images confirms that the IL was distributed uniformly over the surface. Assuming that this result can be generalized, the average spacing of the Ir atoms was estimated in the range of 1–3 nm, depending on the support's surface area. SEM/EDX images (Table S4 and Figures S45–S50) indicate that the IL loadings ranged from 8–25 wt% in the respective imaged regions. Accordingly, these Ir and IL loadings correspond to approximately 55 ± 3 IL molecules per Ir site with a maximum of a few molecular layers of IL layer. We infer that these values are sufficient to ensure the availability of at least one IL molecule in the vicinity of each Ir center assuming a uniform Ir site distribution over the supports.

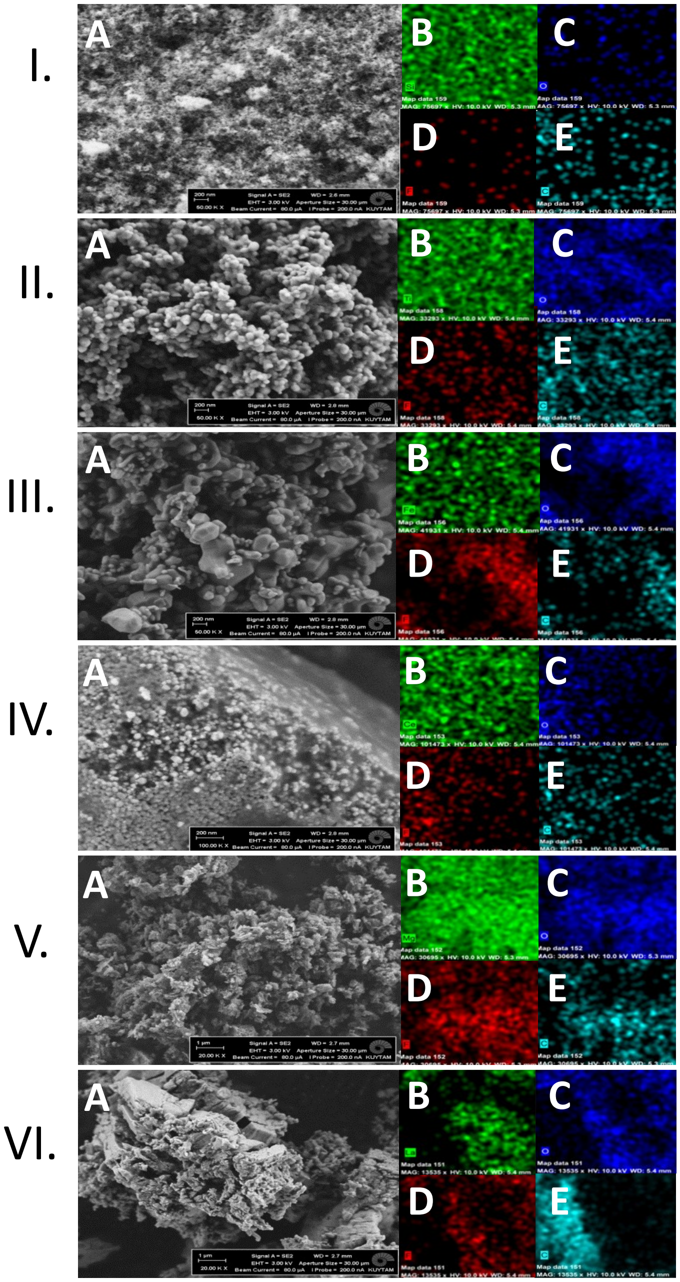

Figure S44. SEM images characterizing a representative sample set of [BMIM][BF_4]-coated Ir$(CO)_2$ complexes supported on various metal oxides: SiO_2 (IA), TiO_2 (IIA), Fe_2O_3 (IIIA), CeO_2 (IVA), MgO (VA), La_2O_3 (VIA). EDX map showing the distribution of the individual elements Si (IB), Ti (IIB), Fe (IIIB), Ce (IVB), Mg (VIB), La (VIIB), O (IC, IIC, IIIC, IVC, VC, VIC), F (ID, IID, IIID, IVD, VD, VID), and C (IE, IIE, IIIE, IVE, VE, VIE). EDX spectra of IL-coated supported Ir$(CO)_2$ complexes are given in Figures S45–S50 in the ESI.

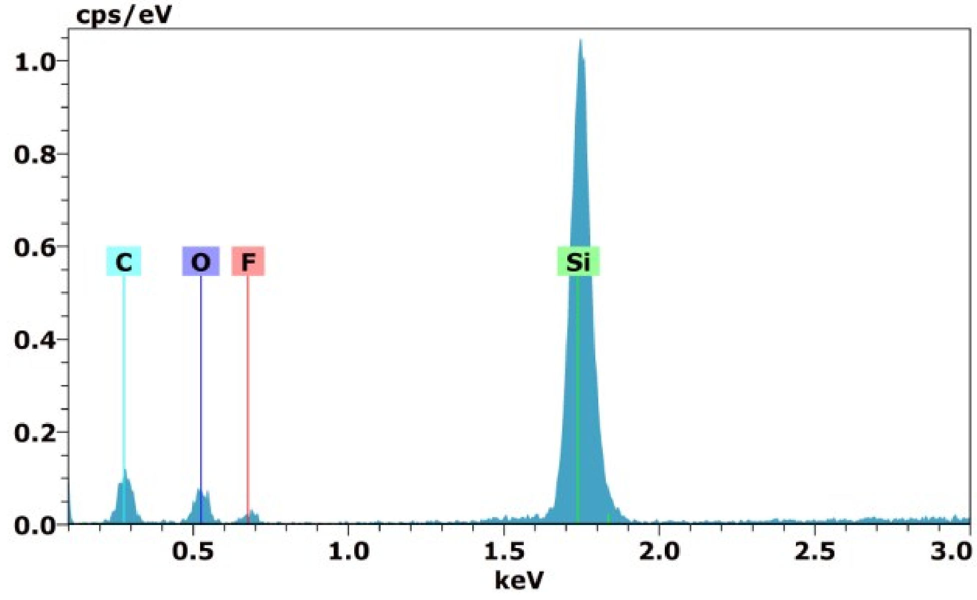

Figure S45. EDX spectra of [BMIM][BF_4]-coated $Ir(CO)_2/SiO_2$.

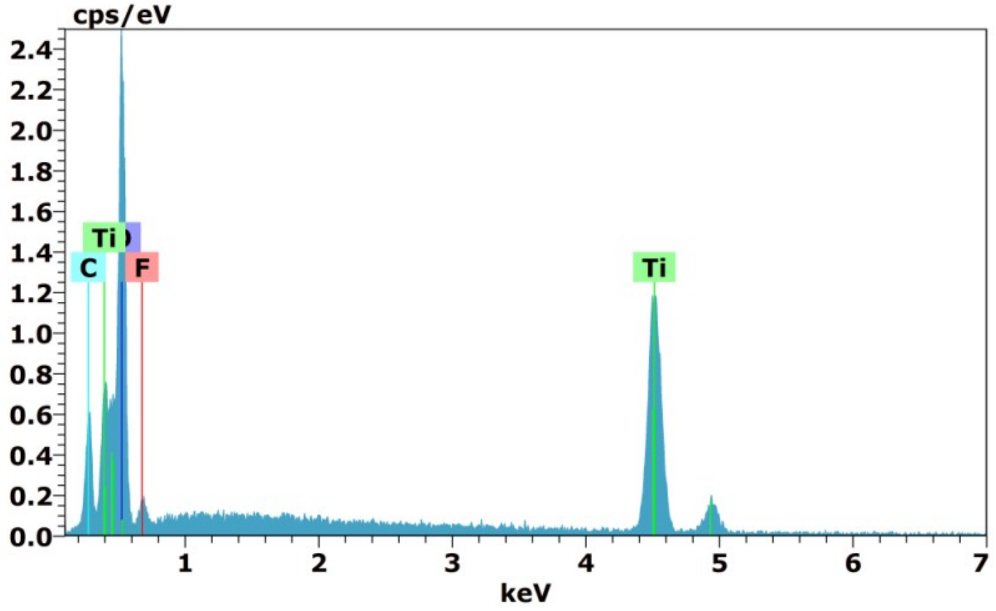

Figure S46. EDX spectra of [BMIM][BF_4]-coated $Ir(CO)_2/TiO_2$.

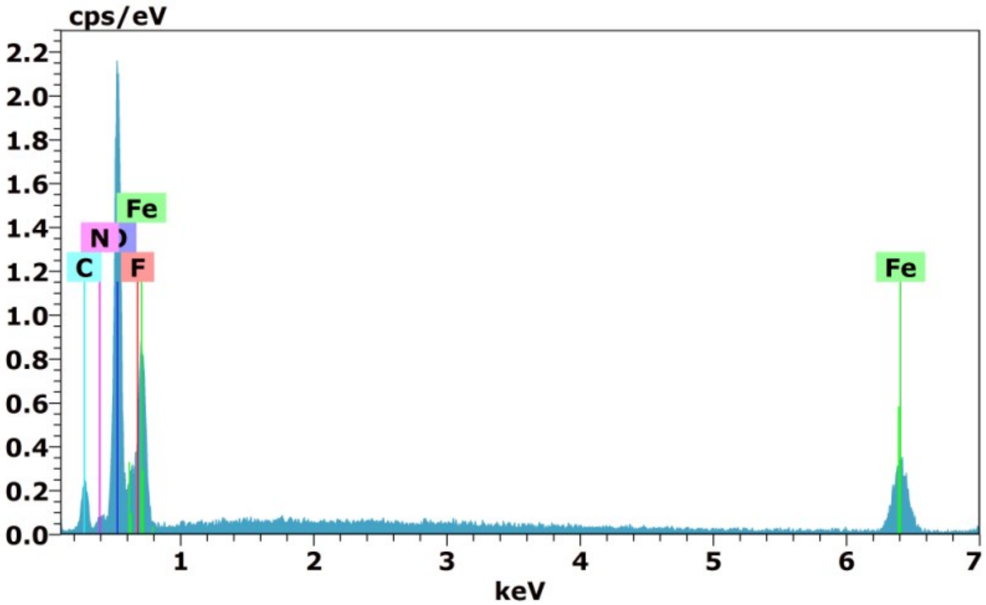

Figure S47. EDX spectra of [BMIM][BF_4]-coated $Ir(CO)_2/Fe_2O_3$.

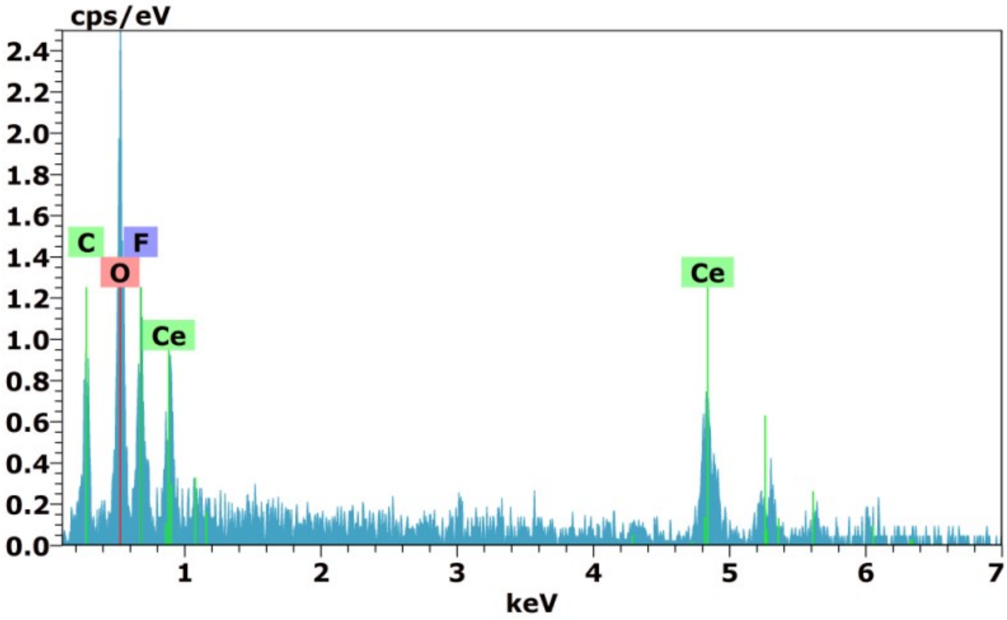

Figure S48. EDX spectra of [BMIM][BF_4]-coated $Ir(CO)_2/CeO_2$.

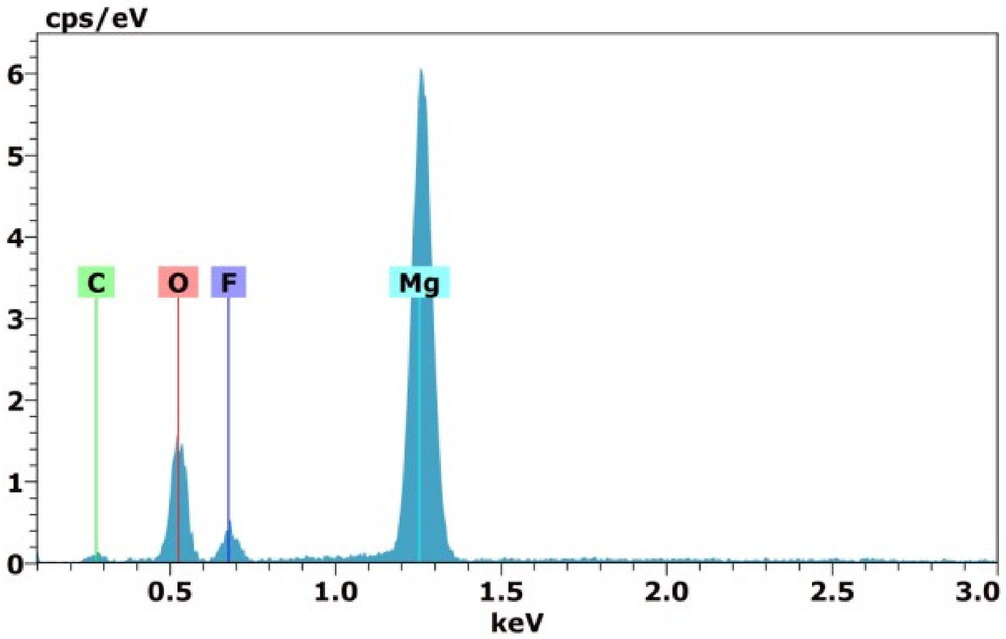

Figure S49. EDX spectra of [BMIM][BF_4]-coated $Ir(CO)_2$/MgO.

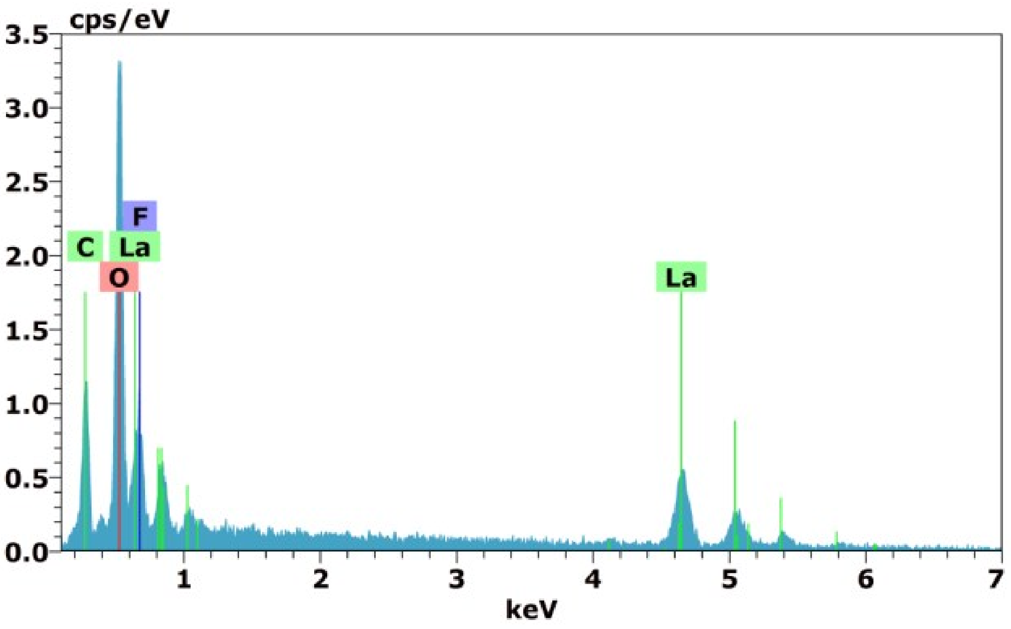

Figure S50. EDX spectra of [BMIM][BF_4]-coated $Ir(CO)_2$/La_2O_3.

Table S4. [BMIM][BF_4] loading amount on supported $Ir(CO)_2$ complexes. These values are calculated by using integrals of EDX spectra area of each sample.

Samples	Calculated IL loading (%)
[BMIM][BF_4]-coated $Ir(CO)_2/SiO_2$	23
[BMIM][BF_4]-coated $Ir(CO)_2/TiO_2$	18
[BMIM][BF_4]-coated $Ir(CO)_2/Fe_2O_3$	8
[BMIM][BF_4]-coated $Ir(CO)_2/CeO_2$	16
[BMIM][BF_4]-coated $Ir(CO)_2/MgO$	22
[BMIM][BF_4]-coated $Ir(CO)_2/La_2O_3$	24

S5. IR evidence of atomically dispersed supported iridium complexes after IL coating

IR spectra comparing the features of the uncoated and IL-coated samples along with those of the bulk ILs are shown in Figure 1 and Figures S51–S53. The data confirm presence of the IL and show that the supported complexes were still present as site-isolated iridium *gem*-dicarbonyls. The spectra of the IL-coated samples, like those of the uncoated samples, are characterized by two distinct carbonyl stretching bands of the supported iridium species.

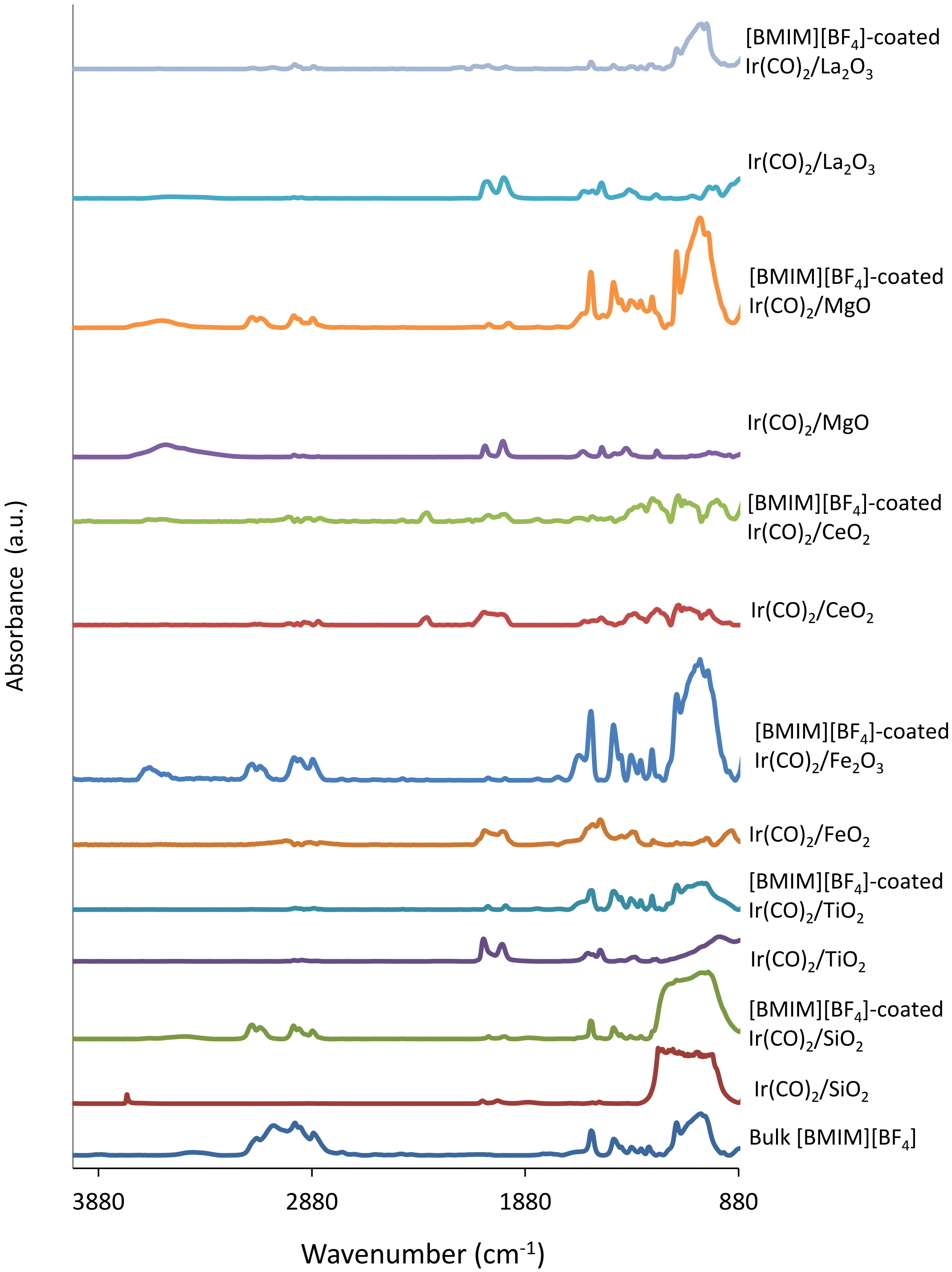

Figure S51. IR data comparing the structural features of bulk [BMIM][BF_4] with those of coated and uncoated Ir$(CO)_2$ complexes prepared on different supports.

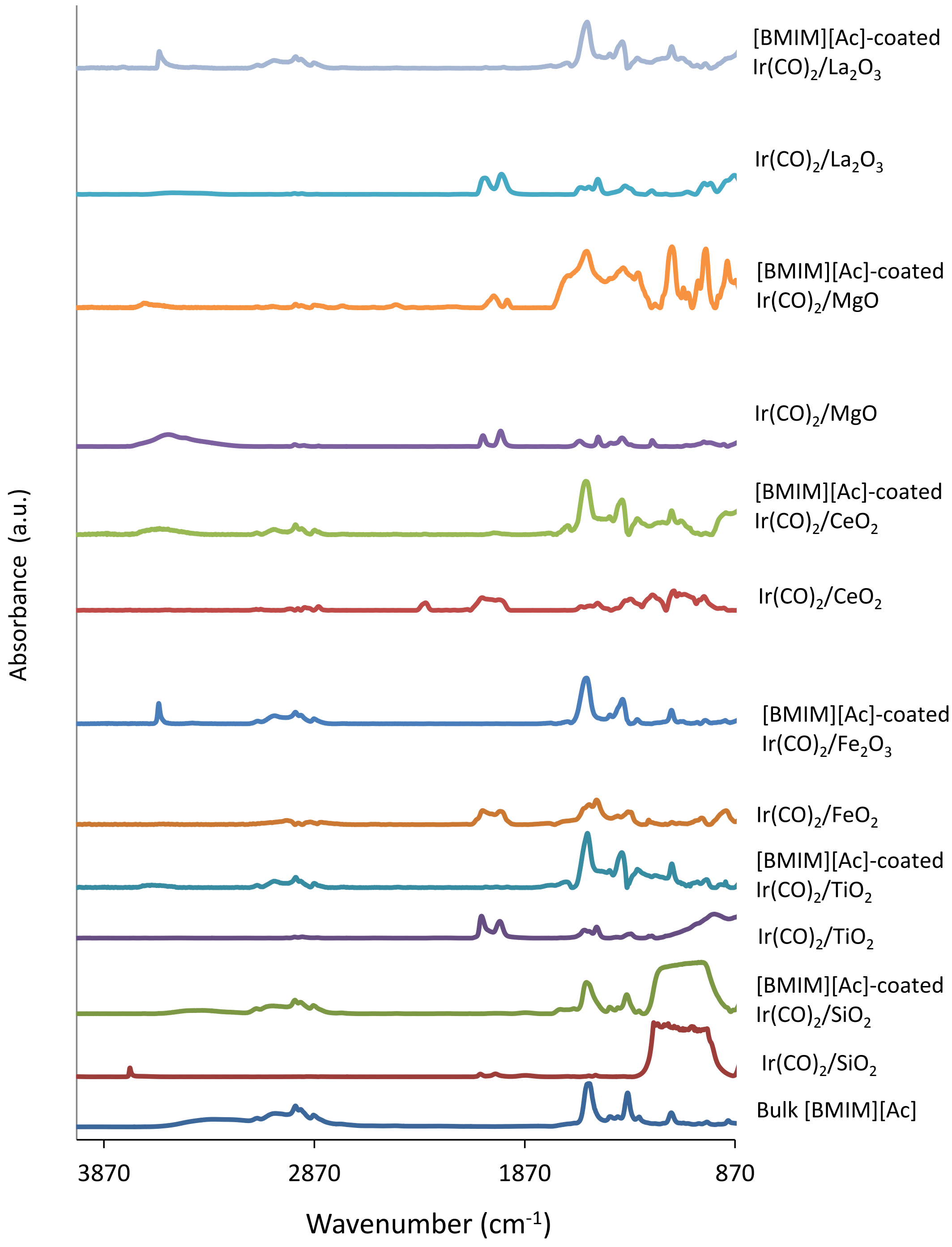

Figure S52. IR data comparing the structural features of bulk [BMIM][Ac] with those of coated and uncoated $Ir(CO)_2$ complexes prepared on different supports.

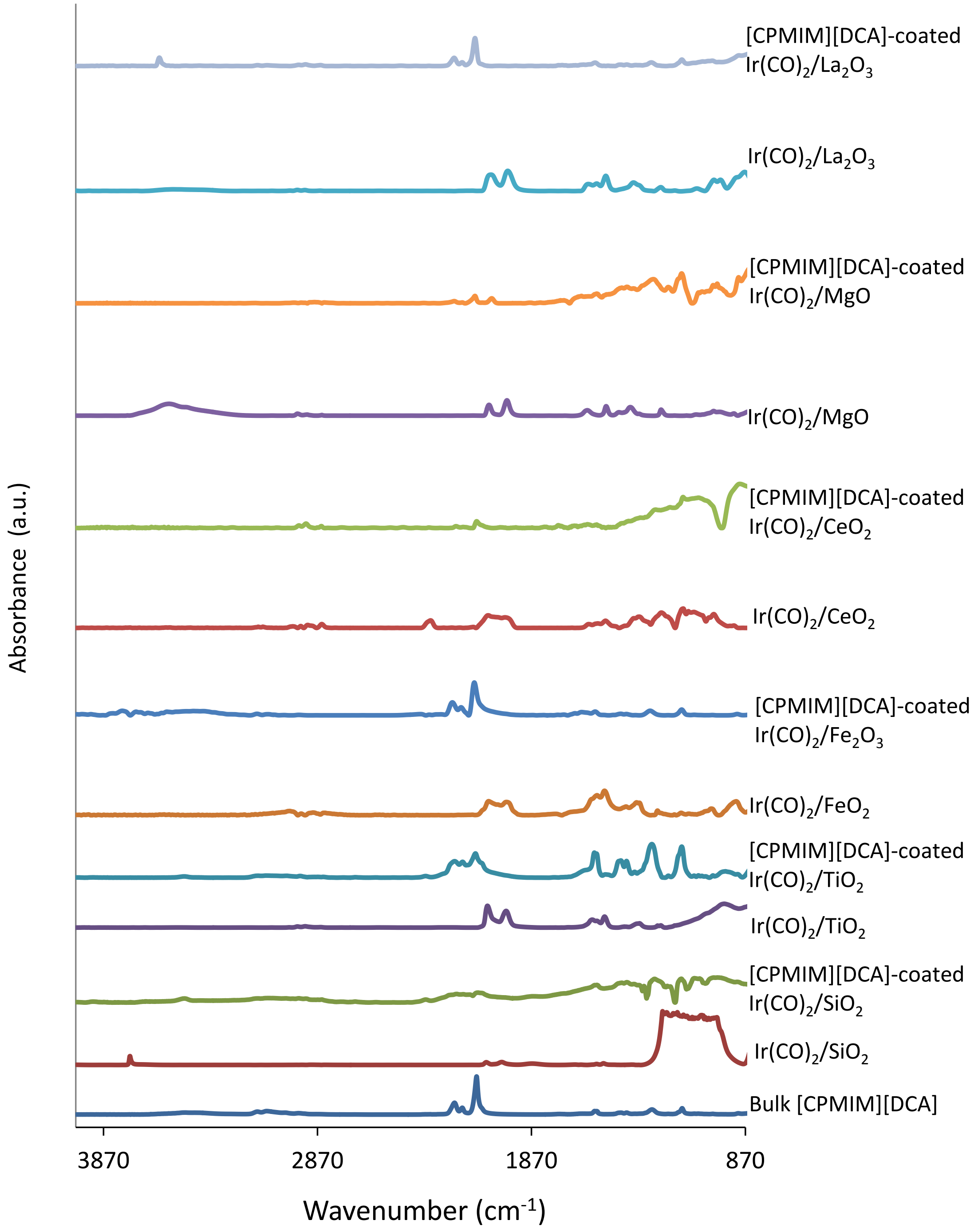

Figure S53. IR data comparing the structural features of bulk [CPMIM][DCA] with those of coated and uncoated $Ir(CO)_2$ complexes prepared on different supports.

S6. Evidence Confirming Iridium Complexes Remained Bonded to Supports in the Presence of ILs

To rule out the possibility that the iridium complex was removed from the surface and dissolved in the IL coating, we washed some IL-coated samples in methanol and checked the IR spectra of the filtrates and the washed samples. No vibrational bands associated with iridium carbonyls were detected in the filtrates, and the spectra of the washed samples still indicated the presence of the iridium carbonyls (although the peak positions and shapes were changed by the treatments, as illustrated in Figure S54 for a representative sample [BMIM][BF_4]-coated $Ir(CO)_2/MgO$). These results show that the iridium complexes remained bonded to the support even after the samples were coated with the ILs and did not dissolve in the IL.

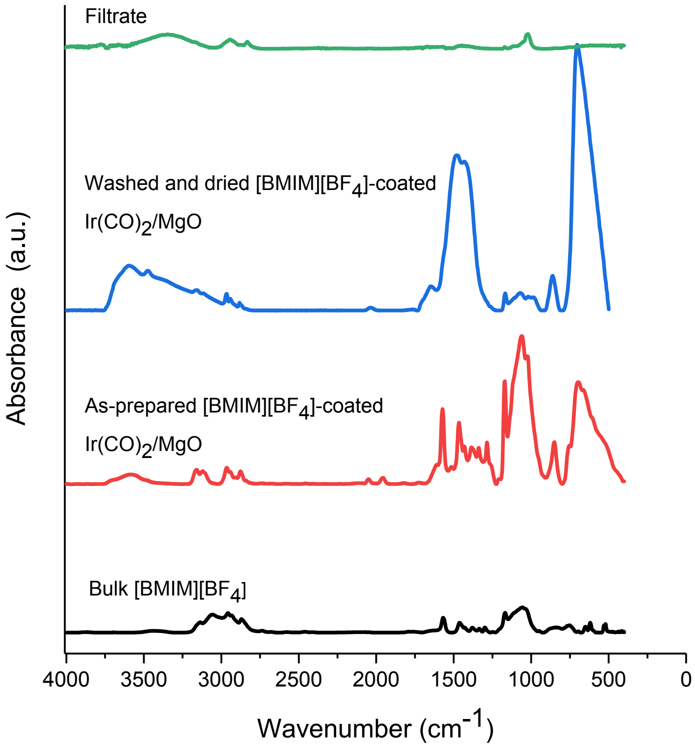

Figure S54. IR spectra characterizing the [BMIM][BF_4]-coated $Ir(CO)_2/MgO$ sample before and after washing with methanol: Bulk [BMIM][BF_4] (black); as-prepared [BMIM][BF_4]-coated $Ir(CO)_2/MgO$ (red); washed (in methanol) and dried (at 353 K in air) [BMIM][BF_4]-coated $Ir(CO)_2/MgO$ (blue); and the filtrate of washing (green).

S7. Details on Catalytic hydrogenation of 1,3-butadiene

Each catalyst was first activated by removal of one of the CO ligands per each Ir atom by treatment in flowing ethylene at 373 K; this treatment incorporated ethylene ligands in place of CO, which is a strong catalytic reaction inhibitor,[7] as reported for similar metal carbonyl complexes supported on metal oxides and zeolites.[7-8]

IR spectra recorded before and after ethylene exposure (Figures S55-S77), indicate changes in the intensities and positions of the υ(CO) bands as well as the formation of new bands, but none indicating the formation of carbonyls bonded to neighboring iridium centers, which would have indicated iridium cluster formation.[9] New bands located between the two carbonyl stretching bands are consistent with the partial replacement of CO with ethylene.[7-8] For example, in the spectrum of the TiO_2-supported iridium complex coated with [BMIM][BF_4], the intensity of the

υ_{asym}(CO) band at 1988 cm^{-1} decreased slightly relative to that of the υ_{sym}(CO) band, accompanied by the appearance of two new bands, at 2030 and 2048 cm^{-1}, assigned to Ir(CO)(C_2H_4) and Ir(CO)(C_2H_4)$_2$, respectively.[5,10] The areas of the υ(CO) bands indicate that the supported complexes were converted into mixtures of Ir(CO)$_2$ (~74%), Ir(CO)(C_2H_4) (~16%), and Ir(CO)(C_2H_4)$_2$ (~10%) on TiO_2, for example. Data characterizing other samples indicate the presence of mixtures of Ir(CO)$_2$, Ir(CO)(C_2H_4), and Ir(CO)(C_2H_4)$_2$ in varying concentrations (Table S5).

Table S5. Relative concentrations (%) of the supported Ir(CO)$_2$, Ir(CO)(C_2H_4), and Ir(CO)(C_2H_4)$_2$ species formed by treatment of the IL-coated and uncoated supported Ir(CO)$_2$ complexes in flowing C_2H_4 at 373 K and their corresponding υ(CO) values given in parentheses in units of cm^{-1}.

**Supported Ir(CO)$_2$ Complex**	**Percentages of species after ethylene treatment (%) with corresponding υ_{sym}(CO) and υ_{asym}(CO) (cm^{-1}) band positions**		
	**Ir(CO)$_2$***	**Ir(CO)(C_2H_4)**	**Ir(CO)(C_2H_4)$_2$****
Ir(CO)$_2$/SiO_2	78 (2081, 2008)	22 (2045)	ND
Ir(CO)$_2$/TiO_2	74 (2076, 1988)	16 (2030)	10 (2048)
Ir(CO)$_2$/Fe_2O_3	55 (2073, 1979)	41 (2034)	4 (2052)
Ir(CO)$_2$/CeO_2	68 (2071, 2057)	16 (2029)	16 (2041)
Ir(CO)$_2$/MgO	75 (2069, 1985)	17 (2032)	8 (2056)
Ir(CO)$_2$/La_2O_3	68 (2066, 1977)	23 (2020)	9 (2051)
[BMIM][BF_4]-coated Ir(CO)$_2$/SiO_2	71 (2052, 1981)	29 (2031)	ND
[BMIM][BF_4]-coated Ir(CO)$_2$/TiO_2	64 (2055, 1971)	23 (2027)	13 (2041)
[BMIM][BF_4]-coated Ir(CO)$_2$/Fe_2O_3	59 (2056, 1969)	41 (2024)	ND
[BMIM][BF_4]-coated Ir(CO)$_2$/CeO_2	86 (2057, 1974)	12 (2021)	2 (2042)
[BMIM][BF_4]-coated Ir(CO)$_2$/MgO	49 (2058, 1960)	51 (2024)	ND
[BMIM][BF_4]-coated Ir(CO)$_2$/La_2O_3	61 (2055, 1972)	39 (2018)	ND
[BMIM][Ac]-coated Ir(CO)$_2$/SiO_2	81 (2049, 1953)	19 (2039)	ND
[BMIM][Ac]-coated Ir(CO)$_2$/TiO_2	57 (2052, 1962)	43 (2018)	ND
[BMIM][Ac]-coated Ir(CO)$_2$/Fe_2O_3	83 (2053, 1947)	17 (2021)	ND
[BMIM][Ac]-coated Ir(CO)$_2$/CeO_2	47 (2050, 1966)	38 (2027)	15 (2038)
[BMIM][Ac]-coated Ir(CO)$_2$/MgO	75 (2056, 1956)	25 (2008)	ND
[BMIM][Ac]-coated Ir(CO)$_2$/La_2O_3	95 (2053, 1970)	5 (2019)	ND
[CPMIM][DCA]-coated Ir(CO)$_2$/SiO_2	93 (2098, 1974)	7 (2035)	ND
[CPMIM][DCA]-coated Ir(CO)$_2$/TiO_2	91 (2084, 1956)	9 (2023)	ND
[CPMIM][DCA]-coated Ir(CO)$_2$/Fe_2O_3	96 (2081, 1973)	4 (2032)	ND
[CPMIM][DCA]-coated Ir(CO)$_2$/CeO_2	89 (2074, 1963)	ND	11 (2045)
[CPMIM][DCA]-coated Ir(CO)$_2$/MgO	76 (2064, 1959)	24 (2015)	ND
[CPMIM][DCA]-coated Ir(CO)$_2$/La_2O_3	88 (2061, 1973)	12 (2020)	ND

*Value calculated on the basis of sum of the intensities of υ_{sym}(CO) and υ_{asym}(CO).

**ND: not detected.

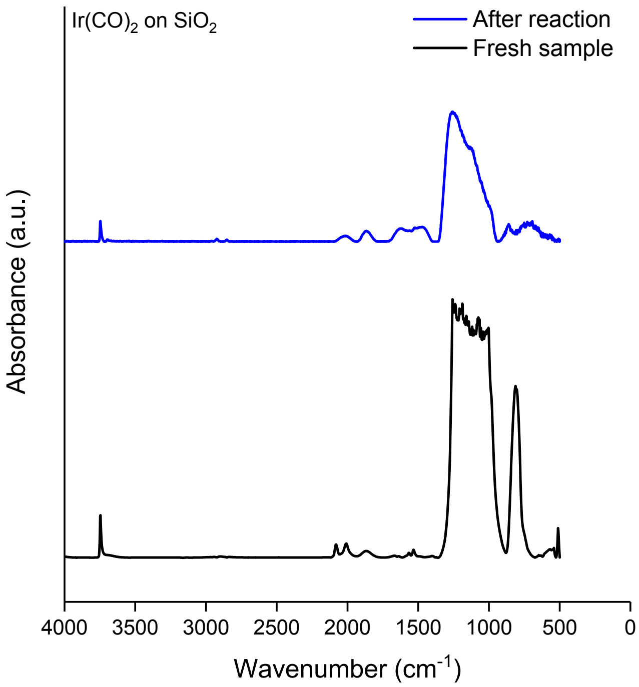

Figure S55. IR spectra of uncoated $Ir(CO)_2$ complexes on SiO_2 before (black) and after (blue) ethylene exposure.

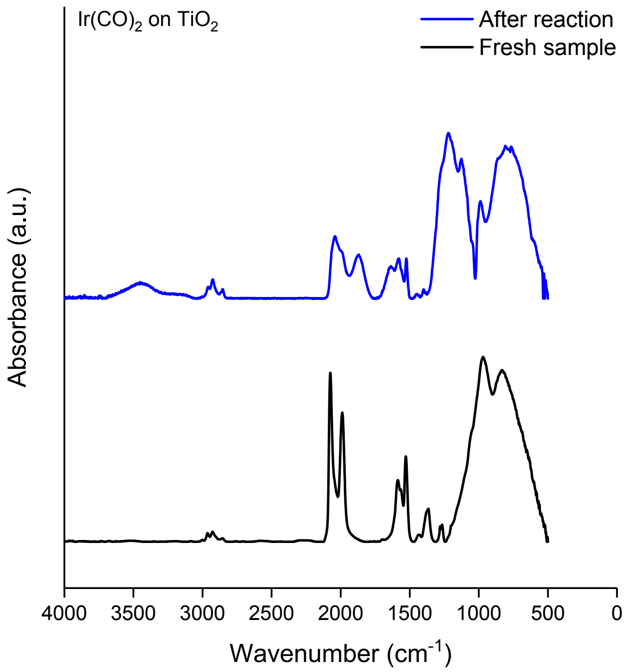

Figure S56. IR spectra of uncoated $Ir(CO)_2$ complexes on TiO_2 before (black) and after (blue) ethylene exposure.

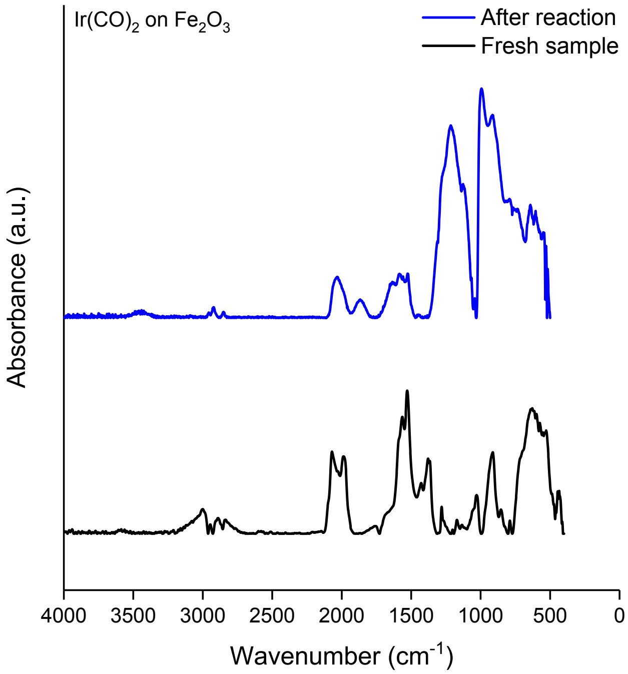

Figure S57. IR spectra of uncoated $Ir(CO)_2$ complexes on Fe_2O_3 before (black) and after (blue) ethylene exposure.

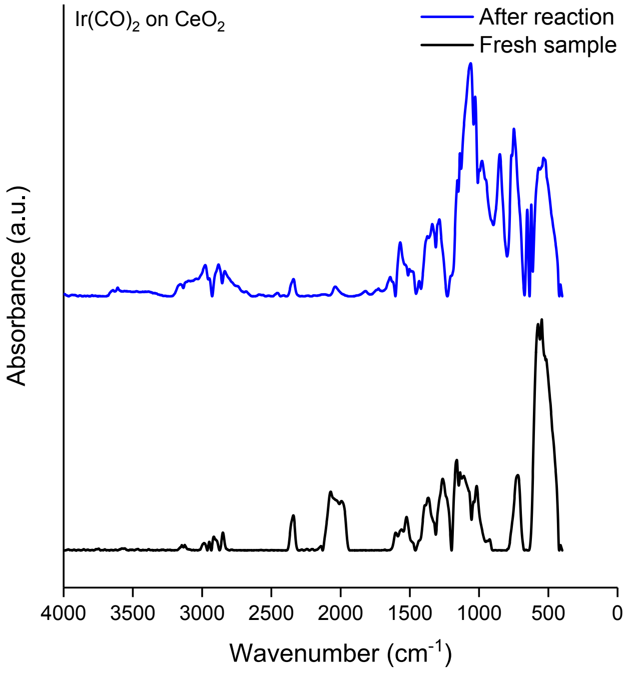

Figure S58. IR spectra of uncoated $Ir(CO)_2$ complexes on CeO_2 before (black) and after (blue) ethylene exposure.

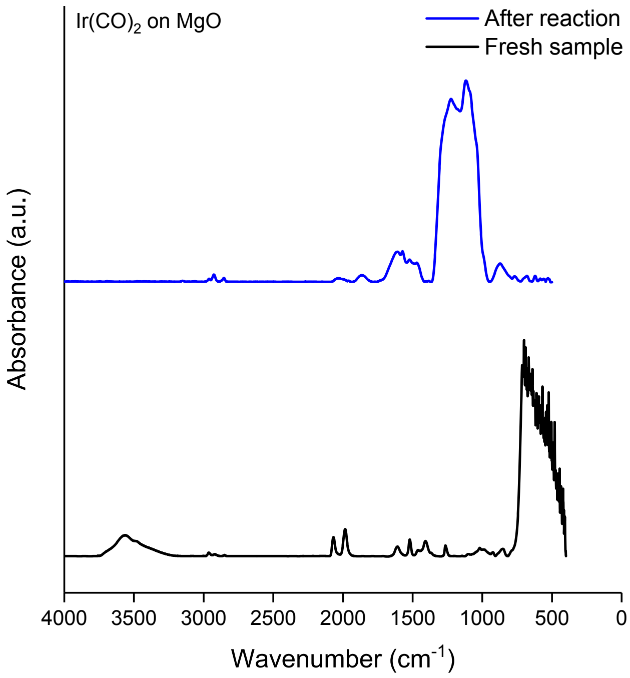

Figure S59. IR spectra of uncoated $Ir(CO)_2$ complexes on MgO before (black) and after (blue) ethylene exposure.

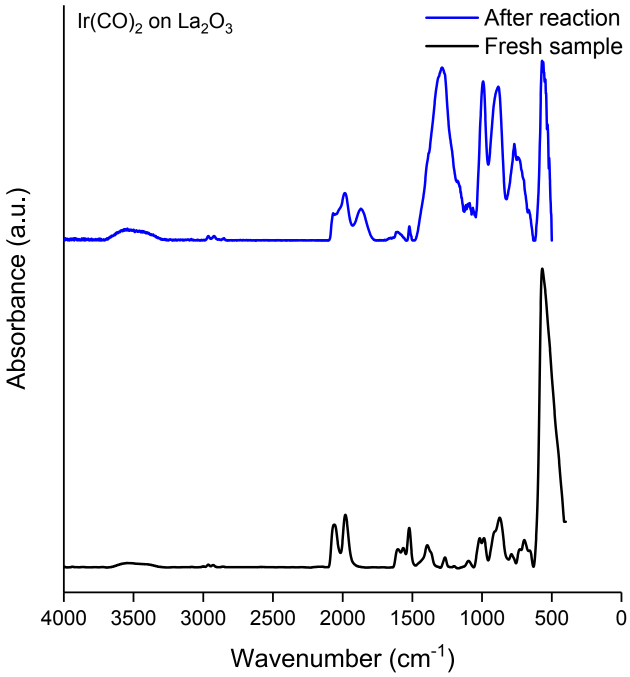

Figure S60. IR spectra of uncoated $Ir(CO)_2$ complexes on La_2O_3 before (black) and after (blue) ethylene exposure.

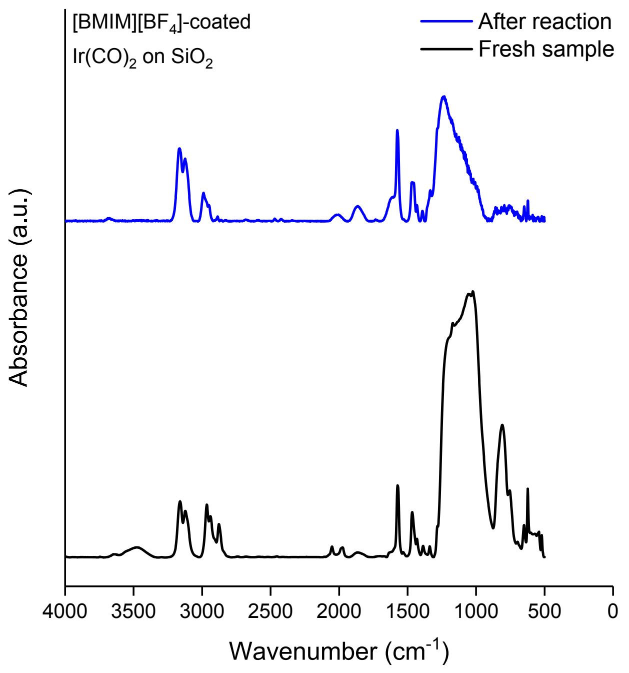

Figure S61. IR spectra of [BMIM][BF_4]-coated Ir$(CO)_2$ complexes on SiO_2 before (black) and after (blue) ethylene exposure.

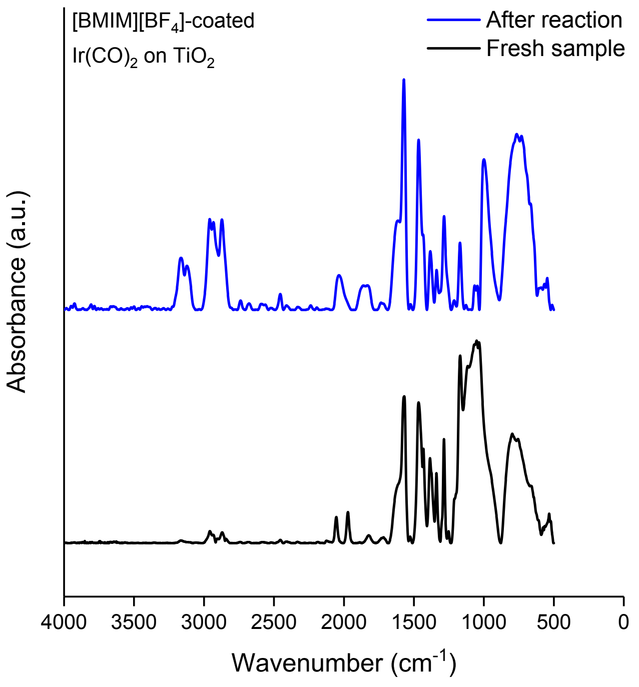

Figure S62. IR spectra of [BMIM][BF_4]-coated Ir$(CO)_2$ complexes on TiO_2 before (black) and after (blue) ethylene exposure.

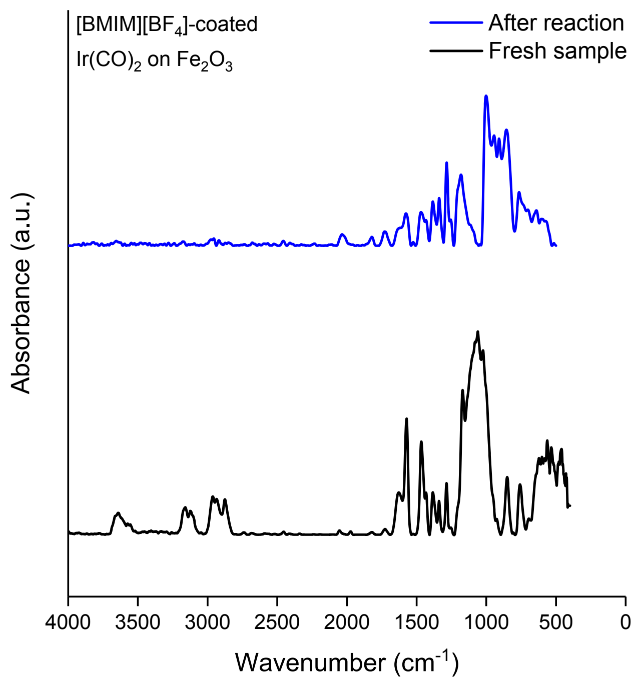

Figure S63. IR spectra of [BMIM][BF_4]-coated Ir$(CO)_2$ complexes on Fe_2O_3 before (black) and after (blue) ethylene exposure.

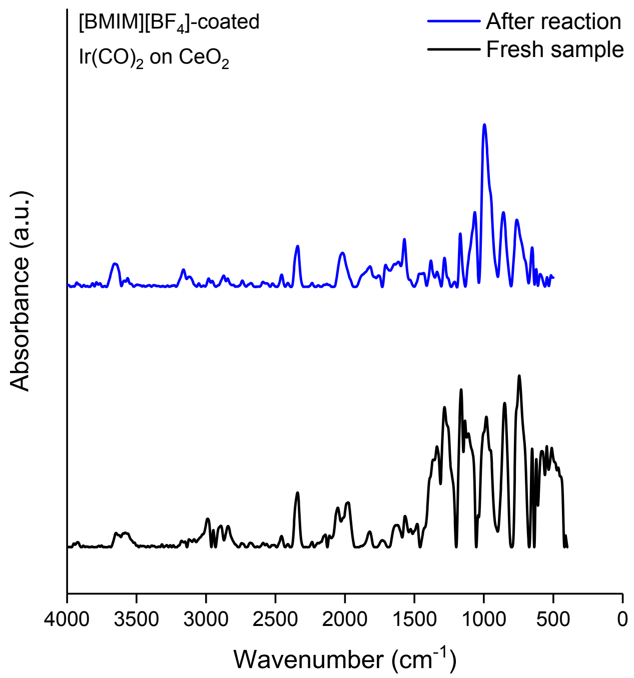

Figure S64. IR spectra of [BMIM][BF_4]-coated Ir$(CO)_2$ complexes on CeO_2 before (black) and after (blue ethylene exposure.

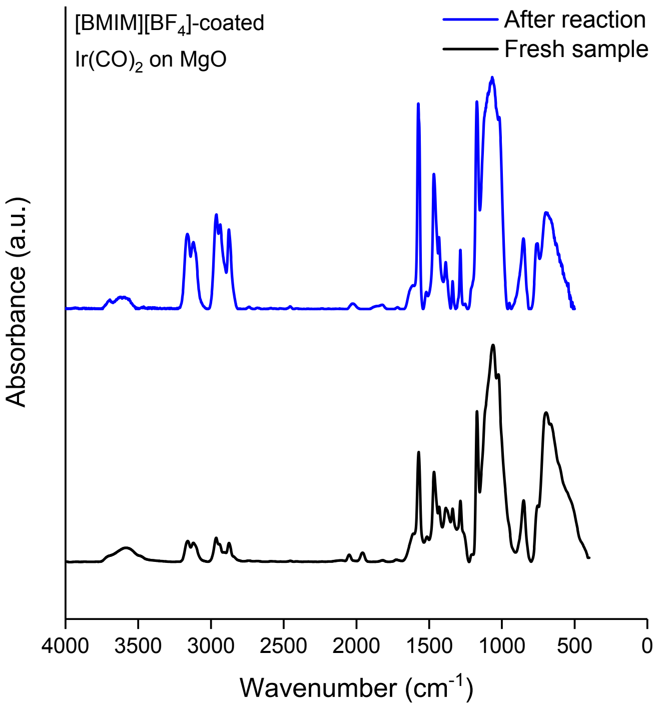

Figure S65. IR spectra of [BMIM][BF_4]-coated $Ir(CO)_2$ complexes on MgO before (black) and after (blue) ethylene exposure.

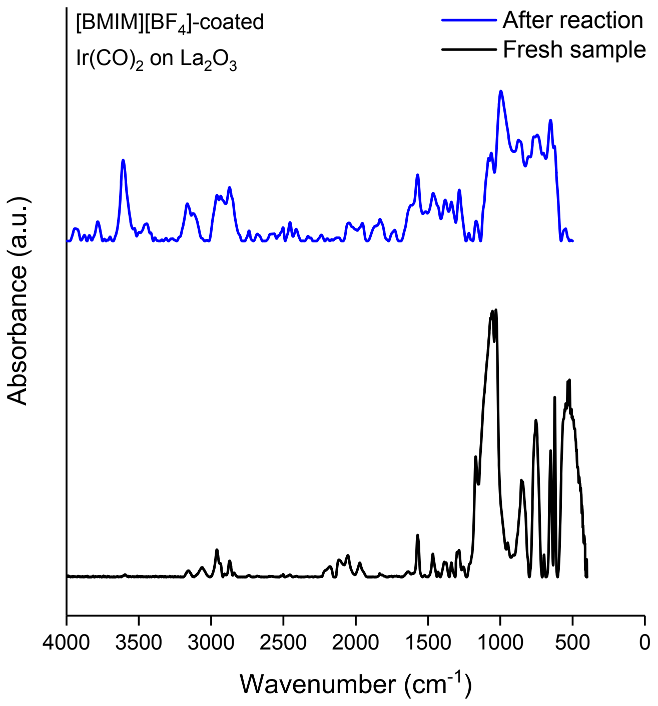

Figure S66. IR spectra of [BMIM][BF_4]-coated $Ir(CO)_2$ complexes on La_2O_3 before (black) and after (blue) ethylene exposure.

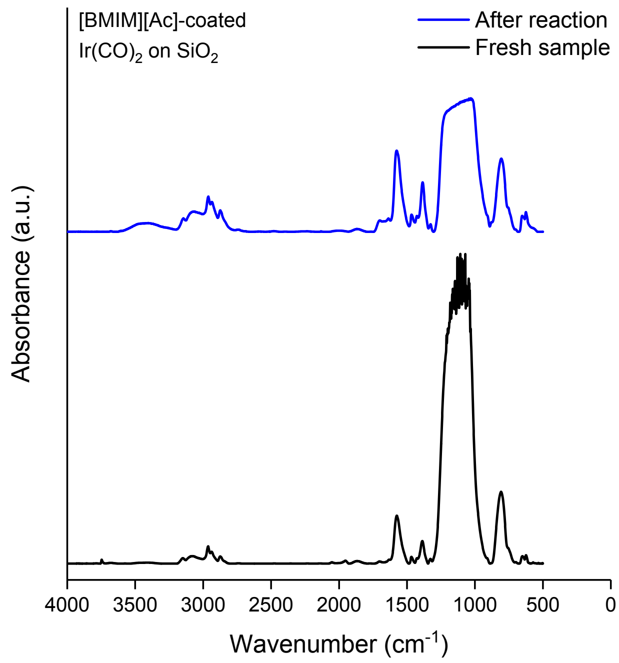

Figure S67. IR spectra of [BMIM][Ac]-coated Ir$(CO)_2$ complexes on SiO_2 before (black) and after (blue) ethylene exposure.

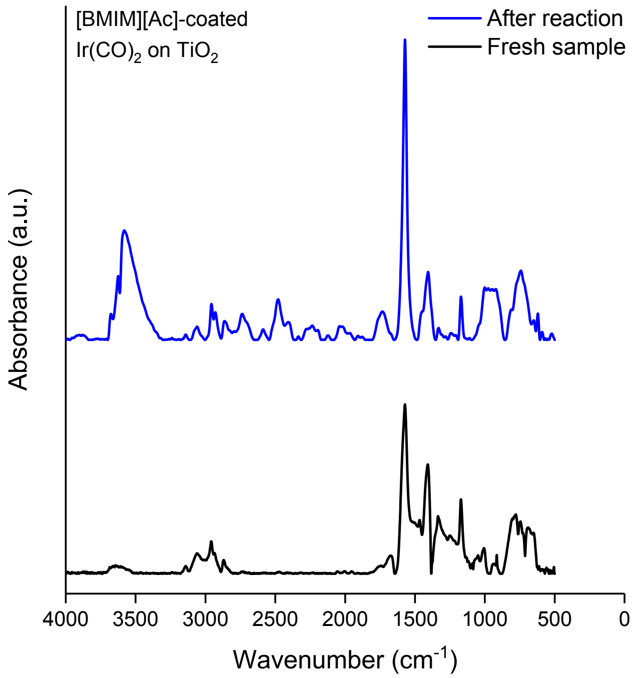

Figure S68. IR spectra of [BMIM][Ac]-coated Ir$(CO)_2$ complexes on TiO_2 before (black) and after (blue) ethylene exposure.

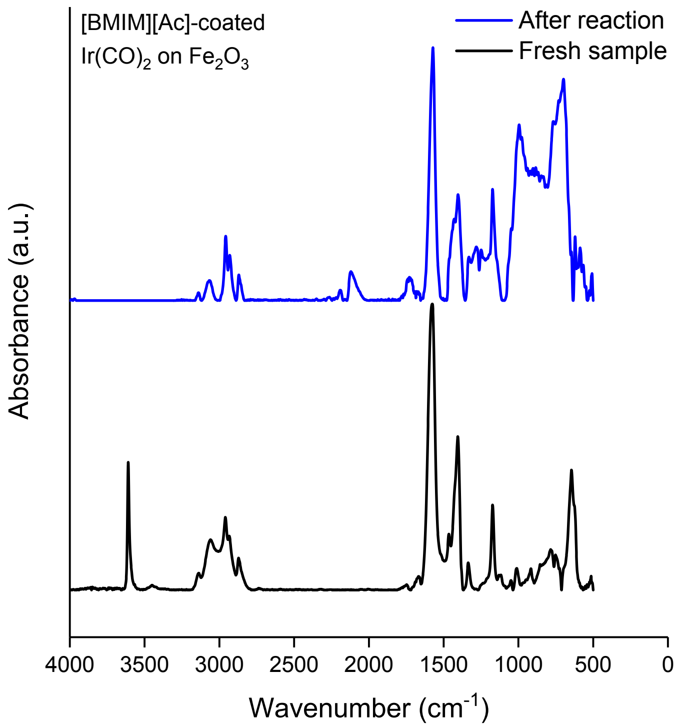

Figure S69. IR spectra of [BMIM][Ac]-coated $Ir(CO)_2$ complexes on Fe_2O_3 before (black) and after (blue) ethylene exposure.

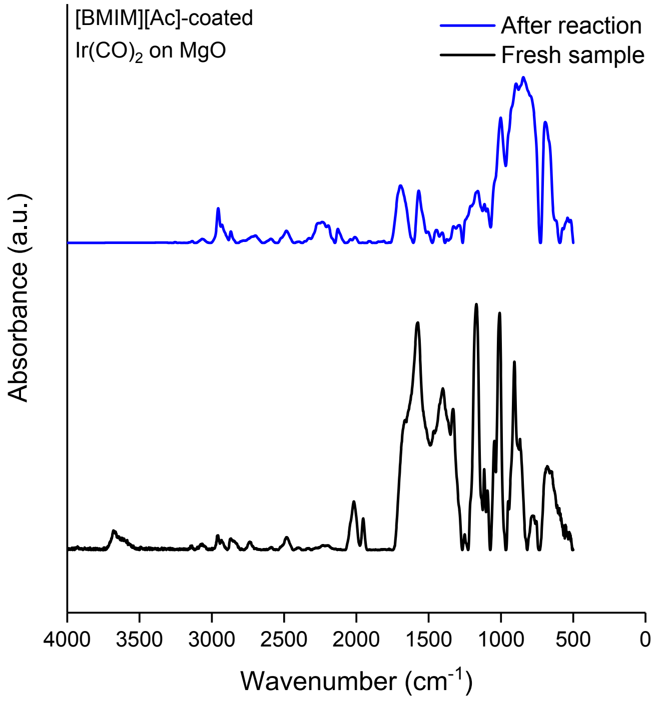

Figure S70. IR spectra of [BMIM][Ac]-coated $Ir(CO)_2$ complexes on MgO before (black) and after (blue) ethylene exposure.

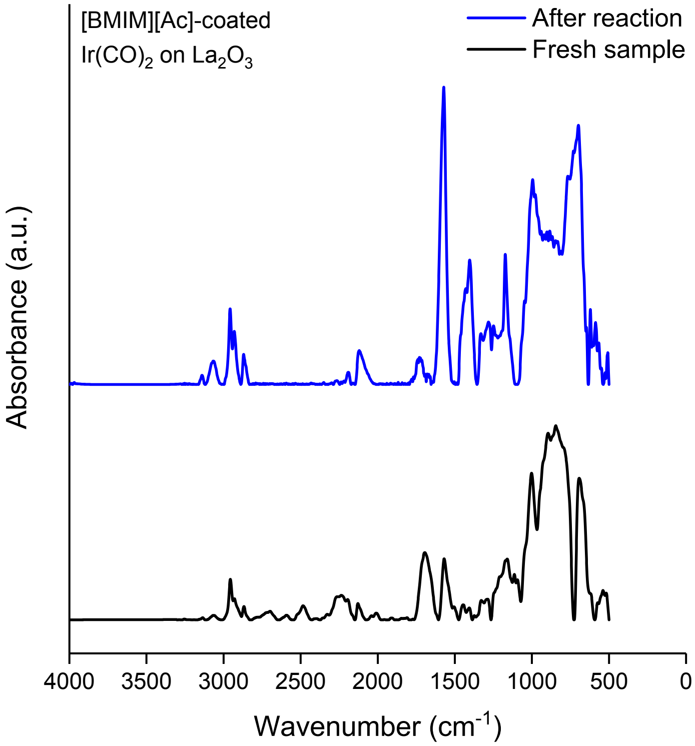

Figure S71. IR spectra of [BMIM][Ac]-coated $Ir(CO)_2$ complexes on La_2O_3 before (black) and after (blue) ethylene exposure.

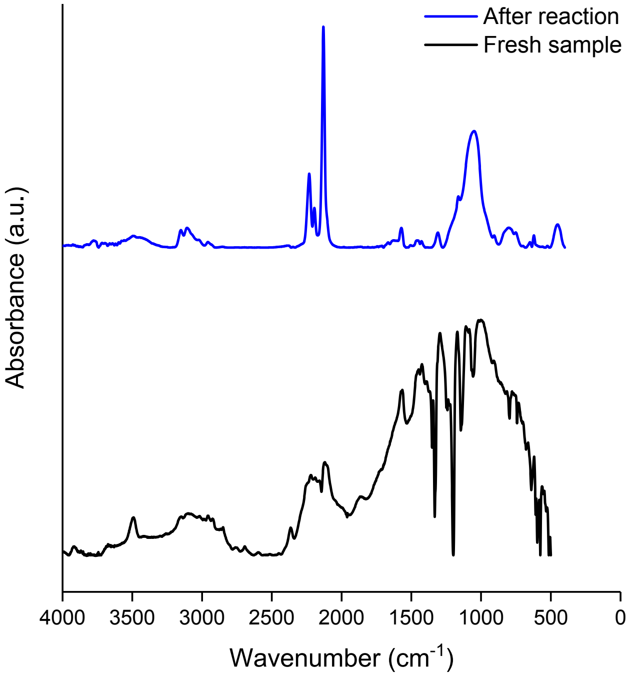

Figure S72. IR spectra of [CPMIM][DCA]-coated $Ir(CO)_2$ complexes on SiO_2 before (black) and after (blue) ethylene exposure.

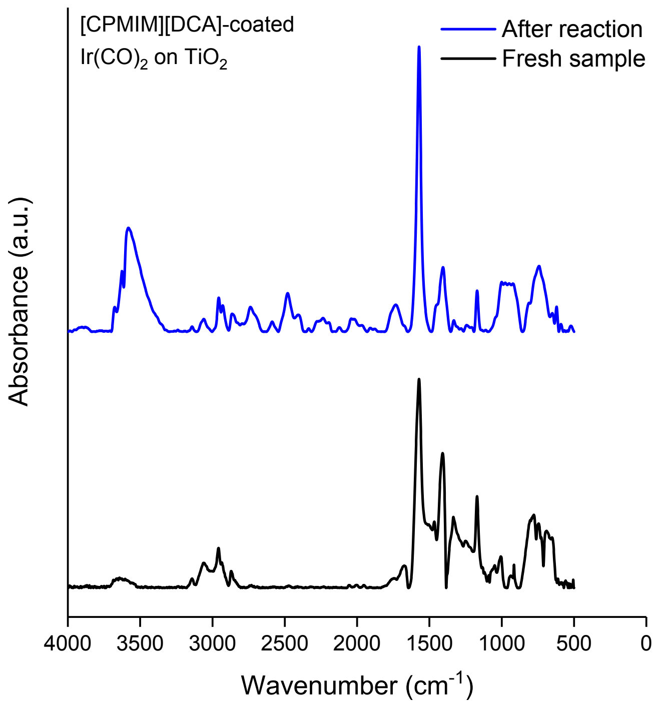

Figure S73. IR spectra of [CPMIM][DCA]-coated $Ir(CO)_2$ complexes on TiO_2 before (black) and after (blue) ethylene exposure.

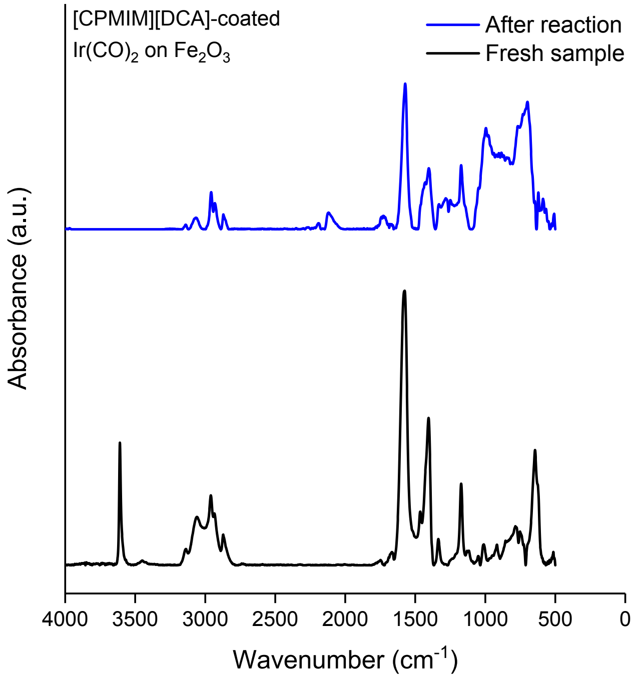

Figure S74. IR spectra of [CPMIM][DCA]-coated $Ir(CO)_2$ complexes on Fe_2O_3 before (black) and after (blue) ethylene exposure.

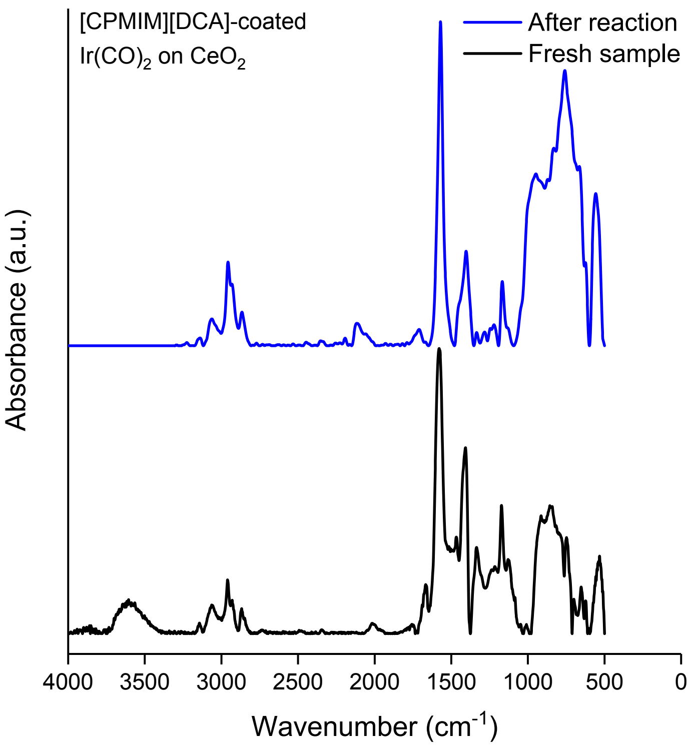

Figure S75. IR spectra of [CPMIM][DCA]-coated $Ir(CO)_2$ complexes on CeO_2 before (black) and after (blue) ethylene exposure.

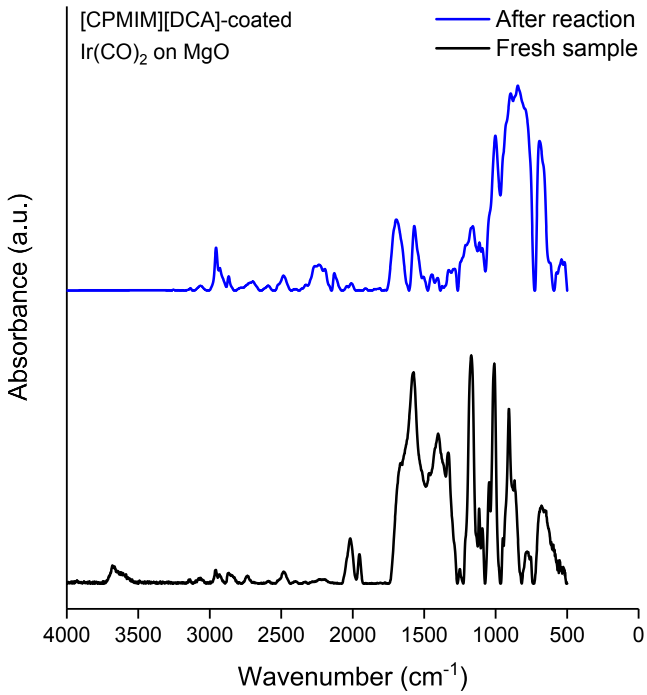

Figure S76. IR spectra of [CPMIM][DCA]-coated $Ir(CO)_2$ complexes on MgO before (black) and after (blue) ethylene exposure.

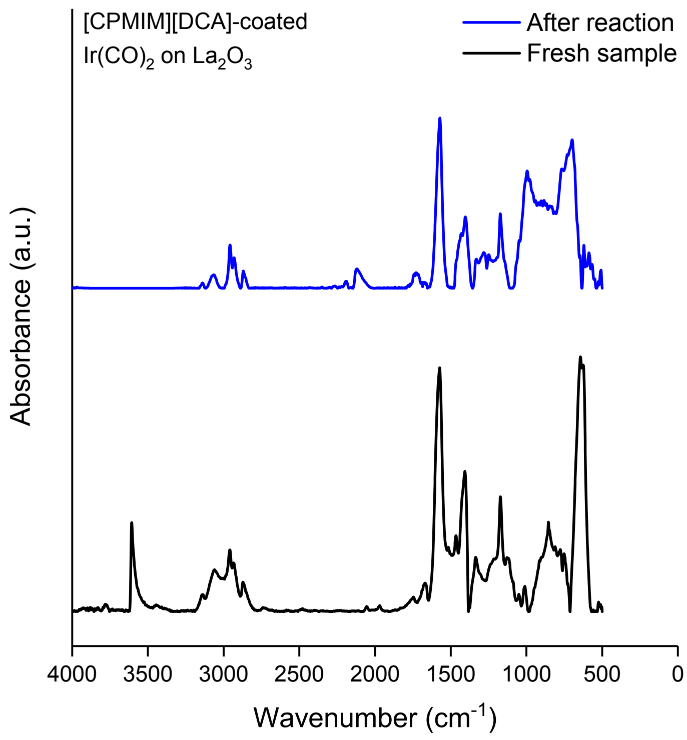

Figure S77. IR spectra of [CPMIM][DCA]-coated $Ir(CO)_2$ complexes on La_2O_3 before (black) and after (blue) ethylene exposure.

Following the activation of each supported iridium complex by reaction with ethylene, it was cooled to 333 K in flowing helium before the start of the catalytic reaction by introduction of a stream of H_2 + 1,3-butadiene in a molar ratio of 2.0 at a space velocity ensuring operation under differential conversion conditions (conversions <2%). Figure S78 illustrates that 1,3-butadiene conversion in the presence of [BMIM][BF_4]-coated $Ir(CO)_2$/γ-MgO remains almost constant during the reaction in the flow reactor for 3 h on stream.

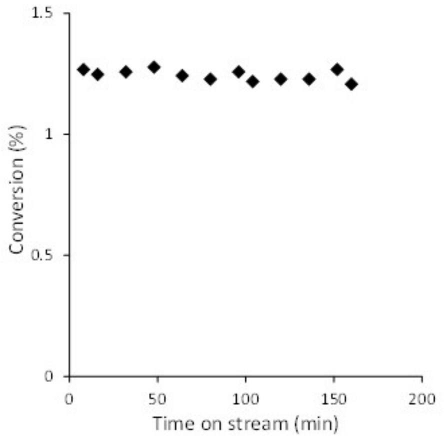

Figure S78. Change in 1,3-butadiene conversion with time on stream on a representative sample, [BMIM][BF_4]-coated $Ir(CO)_2/SiO_2$ at an H_2/BD ratio of 2.0 at 333 K and 1 atm.

Table S6. Selectivity and TOF values measured at steady-state and differential conversions (<2%) characterizing partial hydrogenation of 1,3-butadiene (BD) at an H_2/BD feed molar ratio of 2.0 at 333 K and 1 atm catalyzed by activated uncoated and IL-coated iridium complexes supported on metal oxides with various electron-donor characteristics.

**Supported $Ir(CO)_2$ complex**	**TOF × 10^3 ((mole of BD converted) × (Ir site × s)$^{-1}$)**	**Selectivity (%)**			
		**Butane**	***trans*-2-butene**	**1-butene**	***cis*-2-butene**
$Ir(CO)_2/SiO_2$	9.6	58	11	21	10
$Ir(CO)_2/TiO_2$	4.8	51	15	25	8
$Ir(CO)_2/Fe_2O_3$	4.7	30	24	30	15
$Ir(CO)_2/CeO_2$	2.3	36	20	29	16
$Ir(CO)_2/MgO$	2.5	28	24	33	16
$Ir(CO)_2/La_2O_3$	3.3	19	30	40	10
[BMIM][BF_4]-coated $Ir(CO)_2/SiO_2$	1.2	27	17	41	13
[BMIM][BF_4]-coated $Ir(CO)_2/TiO_2$	0.9	26	23	38	13
[BMIM][BF_4]-coated $Ir(CO)_2/Fe_2O_3$	1.4	21	26	33	20
[BMIM][BF_4]-coated $Ir(CO)_2/CeO_2$	0.8	16	22	47	15
[BMIM][BF_4]-coated $Ir(CO)_2/MgO$	0.7	20	26	40	14
[BMIM][BF_4]-coated $Ir(CO)_2/La_2O_3$	1.8	16	24	46	15
[BMIM][Ac]-coated $Ir(CO)_2/SiO_2$	0.5	7	45	18	31
[BMIM][Ac]-coated $Ir(CO)_2/TiO_2$	1.8	5	36	38	21
[BMIM][Ac]-coated $Ir(CO)_2/Fe_2O_3$	1.9	15	31	37	17
[BMIM][Ac]-coated $Ir(CO)_2/CeO_2$	1.2	14	28	38	20
[BMIM][Ac]-coated $Ir(CO)_2/MgO$	0.9	16	27	39	18
[BMIM][Ac]-coated $Ir(CO)_2/La_2O_3$	1.5	15	31	36	18
[CPMIM][DCA]-coated $Ir(CO)_2/SiO_2$	9.3	78	6	12	4
[CPMIM][DCA]-coated $Ir(CO)_2/TiO_2$	4.2	57	11	23	9
[CPMIM][DCA]-coated $Ir(CO)_2/Fe_2O_3$	4.6	51	13	27	9
[CPMIM][DCA]-coated $Ir(CO)_2/CeO_2$	2.2	46	17	26	11
[CPMIM][DCA]-coated $Ir(CO)_2/MgO$	1.6	36	24	31	10
[CPMIM][DCA]-coated $Ir(CO)_2/La_2O_3$	1.2	23	29	36	12

REFERENCES

1 S. Kawi and B. C. Gates, *Inorg. Chem.,* 1992, **31,** 2939-2947.

2 A. S. Hoffman, C.-Y. Fang, and B. C. Gates, *J. Phys. Chem. Lett.,* 2016, **7,** 3854-3860.

3 A. Uzun and B. C. Gates, *J. Am. Chem. Soc.,* 2009, **131,** 15887-15894.

4 A. Uzun, V. A. Bhirud, P. W. Kletnieks, J. F. Haw, and B. C. Gates, *J. Phys. Chem. C,* 2007, **111,** 15064-15073.

5 J. Lu, C. Aydin, N. D. Browning, and B. C. Gates, *Langmuir,* 2012, **28,** 12806-12815.

6 M. Mihaylov, E. Ivanova, F. Thibault-Starzyk, M. Daturi, L. Dimitrov, and K. Hadjiivanov, *J. Phys. Chem. B,* 2006, **110,** 10383-10389.

7 M. Babucci, C.-Y. Fang, A. S. Hoffman, S. R. Bare, B. C. Gates, and A. Uzun, *ACS Catal.,* 2017, **7,** 6969-6972.

8 C. Martinez-Macias, P. Serna, and B. C. Gates, *ACS Catal.,*2015, **5,** 5647-5656.

9 A. Uzun and B. C. Gates, *Angew. Chem. Int. Ed.,* 2008, **47,** 9245-9248.

10 C. Martinez-Macias, M. Chen, D. A. Dixon, and B. C. Gates, *Chem. Eur. J.,* 2015, **21,** 11825-11835.
